# Development of a multi-epitope vaccine from outer membrane proteins and identification of novel drug targets against *Francisella tularensis*: an *In Silico* approach

**DOI:** 10.3389/fimmu.2025.1479862

**Published:** 2025-04-03

**Authors:** Safoura Moradkasani, Saber Esmaeili, Mohammad Reza Asadi Karam, Ehsan Mostafavi, Behzad Shahbazi, Amir Salek Farrokhi, Mohsen Chiani, Farzad Badmasti

**Affiliations:** ^1^ WHO Collaborating Centre for Vector-Borne Diseases, Department of Epidemiology and Biostatistics, Research Centre for Emerging and Reemerging Infectious Diseases, Pasteur Institute of Iran, Tehran, Iran; ^2^ Student Research Committee, Pasteur Institute of Iran, Tehran, Iran; ^3^ National Reference Laboratory for Plague, Tularemia and Q Fever, Research Centre for Emerging and Reemerging Infectious Diseases, Pasteur Institute of Iran, Akanlu, KabudarAhang, Hamadan, Iran; ^4^ Department of Molecular Biology, Pasteur Institute of Iran, Tehran, Iran; ^5^ School of Pharmacy, Semnan University of Medical Sciences, Semnan, Iran; ^6^ Nervous System Stem Cells Research Center, Semnan University of Medical Sciences, Semnan, Iran; ^7^ Department of Immunology, Pasteur Institute of Iran, Tehran, Iran; ^8^ Department of Nanobiotechnology, Pasteur Institute of Iran, Tehran, Iran; ^9^ Department of Bacteriology, Pasteur Institute of Iran, Tehran, Iran

**Keywords:** *Francisella tularensis*, tularemia, multi-epitope vaccine, reverse vaccinology, drug target

## Abstract

**Background:**

*Francisella tularensis* is a category A potential thread agent, making the development of vaccines and countermeasures a high priority. Therefore, identifying new vaccine candidates and novel drug targets is essential for addressing this significant public health concern.

**Methods:**

This study presents an *in silico* analysis of two strategies against *F. tularensis* infection: the development of a multi-epitope vaccine (MEV) and the identification of novel drug targets. Outer membrane proteins (OMPs) were predicted using subcellular localization tools and immunogenicity was evaluated using a reverse vaccinology pipeline. Epitopes from these OMPs were combined to create candidate MEV for prophylactic protection. Concurrently, cytoplasmic proteins were subjected to rigorous analysis to identify potential novel drug targets.

**Results:**

Of 1,921 proteins, we identified 12 promising protein vaccine candidates from *F. tularensis* OMPs and proposed a multi-epitope vaccine (MEV) designed using seven immunodominant epitopes derived from four of these OMPs, including two hypothetical proteins (WP_003026145.1 and WP_003029346.1), an OmpA family protein (WP_003020808.1), and PD40 (WP_003021546.1). In addition, we proposed 10 novel drug targets for *F. tularensis*: Asp-tRNA (Asn)/Glu-tRNA (Gln) amidotransferase subunit GatC (WP_003017413.1), NAD(P)-binding protein (WP_042522581.1), 30S ribosomal protein S16 (WP_003023081.1), Class I SAM-dependent methyltransferase (WP_003022345.1), haloacid dehalogenase (WP_003014157.1), uroporphyrinogen-III synthase (WP_003022220.1), and four hypothetical proteins (WP_003017784.1, WP_003020080.1, WP_003020066.1, and WP_003022350.1).

**Conclusion:**

This study designed an MEV and proposed novel drug targets to address tularemia, offering broad protection against various *F. tularensis* strains. MEV, with favorable physicochemical properties, showed strong potential through molecular docking and dynamic simulations. Immune simulations suggest that it may elicit robust responses against pathogens. The identification of novel drug targets can lead to the discovery of new antimicrobial agents. However, further *in vitro* and *in vivo* studies are required to validate their efficacy and capability.

## 1 Introduction

Tularemia is an acute zoonotic disease caused by *Francisella tularensis*, a Gram-negative intracellular coccobacillus that poses a significant threat to human health (1). The disease is primarily caused by two subspecies: *F. tularensis* subsp. *tularensis* (type A) and *F. tularensis* subsp. *holarctica* (type B), with type A being the most virulent (2). Without antibiotic treatment, tularemia can lead to a mortality rate as high as 30% (3). Recognizing its potential as a bioweapon, the Centers for Disease Control and Prevention (CDC) classifies *F. tularensis* as a Category A bioterrorism agent, underscoring the urgent need for effective medical countermeasures (3). The main antibiotics used to treat tularemia include fluoroquinolones, tetracyclines, and aminoglycosides (4). However, *F. tularensis* exhibits resistance to several commonly used antibiotics including penicillin, polymyxin B, erythromycin, azithromycin, and carbapenems (4, 5). Given these challenges, ongoing research on novel molecular structures with unique mechanisms of action, supported by microbial genome analysis, is essential for identifying new drug targets, particularly for pathogens that are difficult to culture (6).

Despite extensive efforts, an approved vaccine for human tularemia remains elusive, primarily because of the complex immune evasion strategies employed by *F. tularensis* (7). Although B-cells generate antibodies, these are insufficient for complete protection. The intracellular nature of the bacterium significantly limits the effectiveness of the humoral response, emphasizing the critical role of T-cells, particularly the CD4+ and CD8+ subsets, in providing long-lasting immunity. T-cells contribute to bacterial clearance through cytokine production and the coordination of the immune response, underscoring the need for robust T-cell-mediated immunity. Given the limitations of traditional vaccines and the intricate immune response required to combat *F. tularensis* effectively, the development of a subunit vaccine that elicits strong coordinated T-cell and B-cell responses is essential. This strategy focuses on harnessing key immunodominant epitopes to activate both arms of the immune system to counteract the immune evasion mechanisms of bacteria and deliver comprehensive protection (8). Although conventional multi-antigen vaccines have limitations, peptide-based multi-epitope vaccines (MEVs) represent significant advancements. By leveraging *in silico* methods, MEVs can efficiently identify and target immunodominant epitopes, thereby addressing many shortcomings inherent in traditional vaccine approaches (9, 10). Traditional vaccine development is typically time-consuming and costly and requires extensive *in vivo* and *in vitro* testing (11). However, advances in computational biology and bioinformatics have revolutionized this process, enabling the rapid design of highly effective vaccine constructs and significantly reducing dependence on labor-intensive traditional laboratory methods (12). Reverse vaccinology is a transformative computational approach that leverages genomic data to design vaccine candidates, without the need for pathogen cultivation. By analyzing protein sequences, this method identifies multiple epitopes that trigger both cellular and humoral immune responses, while minimizing side effects (13, 14). Unlike traditional methods, it uncovers both known and novel antigens, opens new pathways for immune interventions, and enhances our understanding of pathogen-host interactions (15). Epitope-based immune-derived vaccines (IDVs) offer significant safety and efficacy advantages over conventional vaccines, particularly against complex pathogens, such as *F. tularensis*, which employs advanced immune evasion strategies. IDVs enhance T-cell responses, which are crucial for long-lasting immunity (16). Reverse vaccinology has successfully prioritized and designed vaccine targets for various pathogens (17–21). MEVs have emerged as effective solutions that incorporate key epitopes to stimulate stronger immune responses, thereby offering superior protection against infectious diseases (22).

In light of these considerations, our study proposes a comprehensive, two-pronged approach to combat tularemia. The first objective is to design an MEV that not only elicits a strong immune response but also effectively targets the unique challenges posed by this intracellular pathogen. By focusing on key immunodominant epitopes, we aimed to activate both T-cell and B-cell pathways, creating a balanced immune response that addresses the complexities of immune evasion by *F. tularensis*. The second objective was to identify new drug targets, potentially leading to novel therapeutic options for the treatment of tularemia. This integrated approach holds significant promise for the development of effective vaccines and therapeutics. By combining cutting-edge computational methods with deep immunological insights, our research aims to pave the way for innovative strategies for preventing and treating tularemia.

## 2 Materials and methods

### 2.1 Design of multi-epitope subunit

#### 2.1.1 Genomic sequence retrieval

A total of 686 *F. tularensis* strains with complete genome sequences were retrieved from the GenBank database (https://www.ncbi.nlm.nih.gov/genbank/), and their proteomes were extracted for core/pan-genome analysis with BPGA software (Bacterial Pan Genome Analysis Tool), version 1.3 (23) based on the USEARCH algorithm. The BPGA analysis of these 686 strains revealed that *F. tularensis* had an open pan-genome and there was a significant genomic diversity within *F. tularensis*. Based on these findings, the *F. tularensis* strain 2017317779 was selected as the reference strain. This strain was classified as *F. tularensis* type A and was isolated from the lung tissue of a patient with tularemia in the USA in 2017 (24). The protein-coding sequences of this strain were annotated and used for further analysis.

#### 2.1.2 Prediction of subcellular localization

The subcellular localization of all *F. tularensis* strain 2017317779 (GenBank: CP073122), used as a reference strain, was predicted using both of PSORTb v3.0.3 (www.psort.org/psortb/) and CELLO version 2.5 (http://cello.life.nctu.edu.tw/) for more accuracy. The results were confirmed with the TMHMM Server v2.0 web tool (https://services.healthtech.dtu.dk/service.php?TMHMM-2.0) (25–27). At this stage, only the surface-exposed proteins including extracellular and outer membrane proteins (OMPs) of *F. tularensis* strain 2017317779 (GenBank: CP073122) were considered for further analysis.

#### 2.1.3 Determination of the overall antigenicity and allergenicity

The antigenicity of the putative immunogenic targets was assessed using the VaxiJen web tool (http://www.ddg-pharmfac.net/vaxijen/VaxiJen/VaxiJen.html), with a cutoff value of ≥ 0.5. This tool utilizes Auto-Cross Covariance (ACC) transformation, converts sequences into uniform vectors based on the chemical properties of proteins, and provides predictions with an accuracy ranging from 70% to 89% (28). To evaluate allergenicity, we employed the AlgPred 2.0 server (https://webs.iiitd.edu.in/raghava/algpred2/batch.html), using a cutoff value of ≥ 0.5. This server integrates six distinct methodologies: (i) IgE mapping, (ii) MEME/Mast motif analysis, (iii) support vector machine (SVM) modules based on both amino acid and dipeptide compositions, (iv) BLAST searches against allergen-representative proteins (ARPs), and (v) a hybrid approach that combines all parameters. While MEME/Mast analysis, BLAST, and IgE mapping indicated non-allergenicity, the SVM modules and hybrid approach suggested potential allergenicity. To ensure a comprehensive evaluation, we performed an additional allergenicity assessment using AllerTOP v2.0 (https://www.ddg-pharmfac.net/AllerTOP/), which corroborated the non-allergenic nature of these proteins. Based on the combined results from both servers, we concluded that vaccine candidates are likely non-allergenic (29, 30).

#### 2.1.4 Homology analysis of immunogenic targets against the human proteome

All selected proteins were analyzed for sequence similarity to the human proteome (*Homo sapiens*, Taxid: 9606) using the PSI-BLAST tool in the BLASTp database (https://blast.ncbi.nlm.nih.gov/Blast.cgi?SIDE=protein). Proteins with significant similarities were excluded to prevent cross-reactivity.

#### 2.1.5 Prevalence of putative immunogenic targets among *F. tularensis* strains

The prevalence and conservation of proteins among these strains were assessed to induce a strong immune response against all *F. tularensis* strains. Homologs of each immunogenic target in the 686 *F. tularensis* strains were retrieved using BLASTp and aligned using MegaX software (31). Proteins with a prevalence > 90% were selected for further analysis (32).

#### 2.1.6 Linear B-cell, T-cell epitopes, and quartile scoring

The VICMpred database (https://webs.iiitd.edu.in/raghava/vicmpred/submission.html) was used to categorize the functional roles of the proteins into four distinct classes: virulence, cellular processes, metabolic molecules, and unknown (33). To identify linear B-cell epitopes in the selected proteins, we used the BepiPred-2.0 tool (https://services.healthtech.dtu.dk/service.php?BepiPred-2.0), with a threshold of ≥ 0.6 (34). The B-cell epitope ratio was calculated by dividing the number of amino acids in the identified epitopes by the total amino acid count for each protein. Subsequently, TepiTool (http://tools.iedb.org/tepitool/), a resource from the Immune Epitope Database (IEDB), was used to predict binding sites for human MHC I and MHC II. For MHC I, binding sites were chosen from the top 5% of peptides, prioritizing those prevalent in the reference HLA allele set for the normal human population (35). The MHC II binding site ratio was calculated by dividing the number of MHC II binding sites by the total amino acid content in each protein. A quartile scoring method was then applied to evaluate each protein based on three indicators: functional class, MHC II binding site ratio, and B-cell epitope ratio. The overall score for each protein was calculated by summing the individual scores of these indicators. Finally, proteins in the top quartile with the highest cumulative scores were selected.

#### 2.1.7 Protein domain search and predicting of physiochemical characteristics of immunogenic targets

The Conserved Domain Database (CDD) (https://www.ncbi.nlm.nih.gov/Structure/cdd/cdd.shtml) and EggNOG (http://eggnog5.embl.de/#/app/home) were used to identify the functional domains and classification of the proteins. CDD, a part of the NCBI Entrez query system, annotates the location of conserved domains in protein sequences (36, 37). The Expasy ProtParam online tool (https://web.expasy.org/protparam/) was used to calculate the molecular weights and theoretical isoelectric points of the selected proteins (38).

#### 2.1.8 Tertiary structure prediction and characterization of the conformational B-cell epitopes

The Swiss Model (https://swissmodel.expasy.org/) was used to predict the 3D structures of the selected proteins (39). To assess and validate the quality and stability of these models, we used the ProSA-web server (https://prosa.services.came.sbg.ac.at/prosa.php) and ERRAT (https://saves.mbi.ucla.edu/results?Job%20=1243713,%20p=errat) to detect potential errors (40, 41). Conformational B-cell epitopes were identified using the ElliPro server (http://tools.iedb.org/ellipro/) at a threshold of ≥ 0.8. The predicted epitopes were then visualized on the protein surface using Jmol software, with each epitope represented by different colors for clarity (42).

#### 2.1.9 Analysis of protein-protein interactions

The STRING software (https://string-db.org/) server was used to analyze the interaction network of proteins with unknown functions (43). In this analysis, interactions with high confidence scores > 0.7 were considered to minimize false-positive and false-negative results (44).

#### 2.1.10 Prediction and selection of optimal epitopes for vaccine target

B-lymphocytes are crucial in humoral immunity and produce antibodies that detect and neutralize pathogens. To identify potential vaccine targets, the selected protein sequences were scanned for linear B-cell epitopes using the Kolaskar-Tongaonkar algorithm within the Antibody Epitope Prediction tool hosted on the Immune Epitope Database (IEDB) analysis server (http://tools.iedb.org) (35). The predicted B-cell epitopes were evaluated for MHC I processing compatibility, focusing on the most prevalent HLA alleles in Iran, using the IEDB server (35). The VaxiJen web tool (http://www.ddg-pharmfac.net/vaxijen/VaxiJen/VaxiJen.html) was used to predict the antigenicity of putative immunogenic epitopes, with a cut-off value of ≥ 0.5. Additionally, AlgPred 2.0 serve (https://webs.iiitd.edu.in/raghava/algpred2/batch.html) was employed with a cut-off value ≥ 0.5 to investigate the allergenicity of these epitopes. Epitope conservation and hydropathicity were determined using the IEDB analysis server (http://tools.iedb.org/conservancy/). Epitopes that were antigenic and non-allergenic showed > 90% conservation, and the lowest hydropathicity scores were selected as potential candidates for subunit vaccine development.

#### 2.1.11 Structural construction and validation of the vaccine candidate

Effective vaccine design requires appropriate antigenic peptide folding and linker incorporation to maintain epitope integrity, prevent unintended junctional epitopes, and enhance immunogenicity. Without suitable linkers, multi-epitope vaccines may generate unwanted proteins or epitopes, reducing their effectiveness and stability (45). To construct an MEV against *F. tularensis*, linear B-cell epitopes were fused using flexible GPGPG linkers. These flexible linkers, composed of small amino acids, such as glycine and serine, ensure adequate separation between epitope domains and minimize junctional epitope formation, allowing the protein domains to move freely and facilitating proper protein folding. Flexible linkers are also advantageous for enhancing antibody accessibility to epitopes and improving the folding efficiency (46, 47). To optimize antigenicity, epitope shuffling techniques were used to identify the best epitope arrangement with the highest antigenicity score. The 3D structures of the selected proteins were predicted using the Swiss Model (https://swissmodel.expasy.org/) (39). The quality and stability of the 3D models were further evaluated using the ProSA-web (https://prosa.services.came.sbg.ac.at/prosa.php) and ERRAT (https://saves.mbi.ucla.edu/results?Job%20=1243713,%20p=errat) servers to detect structural errors (40, 41). Furthermore, the affinities of the multi-epitope vaccine against human Toll-like receptor 2 (TLR2, PDB: 2Z7X), human Toll-like receptor 4 (TLR4 PDB: 3FXI), HLA-DR-B (PBD: 3PDO_B), and HLA-A chain A (PDB:8XG2_A) were evaluated using the HDOCK web tool (http://hdock.phys.hust.edu.cn/) (48). The interactions of the docked complexes were visualized and validated using the PDBsum server (https://www.ebi.ac.uk/thornton-srv/databases/pdbsum/) (49).

#### 2.1.12 Evaluation of allergenicity, antigenicity, solubility, and physicochemical properties of MEV

To assess the allergenicity of the vaccine construct, we utilized the AlgPred 2.0 server (https://webs.iiitd.edu.in/raghava/algpred2/batch.html) with a cut-off value ≥ 0.5. Allergenicity analysis was performed using AllerTOP v. 2.0 (https://www.ddg-pharmfac.net/AllerTOP/). Both tools consistently indicated that the vaccine construct is non-allergenic, thus mitigating the risk of allergic reactions during vaccination protocols (29, 30). Next, we evaluated the antigenicity of MEV using the VaxiJen server tool (http://www.ddg-pharmfac.net/vaxijen/VaxiJen/VaxiJen.html) (28). Subsequently, the Protein-Sol server (https://protein-sol.manchester.ac.uk/) was employed to predict the solubility of the engineered MEV, where scores above 0.45 indicate solubility (50). Further characterization of MEV included the prediction of major physicochemical characteristics using the ExPASy ProtParam web tool (https://web.expasy.org/protparam/) (38). This tool provides insights into the molecular weight (Mw), theoretical isoelectric point (pI), amino acid composition, *in vitro* and *in vivo* protein half-life, aliphatic index, instability index, and grand average of hydropathicity (GRAVY) score (51). These parameters are essential to understand the stability, solubility, and potential effectiveness of MEVs as vaccine candidates.

#### 2.1.13 Reverse translation and *in silico* cloning

To ensure codon compatibility and optimize the vaccine construct for expression, we used the Java Codon Adaptation Tool (https://www.jcat.de/) (52). This tool refines codon usage specifically for expression in *Escherichia coli* K12, following the recommended guidelines. For optimal expression, it is important that the protein sequence maintains a GC content between 30–70% and achieves a Codon Adaptation Index (CAI) value above 0.8 (52). Once the sequence was optimized, we performed *in silico* cloning of the finalized MEV model using the SnapGene v7.2 software (https://www.snapgene.com/resources). This software enabled a virtual cloning process, allowing insertion of the optimized vaccine construct into a vector suitable for expression studies and preparation for experimental validation.

#### 2.1.14 Simulations for evaluating the immune response of the vaccine construct

The C-ImmSim tool (https://kraken.iac.rm.cnr.it/C-IMMSIM/) employs position-specific scoring matrices to simulate and predict the intensity of immune responses triggered by vaccines over different time intervals (53). This model helps researchers to understand how vaccine timing and administration influence immune dynamics, which is crucial for optimizing vaccination strategies and evaluating the effectiveness of vaccine candidates.

#### 2.1.15 Molecular dynamics simulation

Molecular dynamics (MD) simulations provide crucial insights into the structural stability and functionality of vaccines in a simulated cytosolic environment. In this study, we explored the interactions between MEV, TLR2, and TLR4 using a 100-nanosecond MD simulation conducted using GROMACS version 2018. The MEV and receptors were situated in a dodecahedral solvent box containing TIP3P water molecules. To simulate physiological conditions, some water molecules were randomly substituted with sodium (Na^+^) and chloride (Cl^-^) ions. The system was then subjected to energy minimization, followed by equilibration under constant volume (NVT) and pressure (NPT) conditions to ensure system stability. Throughout the simulation, the particle mesh Ewald (PME) method was employed to compute electrostatic interactions, and LINCS constraints were applied to maintain the hydrogen bond lengths. This methodology enabled us to accurately simulate the interactions between MEV and TLRs over 100 nanoseconds (54).

### 2.2 Identification of novel drug targets

#### 2.2.1 Detection of cytoplasmic protein sequences

Cytoplasmic protein sequences of *F. tularensis* strain 2017317779 were determined using PSORTb version 3.0.3 (http://www.psort.org/psortb/) and CELLO v. 2.5 (http://cello.life.nctu.edu.tw/) (25–27). Given their crucial roles in cellular processes, cytoplasmic proteins are promising targets for small-molecule drug targeting (55).

#### 2.2.2 Selecting human non-similar protein sequences

The homology of the selected proteins to the human proteome (*Homo sapiens*, taxid:9606) was evaluated using PSI-BLAST (https://blast.ncbi.nlm.nih.gov/) to identify proteins similar to those in humans. Proteins that demonstrated significant similarities were excluded from further analysis. Additionally, the MITOMASTER database (http://mammag.web.uci.edu/twiki/bin/view/Mitomaster) was used to assess the similarity between selected proteins and human mitochondrial proteins (55). This precaution ensured that the chosen proteins were distinct from those present in human mitochondria, reinforcing their potential as candidates for further investigation.

#### 2.2.3 Identification of unique metabolic pathway proteins

The KAAS server from the Kyoto Encyclopedia of Genes and Genomes (KEGG) database (https://www.genome.jp/kaas-bin/kaas_main) and BLASTp analysis were employed to identify non-homologous to host proteins that are crucial for the study (56). Protein sequences with distinct pathways from the host were selected for analysis, ensuring that only the proteins involved in specific host pathways were included. This approach focuses on unique biological interactions and processes.

#### 2.2.4 Detection of essential proteins

The DEG 15.2 server contains all crucial proteins involved in key functions of bacterial life, as determined by experimental studies (57). Essential proteins were identified using Genome BLAST with a coverage > 90% and an identity > 80% from the DEG database (http://origin.tubic.org/deg/public/index.php/index).

#### 2.2.5 Identifying novel therapeutic targets

To highlight the uniqueness of the selected proteins as pharmacological targets, we analyzed the remaining candidates from earlier stages using the DrugBank database (https://www.drugbank.ca/structures/search/bonds/sequence) (58). This evaluation assessed their druggability and reinforced their potential as novel therapeutic candidates based on well-established criteria. The proteins were systematically screened against all known drug targets in DrugBank and those didn’t exhibit any proposed drugs, were classified as novel drug targets.

#### 2.2.6 Identification of proteins non-similar to the host microbiome

The aim of examining the lack of similarity between the *F. tularensis* metabolic pathway proteins and those from beneficial host microorganisms was to ensure their distinctiveness. We used the NCBI BLAST server for sequence comparisons (See **Supplementary Data 1** for details on gut microbiota) (59). Proteins exhibiting significant similarities with the host microbiome proteins were excluded from the study.

#### 2.2.7 Prediction of functional domains of novel drug targets

Functional domains of the proteins were identified using the Conserved Domain Database (CDD) (https://www.ncbi.nlm.nih.gov/Structure/cdd/cdd.shtml) based on the NCBI Entrez query and EggNOG (http://eggnog5.embl.de/#/app/home) (36, 37).

#### 2.2.8 Analysis of protein-protein interactions

To analyze the interaction network of hypothetical proteins related to distinct drug targets, STRING 11.5 server (https://string-db.org) was employed (43). Interactors with high confidence scores > 0.7 were included in the protein network to mitigate false-positive and false-negative results. Eliminating the query protein resulted in changes in the number of edges (interconnections) and nodes (interconnected proteins), highlighting its influence on biological processes in the organism (44).

## 3 Results

### 3.1 Design of MEV

#### 3.1.1 Sequence retrieval

**Figure 1:**
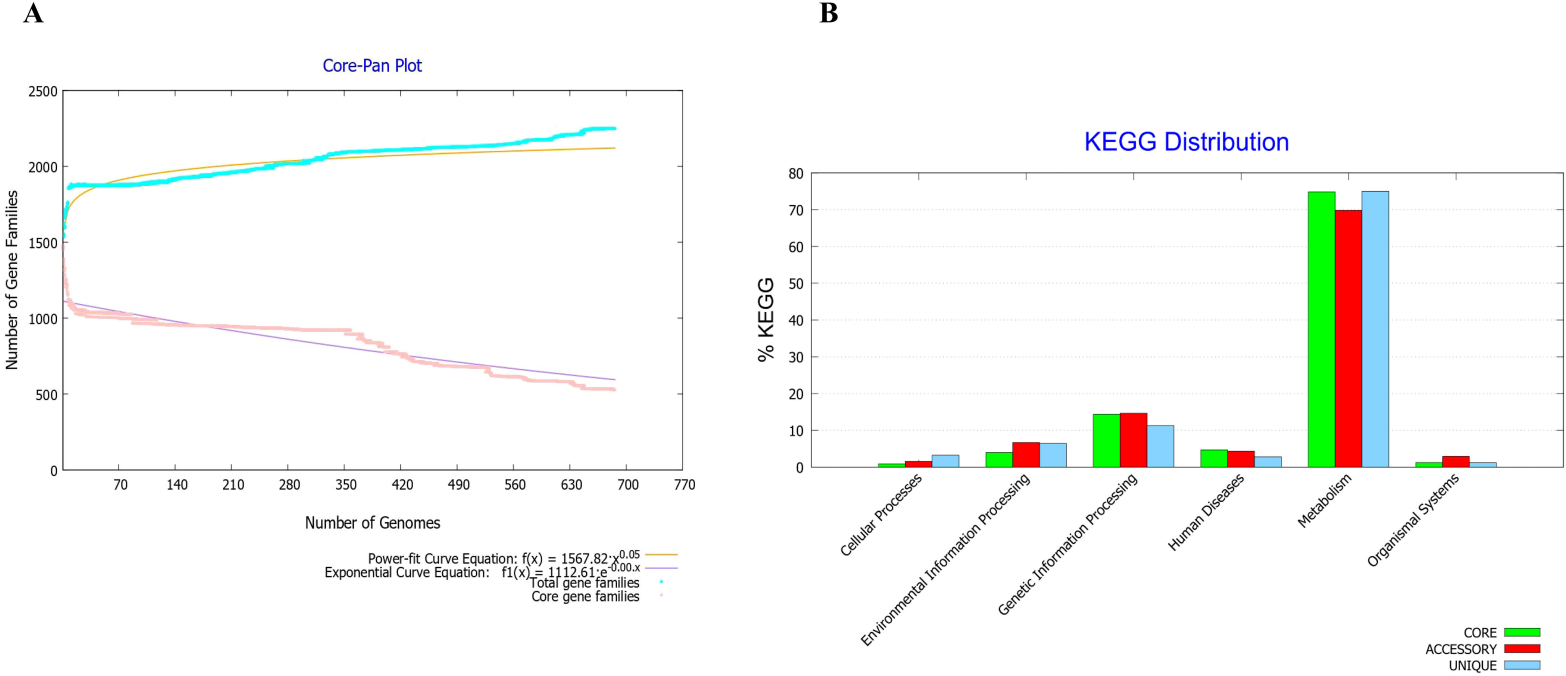
Core-proteome analysis of *F*. *tularensis* strains using BPGA software v 1.3. **(A)** The core-pan plot of 686 F*. tularensis* strains identified 2,249 core proteins. This analysis also demonstrated significant genomic diversity within the *F*. *tularensis* species. **(B)** Clusters of Orthologous Groups (COGs) analysis showed that the majority of core proteins are involved in key biological functions, including secondary metabolism, genetic and environmental information processing, cellular processes, organismal systems, and human disease pathways.

**Figure 2:**
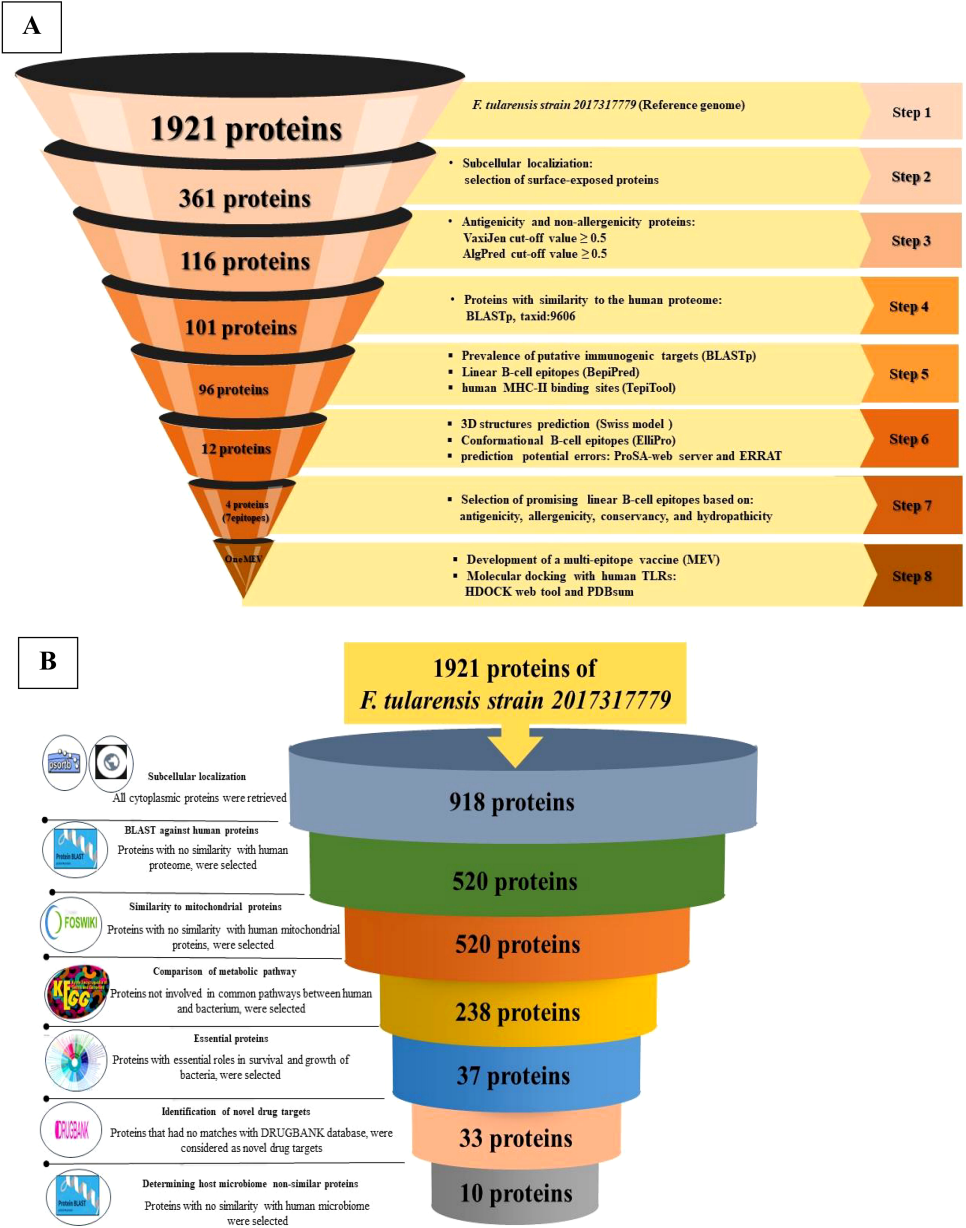
Schematic overview of the selection and validation process for putative immunogenic targets, MEV, and novel drug targets. **(A)** This panel illustrates the sequential flow of the reverse vaccinology strategy employed to design an MEV for *F*. *tularensis*. The schematic includes a comprehensive overview of the software, databases, web tools, and specific cut-off criteria utilized throughout the workflow. **(B)** This panel presents the workflow for identifying novel drug targets in *F*. *tularensis*. It delineates the various software applications, databases, web tools, and cut-off criteria that were systematically applied during the target identification process in *F*. *tularensis*.

The core-to-pan protein ratio was low and the core-pan plot appeared almost open (**Figure 1A**). Consequently, using the core proteome was deemed unsuitable, leading to the decision to utilize the entire genome of the *F. tularensis* strain 2017317779 (24). The KEGG distribution plot demonstrated that most core, accessory, and unique genes were involved in the metabolism of this pathogen (**Figure 1B**). Based on these findings, the proteome of the *F. tularensis* strain 2017317779 was retrieved from the UniProt database (http://www.uniprot.org/). The workflow for identifying new immunogenic targets and designing multi-epitope vaccines against *F. tularensis* is illustrated in **Figure 2A**.

#### 3.1.2 Prediction of subcellular localization

All 1,921 proteins in the reference strain were analyzed using PSORTb, CELLO, and TMHMM. In total, 361 proteins were identified as OMPs located in the extracellular space.

#### 3.1.3 Prediction of antigenicity and allergenicity

A total of 123 antigenic proteins were identified, of which 116 were determined to be non-allergenic.

#### 3.1.4 Selecting non-homologous proteins from the human proteome

All 116 proteins were evaluated for homology with *Homo sapiens* (taxid: 9606). This analysis revealed that 101 proteins were non-homologous, while 15 proteins were homologous; thus, 15 homologous proteins were excluded.

#### 3.1.5 Prevalence of putative immunogenic targets among circulating *F. tularensis* strains

The frequency of 101 selected proteins was assessed in the 686 F*. tularensis* strains. Five proteins exhibited a low prevalence (< 90%) among these strains and were subsequently excluded from the study.

#### 3.1.6 Quartile score-refined proteins: their conserved domains and physicochemical properties

**Figure 3:**
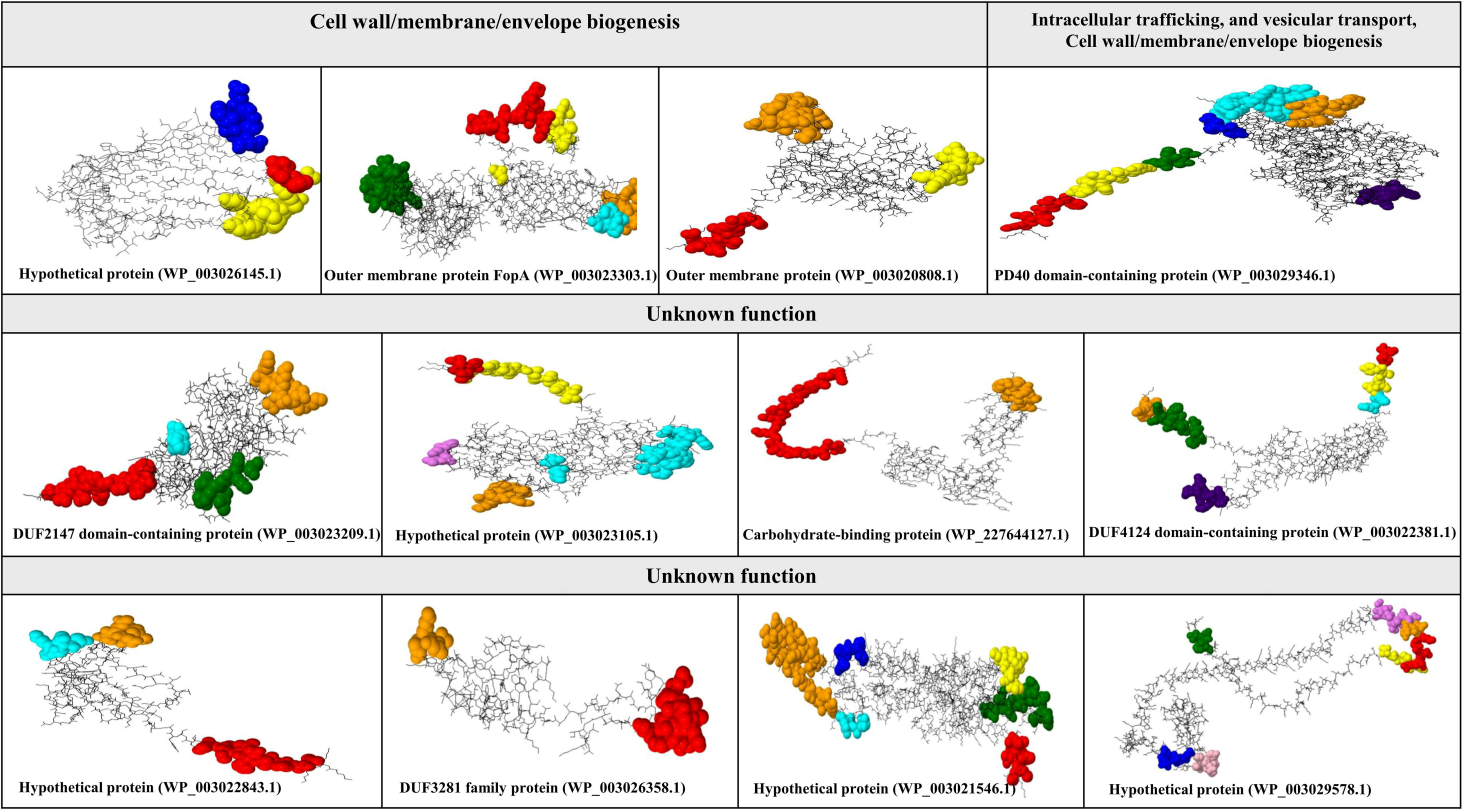
Characterization of conformational B-cell epitopes in putative immunogenic targets against *F. tularensis*. This figure illustrates the identification and mapping of conformational B-cell epitopes on the tertiary structures of potential immunogenic targets against *F. tularensis*. Conformational B-cell epitopes were determined using ElliPro and visualized with Jamal software. Each color represents a unique conformational epitope, allowing for easy differentiation and highlighting of the spatial distribution within the protein structure.

Using the quartile method, 12 immunogenic targets were identified from the 96 candidate proteins (**Figure 3**). These targets were scored within the top 25% of properties associated with successful vaccine development. Notably, four proteins were associated with cell wall, membrane, and envelope biogenesis, which are crucial for bacterial survival and serve as potential targets for immune attack. The identified proteins were FopA (WP_003023303.1), OmpA family protein (WP_003020808.1), and a hypothetical protein (WP_003026145.1). Notably, another hypothetical protein (WP_003029346.1) plays a dual role in intracellular trafficking and cell wall biogenesis, suggesting its potential involvement in immune responses. In addition, eight proteins with unknown functions were identified, including hypothetical proteins (WP_003023105.1, WP_003029578.1, WP_003022843.1), PD40 (WP_003021546.1), DUF2147 (WP_003023209.1), DUF3281 (WP_003026358.1), DUF4124 (WP_003022381.1), and a carbohydrate-binding protein (WP_227644127.1) (**Figure 3**). All identified proteins had molecular weights less than 110 kDa.

#### 3.1.7 Tertiary structure prediction and characterization of conformational B-cell epitopes

The structures predicted by SWISS-MODEL were validated using Verify 3D, PROSA, and ERRAT analyses, which confirmed the accurate folding of all 12 proteins (**Supplementary Data 2**). Conformational epitopes were identified and visualized based on the predicted tertiary structures. The number of conformational epitopes for the 12 putative immunogenic targets is as follows: hypothetical protein (WP_003026145.1) (three epitopes), outer membrane protein FopA (WP_003023303.1) (five epitopes), OmpA family protein (WP_003020808.1) (three epitopes), hypothetical protein (WP_003029346.1) (seven epitopes), DUF2147 (WP_003023209.1) (four epitopes), hypothetical protein (WP_003023105.1) (five epitopes), carbohydrate-binding protein (WP_227644127.1) (two epitopes), DUF4124 (WP_003022381.1) (six epitopes), hypothetical protein (WP_003022843.1) (three epitopes), DUF3281 (WP_003026358.1) (two epitopes), PD40 (WP_003021546.1) (six epitopes), and hypothetical protein (WP_003029578.1) (seven epitopes). See **Figure 3**. Additional details are provided in **Supplementary Table 1**.

#### 3.1.8 Protein-protein interaction networks

**Figure 4:**
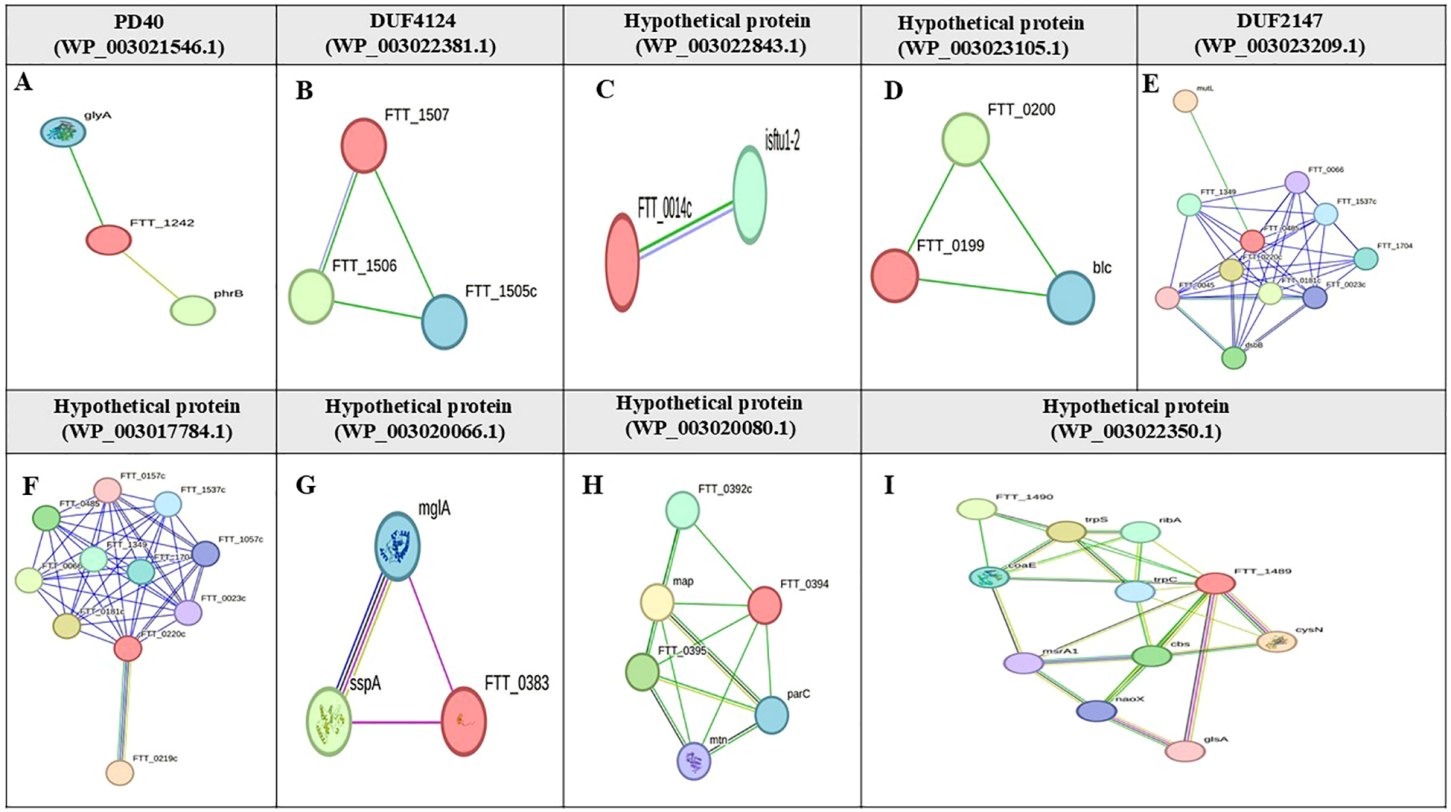
Protein-protein interaction network of hypothetical proteins in *F*. *tularensis*. This figure shows the STRING interaction networks for various hypothetical proteins in *F*. *tularensis*: **(A)** PD40 (WP_003021546.1), **(B)** DUF4124 (WP_003022381.1), **(C)** Hypothetical protein (WP_003022843.1), **(D)** Hypothetical protein (WP_003023105.1), **(E)** DUF2147 (WP_003023209.1), **(F)** Hypothetical protein (WP_003017784.1), **(G)** Hypothetical protein (WP_003020066.1), **(H)** Hypothetical protein (WP_003020080.1), and **(I)** Hypothetical protein (WP_003022350.1). Target proteins are highlighted in red font. Nodes are empty for proteins with unknown 3D structures and filled for those with known or predicted structures. Edges represent protein-protein associations and are classified by interaction type; light blue edges indicate interactions from curated databases; purple edges signify experimentally validated interactions; green edges represent predicted interactions based on gene neighborhood; red edges indicate gene fusions; blue edges are derived from gene co-occurrence. Additional sources of interaction include yellow edges for text mining; black edges for co-expression; gray edges for protein homology.

Based on the STRING database, the functions of eight candidate vaccine proteins DUF2147 (WP_003023209.1), hypothetical protein (WP_003023105.1), DUF4124 (WP_003022381.1), carbohydrate-binding protein (WP_227644127.1), hypothetical protein (WP_003022843.1), DUF3281 (WP_003026358.1), PD40 (WP_003021546.1), and hypothetical protein (WP_003029578.1) were not determined. The protein with accession number WP_003023209.1, interacts with the DNA mismatch repair protein (FTT_0486, mutL), several hypothetical proteins (FTT_0066, FTT_1537c, ORF FTT_0485, FTT_0220c, FTT_0045, FTT_1704, and FTT_1349), lipase/acyltransferase (FTT_0023c), and disulfide bond formation protein B (FTT_0107c, *dsbB*). The protein, with accession number WP_003021546.1, interacts with serine hydroxymethyltransferase (SHMT) and deoxyribodipyrimidine photolyase (PhrB). The protein with accession number WP_003023105.1 interacts with an outer membrane lipoprotein (FTT_0198, Blc) and a hypothetical protein (FTT_0200). The proteins with accession numbers WP_003022381.1, and WP_003022843.1 interacted with two hypothetical proteins (**Figure 4**). The string database did not predict the interactions for hypothetical protein (WP_003029578.1), carbohydrate-binding protein (WP_227644127.1), and DUF3281 (WP_003026358.1).

#### 3.1.9 Multi-epitope vaccine construct processing

**Figure 5:**
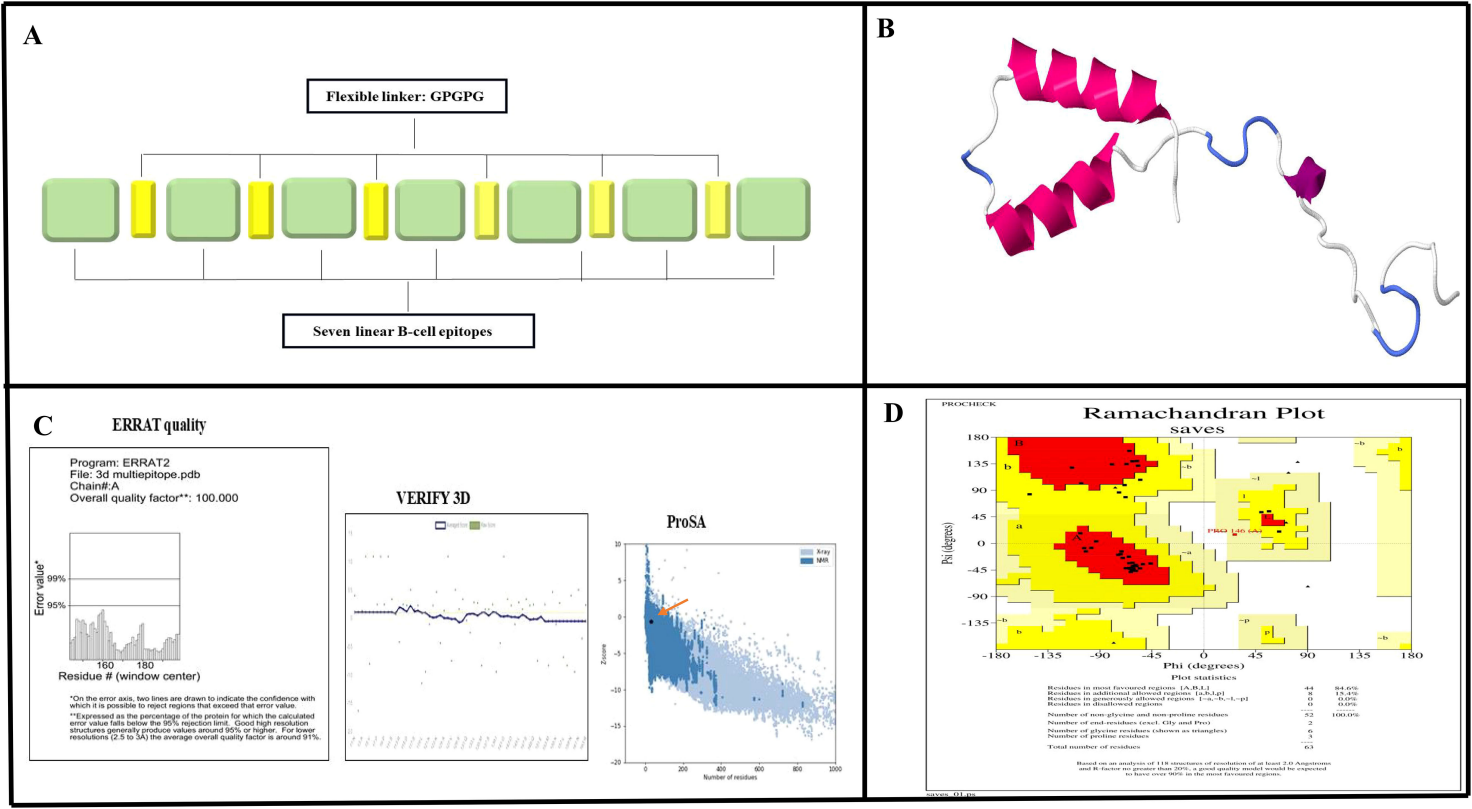
Prediction and validation of the MEV structure. **(A)** Schematic diagram illustrating the arrangement of MEV components, featuring linear B-cell epitopes interconnected by GPGPG flexible linkers. **(B)** Predicted tertiary structure of MEV generated using SWISS-MODEL. **(C)** Validation results for the predicted structure, showing an ERRAT quality score of 100% and successful verification through VERIFY3D analysis. Additionally, a Z-score of -0.61, obtained from Pros A, indicates the model's reliability. **(D)** Ramachandran plot analysis revealed that 84.6% of the residues are in favored regions, 15.4% in allowed regions, and 0% in disallowed regions, further confirming the accuracy of the structure.

**Figure 6:**
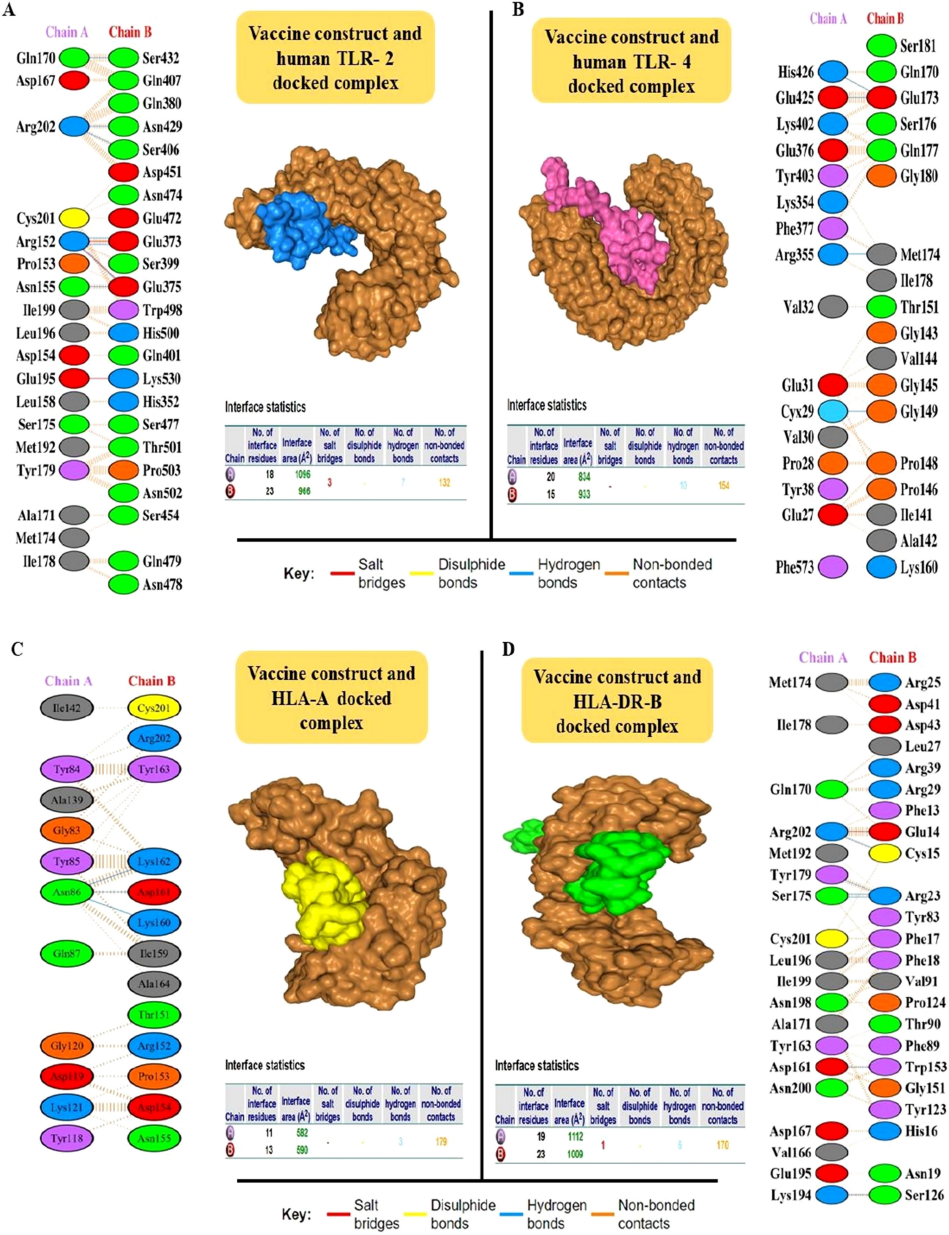
Interaction analysis of the MEV construct with Toll-Like Receptors (TLRs) using HDOCK was visualized using the PDBsum server. The fully docked complexes and residue-by-residue interactions are detailed. **(A)** The MEV-TLR-2 interaction is shown, with the vaccine construct (ligand) in blue and the TLR-2 receptor in brown. The complex formed 10 hydrogen bonds and 154 non-bonded contacts. **(B)** The MEV-TLR-4 interaction is depicted with the vaccine construct (ligand) in pink and TLR-4 in brown. This complex feature seven hydrogen bonds, three salt bridges, and 132 non-bonded contacts. **(C)** The MEV-HLA-A interaction is shown, with the vaccine construct (ligand) in yellow and the HLA-A receptor in brown. The complex formed 3 hydrogen bonds and 179 non-bonded contacts. **(D)** The MEV-HLA-DR-B interaction is shown, with the vaccine construct (ligand) in green and the HLA-DR-B receptor in brown. The complex formed 6 hydrogen bonds, 1 salt bridge and 179 non-bonded contacts.

Of the 12 proteins, seven B-cell epitopes containing T-cell epitopes from four proteins exhibited promising properties for inclusion in the final vaccine construct (**Table 1**). Epitopes were linked using a GPGPG linker. The vaccine sequence was converted into FASTA format and evaluated against various criteria, including antigenicity, non-allergenicity, non-toxicity, and solubility. A schematic representation of the final multi-epitope vaccine peptide is shown in **Figure 5**. The SAVES 6.0 server provided an ERRAT score of 100, and the vaccine demonstrated 84.6% Ramachandran-favored residues with 15.4% in additional allowed regions. The antigenicity score of MEV predicted by the VaxiJen server was 1.061. AlgPred 2.0 and AllerTOP v2.0, were confirmed to be non-allergenic. The selected vaccine candidate exhibited the highest solubility scores, with a value of 0.914. MEV has a low molecular weight (21.27 kDa) and demonstrates extreme thermotolerance, as indicated by its high aliphatic index (61.34). The theoretical pI of MEV was 4.81, and it was recognized as hydrophilic owing to a negative GRAVY score (-0.946). The instability index of the vaccine candidate was 35.9, which categorizes it as a stable polypeptide. The estimated half-life of MEV is 4.4 hours in mammalian reticulocytes *in vitro*, and >10 h in *E. coli in vivo*. The results of protein-protein docking showed that the MEV-TLR-2 complex featured 10 hydrogen bonds and 154 unbound contacts, whereas the MEV-TLR-4 complex exhibited seven hydrogen bonds, three salt bridges, and 132 unbound contacts. The MEV demonstrated nearly equal interactions with TLR-2 (docking score: -235.03 kcal/mol, confidence score: 0.8456, and ligand RMSD: 57.67 Å) and TLR-4 (docking score: -215.94 kcal/mol, confidence score: 0.7890, and ligand RMSD: 42.13 Å). For the HLA interactions, protein-protein docking results showed that the MEV-HLA-A complex featured 3 hydrogen bonds and 179 unbound contacts, with a docking score of -241.44 kcal/mol, a confidence score of 0.8616 and ligand RMSD of 32.93 Å. In comparison, the MEV-HLA-DR-B complex exhibited 6 hydrogen bonds, 1 salt bridge, and 179 unbound contacts, with a docking score of -253.63 kcal/mol, a confidence score of 0.8882, and ligand RMSD of 34.59 Å. The detailed vaccine-receptor interactions are shown in **Figure 6**.

**Table 1:** Seven promising epitopes from four selected *F. tularensis* OMPs were used in the MEV.

Protein (Accession number)	Start	End	length	Epitope	Antigenicity	Ag (Score)	Allergenicity	Conservancy %
Hypothetical protein (WP_003026145.1)	52	72	21	DKGVGEINNSSSVSPNNIAGV	Ag	0.6682	non-allergen	100
Hypothetical protein (WP_003029346.1)	60	86	27	NHNAKLQANDTIKYEIKQKQNIPWKSL	Ag	0.8526	non-allergen	100
OmpA family protein (WP_003020808.1)	23	75	53	STRPDNSDLIKDKYAGVDSSQALEMSSQIYGSDKLSSDQ VEQMKKELMNINCR	Ag	0.6334	non-allergen	100
PD40 (WP_003021546.1)	20	50	31	ADDLNAKIVNESVTKYSNNVETDADTNTNSP	Ag	1.2715	non-allergen	100
	230	245	16	YNINSKDAATAIEFND	Ag	1.0516	non-allergen	100
	255	263	9	IKSLQGSDT	Ag	1.0523	non-allergen	100
	402	416	15	IHYQHNDDNKIDHLD	Ag	0.7518	non-allergen	100

Start: The starting amino acid position of the epitope within the protein sequence; End: The ending amino acid position of the epitope within the protein sequence, Length: The number of amino acids in the epitope sequence; Antigenicity: Indicates if the epitope is recognized as an antigen; Antigenicity Score: A numerical score reflecting the strength of the epitope’s antigenic potential, with higher scores indicating greater antigenicity; Allergenicity: Indicates whether the epitope is considered an non-allergen; Conservancy (%): The percentage of conservation of the epitope across different species or strains.

#### 3.1.10 *In silico* cloning of the design

**Figure 7:**
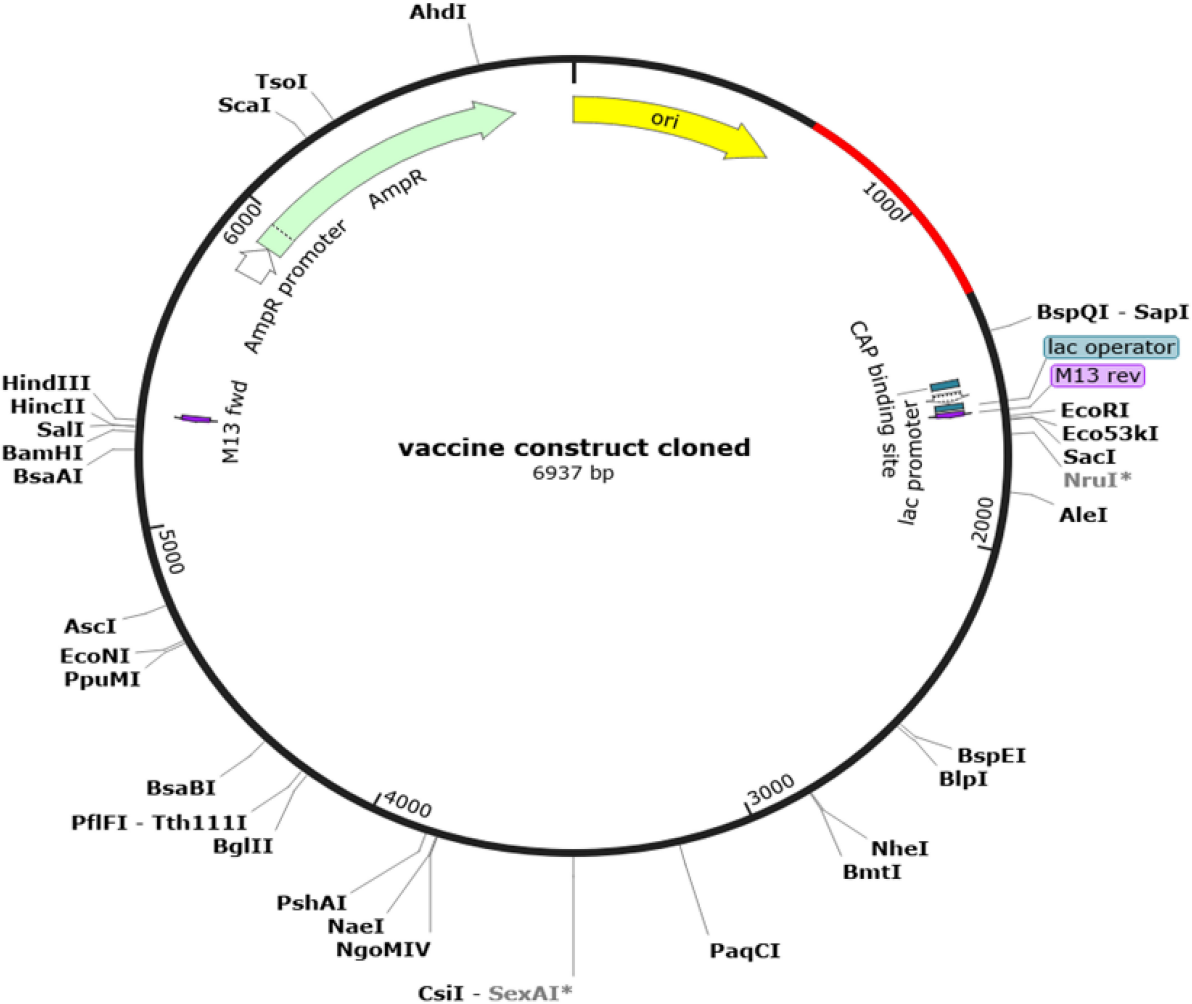
Results of *in Silico* restriction cloning. Computational restriction cloning of the reverse-translated MEV candidate fragment into the ATOH1_Puc18 expression plasmid vector was performed using SnapGene 7.2. The black ring represents the vector backbone, while the red arrow indicates the reverse-translated MEV fragment.

To optimize processivity and expression in *E. coli*, the MEV sequence was subjected to rigorous reverse translation using the *E. coli* K12 codon table. The Codon Adaptation Index (CAI) was calculated as 0.95. This index quantifies how well codon usage in a sequence matches the codon usage bias of the host organism, with a value of 1.0, indicating perfect adaptation. The sequence also exhibited a GC content of 52.05%, which falls within the favorable range (30-70%) for expression in *E. coli*. Subsequently, using the SnapGene software, the vaccine sequence was inserted into a highly efficient ATOH1_Puc18 expression plasmid vector (**Figure 7**).

#### 3.1.11 Immune simulation of predicted vaccine construct

**Figure 8:**
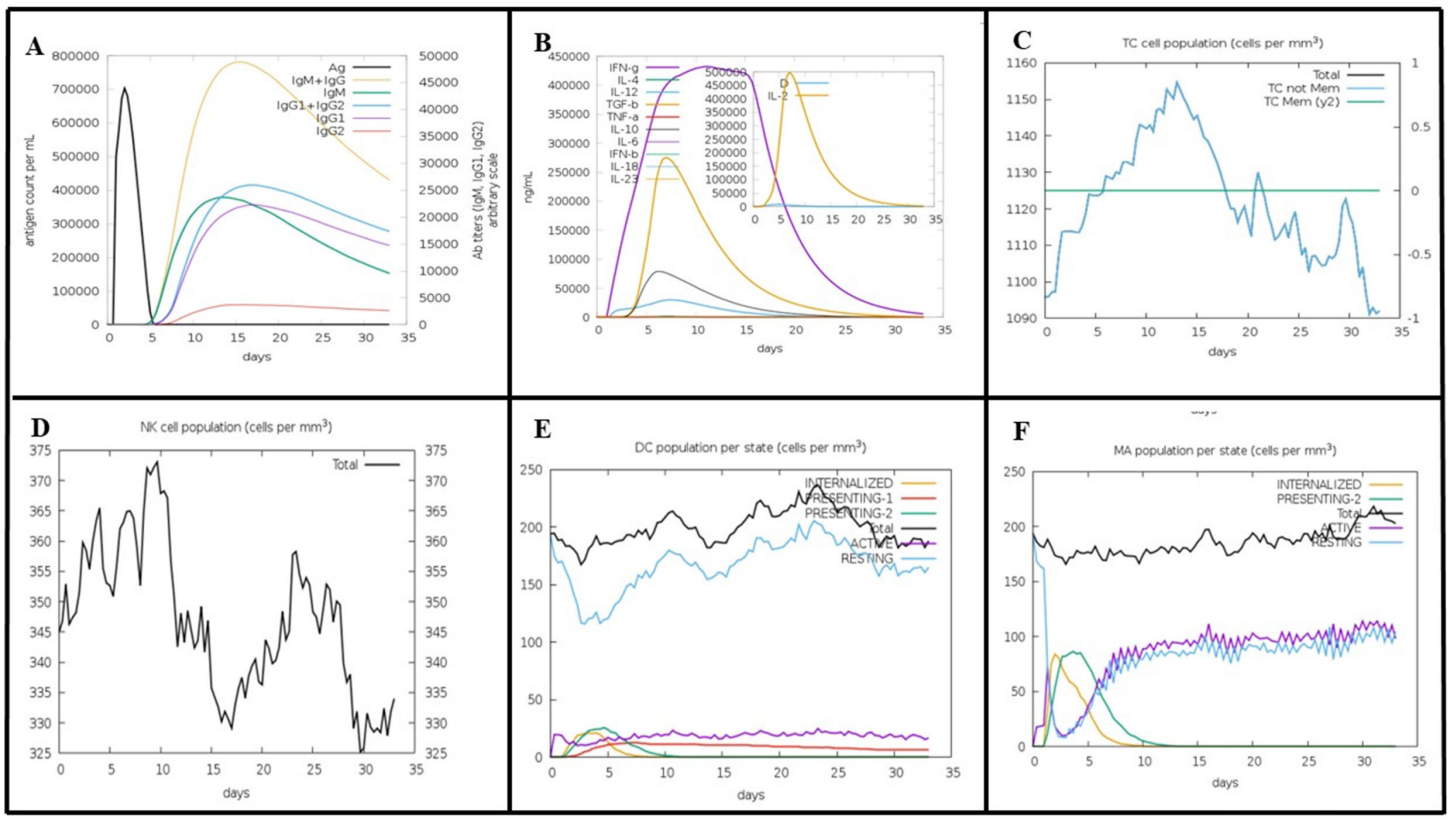
Predicted Immune Response to MEV Vaccine Construct Using the C-ImmSim Tool. **(A)** MEV elicits a rapid immune response with an initial IgM peak, followed by sustained levels of IgG (IgG1 + IgG2), indicating prolonged immunity. **(B)** Early cytokine response, dominated by IFN-γ, TGF-β, and IL-2, peaks on days 5-6, with IL-12 and IL-10 contributing to immune regulation on days 6-7. **(C)** T-cells (CD4 and CD8) peak around days 10-15, then gradually decline by day 32. **(D)** NK cells reach peak levels between days 7-10, followed by a gradual decrease over 35 days. **(E)** The initial decrease in dendritic cells reflects the early activation in response to MEV. Intern, internalized antigen; Pres II, presenting on MHC II; Dup, mitotic cycle; anergic, anergic; resting, inactive.

The vaccine construct peaked rapidly after injection, and then declined, indicating a rapid initial immune response. IgM was the first antibody to respond to MEV and peaks shortly after MEV exposure. Subsequently, IgG antibodies (IgG1 + IgG2) were raised, and the elevation of these two antibodies was critical for long-term immunity. The combined IgM and IgG response showed a broad peak, indicating a sustained antibody presence to combat the antigen (**Figure 8A**). The initial immune response appears to be driven by IFN-γ, TGF-β, and IL-2, as their levels peaked at around day 5-6. This was followed by a peak in IL-12 and IL-10 levels on day 6-7, suggesting their involvement in sustaining and regulating immune response (**Figure 8B**). TC cells (CD4 T-cells and CD8 T-cells) peaked around day 10-15, and after 32 days, TC cells populations exhibited decline in concentration (**Figure 8C**). The population of natural killer (NK) cells peaked around days 7-10 and declines over 35 days (**Figure 8D**). The initial drop in the total and resting dendritic cell (DC) populations may be attributed to an early response to external injection of the vaccine construct (**Figure 8E**). This decline was followed by recovery, indicating an adaptive mechanism that restores the cell populations to a stable state. These dynamics highlight the effective engagement of dendritic cells in processing and presenting vaccine antigens, which are essential for a successful immunization response. These results suggest that the vaccine candidate successfully activated the immune system while maintaining the overall stability of the dendritic cell populations. Following the injection of the vaccine candidate, the macrophage populations exhibited dynamic changes, indicative of an effective immune response (**Figure 8F**).

#### 3.1.12 Molecular dynamic simulation

**Figure 9:**
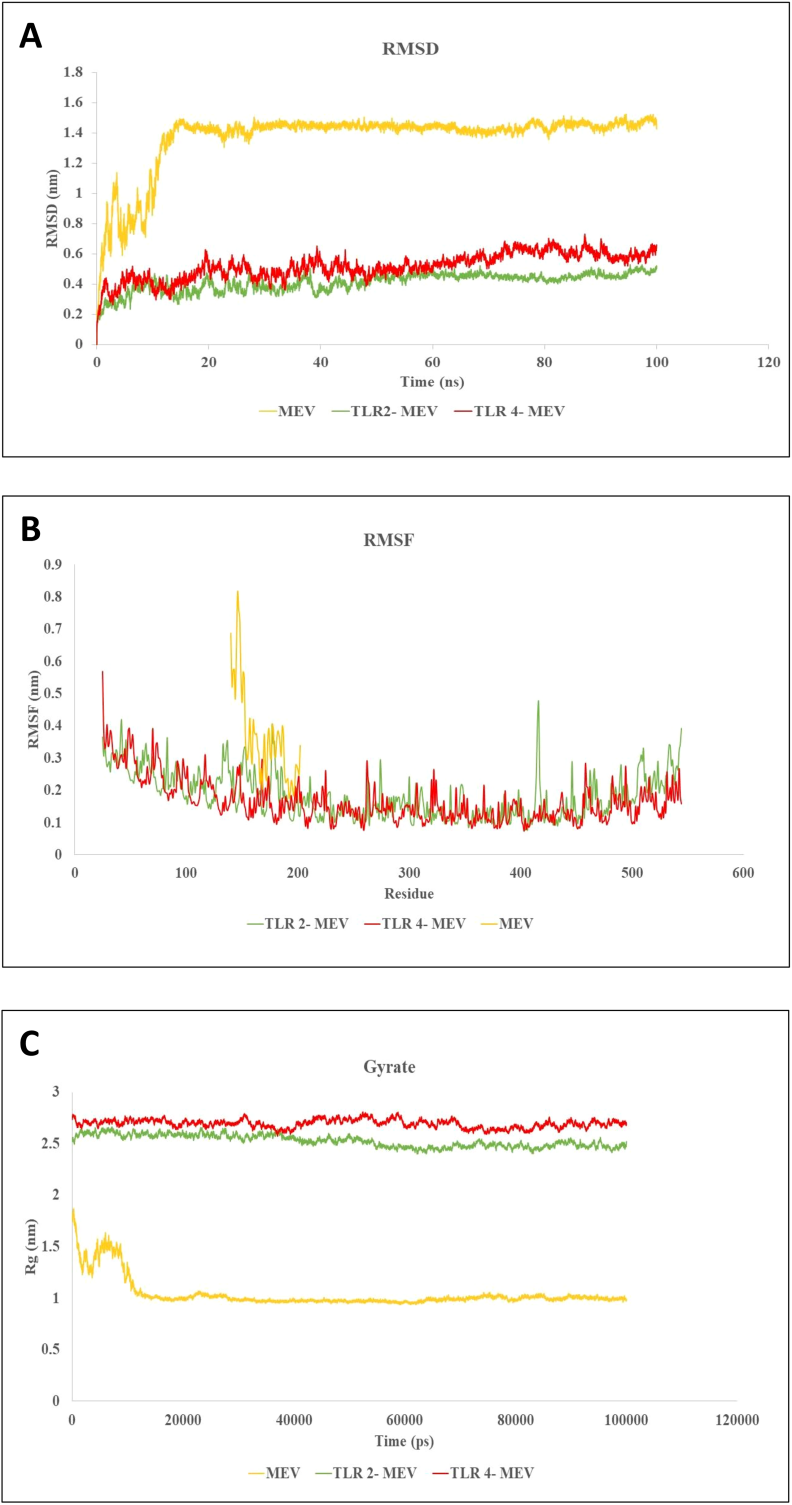
Molecular dynamics simulations of MEV and its Complexes with TLR-2 and TLR-4. **(A)** RMSD plot showing the stability of the MEV molecule and the MEV_TLR-2 and MEV_TLR-4 complexes throughout the simulation, with equilibrium RMSD values indicating stable interactions. **(B)** RMSF plot illustrating residue flexibility, where higher RMSF values represent more flexible regions and lower values denote rigidity across the MEV, MEV_TLR-2, and MEV_TLR-4 systems. **(C)** Radius of gyration (Rg) plot reflecting the shape and compactness of each system over time. Stable Rg values indicate consistent molecular shapes, while fluctuations indicate conformational changes. These simulations offer insights into the stability, flexibility, and structural evolution of MEV molecules and their interactions with TLR receptors under cytosolic conditions.

MD simulations have proven invaluable in guiding experimental validation by analyzing conformational changes, stability variations, and the overall evolution of complex systems under cytosol-like conditions. These simulations allowed for the assessment of parameters such as the RMSD, root mean square fluctuation (RMSF), and radius of gyration. RMSD is a critical metric for evaluating the stability of receptor-ligand complexes. In our MD simulation study, the RMSD graph revealed the MEV, MEV_TLR-2 complex, and MEV_TLR-4 complex exhibited stable interactions, as shown in **Figure 9A**. Furthermore, we quantified the RMSF of the complexes to assess their flexibility across amino acid residues. **Figure 9B** illustrates that the RMSF values for the MEV, MEV_TLR-2 complex, and MEV_TLR-4 complex were similar, indicating similar flexibility. Additionally, we analyzed the radius of gyration (Rg) to evaluate the mobility and overall flexibility of the complexes. The gyration graph shown in **Figure 9C** corroborates the RMSD findings.

### 3.2 Identification of novel drug targets

#### 3.2.1 Detection of cytoplasmic protein sequences: proteins with no similarities to the host

All proteins from the reference strain (1921 proteins) were analyzed using PSORTb and CELLO. A total of 918 cytoplasmic proteins were identified. Using PSI-BLAST, 520 proteins showed no similarity to human proteins, while 398 proteins were excluded due to their similarity with the human proteome. None of the proteins exhibited similarities with human mitochondrial proteins.

#### 3.2.2 Identification of unique metabolic pathway proteins

Following BLAST analysis using the KAAS database, we identified 282 of the 520 non-homologous proteins that were involved in metabolic pathways common to humans and were consequently excluded from further analysis. The remaining 238 non-homologous proteins were retained for subsequent analyses.

#### 3.2.3 Selecting essential protein sequences

Antibacterial agents are often designed to target and inhibit critical gene products, making essential proteins particularly effective therapeutic targets (60). A total of 37 proteins were identified as significant hits using a dataset of essential bacterial proteins from the DEG 15.2 database. These proteins are essential for the survival of *F. tularensis*, underscoring their potential as targets for therapeutic intervention (61). Targeting the unique genetic components specific to microbes can be particularly advantageous for developing species-specific treatments.

#### 3.2.4 Identify novel drug targets

Among the 37 F*. tularensis* proteins identified as significant hits from the DEG 15.2 database, four proteins showed similarities to targets of FDA-approved or experimental drugs listed in the DrugBank database and were therefore excluded from further analysis. The remaining 33 proteins were selected for further investigation.

#### 3.2.5 Determining host microbiome non-similar proteins

In the final analysis, 10 pathogen proteins were identified using microbiome BLAST, which showed no similarity to the human microbial flora (**Table 2**). Therefore, these proteins have been proposed as exclusive drug targets for combating various *F. tularensis* strains.

**Table 2:** The shortlist of 10 novel drug targets against *F. tularensis*.

No.	Accession number)	EggNOG and CCD	Gut microbiota BLAST^*^
1	WP_003017413.1	Asp-tRNA (Asn)/Glu-tRNA (Gln) amidotransferase subunit GatC	–
2	WP_003023081.1	30S ribosomal protein S16	–
3	WP_003017784.1	Hypothetical protein	–
4	WP_003014157.1	Haloacid dehalogenase	–
5	WP_003020066.1	Hypothetical protein	–
6	WP_003020080.1	Hypothetical protein	–
7	WP_003022220.1	uroporphyrinogen-III synthase	–
8	WP_042522581.1	NAD(P)-binding protein	–
9	WP_003022345.1	class I SAM-dependent methyltransferase	–
10	WP_003022350.1	Hypothetical protein	–

*: Minus symbol indicates no significant similarity was found with gut microbiota proteins.

#### 3.2.6 Protein-protein interactions

Based on CDD and EggNOG analyses, the functions of six drug target proteins were detected, whereas four hypothetical proteins (WP_003017784.1, WP_003020066.1, WP_003020080.1, and WP_003022350.1) lacked identifiable functions (**Table 2**). Based on the STRING database results, hypothetical proteins (WP_003017784.1) interacts closely with the cytochrome b561 family protein (FTT_0219c), lipase/acyltransferase proteins (FTT_0023c), Type IV pili lipoprotein (FTT_1057c), conserved hypothetical protein (FTT_1537c), LicB-like transmembrane protein (FTT_0157c), conserved membrane protein similar to Q9X885 (FTT_0181c), a conserved hypothetical protein similar to AAP58972.1 (Q7X3I5) from *F. novicida* (FTT_1704), and two hypothetical proteins (FTT_0485, FTT_0066). Hypothetical protein (WP_003020066.1) shows an expression correlation with stringent starvation protein A (FTT_0458) and is similar to the macrophage growth locus A protein from *F. novicida* (FTT_1275). Hypothetical protein (WP_003020080.1) interacts with a hypothetical protein (FTT_0392c), methionine aminopeptidase (FTT_0393), 5'-methylthioadenosine/S-adenosylhomocysteine nucleosidase (FTT_0397), and DNA topoisomerase IV subunit A (FTT_0396). Hypothetical protein (WP_003022350.1) interacts with Tryptophanyl-tRNA synthetase (TrpS), antiporter protein (FTT_1490), Dephospho-CoA kinase (CoaE), Adenylylsulfate kinase (MsrA1), and Choloylglycine hydrolase (Cbs) (**Figure 4**).

## 4 Discussion

Single-antigen vaccines against tularemia are insufficient to provide comprehensive protection, highlighting the necessity for multi-antigen strategies (62–65). Multi-antigen vaccines that incorporate various proteins typically offer improved efficacy by activating broader immune responses (66–68). For example, a tri-antigen vaccine (DnaK, OmpA, and Tul4) generated appropriate antibody responses, but showed low protective efficacy (69). This indicates that while antibodies play a crucial role, effective protection also relies on strong T-cell responses and optimal combinations of antigens. A deeper understanding of these factors is essential for designing more effective vaccines.

In this study, we designed an MEV using immunodominant epitopes from *F. tularensis* OMPs to stimulate both B-cell and T-cell responses to protect against tularemia (70). It has been shown that OMP immunization provides the highest level of protection against tularemia in animal models (66). The membrane components of *F. tularensis* have demonstrated protective efficacy in both prophylactic and post-exposure therapeutic models of tularemia (66, 71, 72). Additionally, a study using a reverse vaccinology approach identified surface proteins as target immunogens for antibody-based immunotherapies against tularemia (73). Reverse vaccinology is a powerful technique for identifying immunogenic and surface-exposed proteins (74, 75), thus enabling the discovery of novel vaccine candidates that traditional methods may miss. An example of such success is the 4CMenB vaccine (Bexsero), the first approved vaccine against *Neisseria meningitidis* serogroup B (MenB) (76, 77). These examples highlight the importance of including a mix of antigens to achieve a protective immune response (78).

Our proposed MEV includes seven immunogenic epitopes from four OMPs, selected for their high antigenicity, solubility, thermostability, and optimal half-life. *In silico* analysis indicated effective expression and purification in *E. coli*, suggesting a cost-effective manufacturing process (79). The docking results indicated that the MEV construct strongly interacted with both TLR-2 and TLR-4, as well as HLA-A and HLA-DR-B, key receptors for initiating immune responses. The stable binding observed with both Class I and Class II MHC molecules is crucial for eliciting T cell-mediated adaptive immunity. These findings suggest that MEV has the potential to stimulate both the innate and adaptive immune pathways, thereby providing broad protection. This design yielded a stable and reproducible MEV, supported by docking and MD simulations, as well as the known roles of TLR2 (80), suggesting that the MEV vaccine construct has the potential to trigger a robust and multifaceted immune response and could be safe for human use. The simulations indicated a strong immune response to *F. tularensis* infection, suggesting a long-lasting immunity. The involvement of immune cells, such as macrophages, T-cells, and NK cells, along with signaling molecules, such as IL-2, IFN-γ, and IL-12, indicates a comprehensive immune response. These results align with those of studies highlighting the roles of IL-2, TNF-α, IFN-γ, and IL-12 in the management of *F. tularensis* infection (81–83). Additionally, the presence of antibodies (IgM and IgG2) suggests mechanisms such as macrophage phagocytosis and complement activation, further supporting MEV's multi-faceted approach (84). However, while the simulation highlighted increased immune cell populations, the absence of activation markers raises concerns regarding the accuracy of predicting functional immune engagement and vaccine efficacy. The recruitment of T-cells or macrophages does not ensure their active participation in the immune response. Activation markers such as CD80/CD86 and MHC are crucial for verifying that antigen-presenting cells (APCs) effectively communicate with T-cells, priming them for action. Without these markers, the immune response may remain suboptimal, potentially compromising the overall protective efficacy of the vaccine (85). This underscores the importance of further validation through both *in vitro* and *in vivo* studies to confirm the functional activation of immune cells.

To overcome the limitations of core proteins and achieve broad-spectrum protection, we employed a two-pronged strategy. First, we targeted vaccine candidates with high prevalence (> 90%) across a wide range of pathogenic *F. tularensis* strains, which were identified using a highly virulent strain as a reference. Second, we prioritized epitopes that exhibited complete sequence conservation (100 %). This comprehensive approach has the potential to develop a vaccine capable of providing robust protection against a broad spectrum of tularemia-causing *F. tularensis* strains. Supporting this strategy, a study by Subrat Kumar Swain et al. demonstrated that the strategic combination of B-cell and T-cell epitopes effectively induces a strong and durable immune response, thereby establishing both cellular and humoral immunity (86). This dual-epitope strategy enhanced the ability of the vaccine to elicit a well-rounded and long-lasting immune response against *F. tularensis* strains.

Abbas Khan et al. employed structural proteomics to design a multi-epitope vaccine for tularemia, using *F. novicida*, a less virulent strain (87). In contrast, our approach focused on *F. tularensis* type A, isolated from a patient with tularemia, ensuring greater relevance for vaccine development. This differs, from the findings of Khan et al, who identified only four potential targets in *F. novicida* (87). This discrepancy highlights the potential strain-specific variations in immunogenic targets. Of the 12 proteins identified in our study, only four contained suitable epitopes for MEV design. This demonstrates that a protein may be antigenic and exhibit other essential vaccine candidate properties, yet still lack suitable epitopes for eliciting protective immunity. For instance, FopA was identified as a vaccine candidate in our study but did not provide any suitable epitopes. Similarly, previous studies have shown that FopA does not elicit protective immunity against tularemia in animal models (88).

This study identified ten promising drug development targets that are crucial for the survival and metabolism of *F. tularensis*, thus making them ideal candidates for novel treatments. The targets included Asp-tRNA (Asn)/Glu-tRNA (Gln) amidotransferase subunit GatC (WP_003017413.1), NAD(P)-binding protein (WP_042522581.1), 30S ribosomal protein S16 (WP_003023081.1), Class I SAM-dependent methyltransferase (WP_003022345.1), haloacid dehalogenase (WP_003014157.1), and uroporphyrinogen-III synthase (WP_003022220.1). Four hypothetical proteins (WP_003017784.1, WP_003020080.1, WP_003020066.1, WP_003022350.1). These targets offer significant potential for developing new therapeutic interventions. Among these, GatC (WP_003017413.1), an amidotransferase subunit, is essential for bacteria to thrive in host cells. Inhibition of GatC can effectively prevent infection by halting bacterial proliferation (89). The NAD(P)-binding protein (WP_042522581.1) is another promising target. This vital coenzyme is involved in numerous biochemical processes, with approximately 5.4% of the proteins in the UniProtKB/Swiss-Prot database being annotated as NAD(P)-binding. These proteins, such as ADP-ribosylating toxins and poly-ADP-ribose polymerases, are recognized as therapeutic targets. Inhibition of NAD(P)-binding proteins can disrupt crucial redox and non-redox reactions within a pathogen, thereby crippling its metabolic functions (90). 30S ribosomal protein S16 (WP_003023081.1) plays a critical role in protein synthesis. Targeting bacterial ribosomes has been a successful antibiotic strategy, exemplified by drugs such as linezolid, tetracycline, and chloramphenicol (91–93). Class I SAM-dependent methyltransferase (WP_003022345.1) is also a promising target because of its vital role in cellular processes, such as gene expression, protein function, and cell signaling. Inhibition of these enzymes could disrupt these critical functions, hindering the ability of the bacterium to operate (94). Haloacid dehalogenase (WP_003014157.1), a HAD-like phosphatase, performs various cellular functions including primary and secondary metabolism, enzyme activity or protein assembly regulation, cell housekeeping, and nutrient uptake. Targeting this enzyme can disrupt the essential processes (95). Uroporphyrinogen III synthase (WP_003022220.1) is a cytosolic enzyme involved in heme biosynthesis, catalyzing the formation of uroporphyrinogen III from hydroxymethylbilane. Targeting this enzyme can interfere with the heme biosynthesis pathway in bacteria (96). In the present study, we identified four hypothetical proteins with unknown functions as potential vaccine targets. Understanding the roles of these proteins could open new avenues for treatment, underscoring the importance of further research to uncover novel therapeutic possibilities.

## 5 Conclusion

Tularemia demands the development of novel vaccine candidates, identification of new drug targets, and the adoption of modern strategies for effective management. In this study, we identified ten proteins involved in key cellular processes and pathways as potential immunogenic targets, including GatC, NAD(P)-binding protein, 30S ribosomal protein S16, Class I SAM-dependent methyltransferase, haloacid dehalogenase, uroporphyrinogen-III synthase, and four hypothetical proteins, which warrant further investigation. To address the challenges posed by *F. tularensis* and improved prophylactic measures, we designed an MEV. Rational design and proven safety of MEVs have accelerated the development of more stable, efficient, and broad-spectrum vaccine candidates that target a range of pathogens and cancers. This rapidly advancing field of immunoinformatics offers great potential for reducing time and resource expenditure, speeding up vaccine discovery, and making it an area ripe for further exploration. Our proposed MEV was predicted to be non-allergenic and to exhibit favorable physicochemical properties. Molecular docking studies combined with molecular dynamics simulations demonstrated the ability of the vaccine to form strong and stable interactions with immune receptors. Immune simulations further suggested the potential of this vaccine to elicit robust immune responses. While the construct shows promise as a safe and effective solution for combating tularemia, comprehensive *in vitro* and *in vivo* studies are essential to assess its efficacy under native conditions. The promising results of the computational analyses will be experimentally evaluated to confirm by the authors soon.

## Data Availability

The genome of Francisella tularensis is accessible at https://www.ncbi.nlm.nih.gov/datasets/genome/?taxon=263. The article files will be made available upon publication, and additional materials can be found in the Supplementary Information Files.
