# Supplementary material for: Development of a multi-epitope vaccine from outer membrane proteins and identification of novel drug targets against Francisella tularensis: an In Silico approach
Source: Front Immunol. 2025 Apr 3;16:1479862. doi: 10.3389/fimmu.2025.1479862 (PMC12003292; doi:10.3389/fimmu.2025.1479862)
Supplement: Supplementary file 1 [file DataSheet1.docx]

**Supplementary Data 1.** Gut microbiota strains

Actinomyces odontolyticus ATCC 17982 (taxid: 411466)

Akkermansia muciniphila ATCC BAA-835 (taxid: 349741)

Alistipes putredinis DSM 17216 (taxid: 445970)

Anaerofustis stercorihominis DSM 17244 (taxid: 445971)

Anaerostipes caccae DSM 14662 (taxid: 105841)

Anaerotruncus colihominis DSM 17241 (taxid: 445972)

Pseudoflavonifractor capillosus ATCC 29799 (taxid: 411467)

Bacteroides cellulosilyticus DSM 14838 (taxid: 537012)

Bacteroides coprocola DSM 17136 (taxid: 470145)

Bacteroides dorei DSM 17855 (taxid: 483217)

Bacteroides eggerthii DSM 20697 (taxid:483216)

Bacteroides finegoldii DSM 17565 (taxid: 483215)

Bacteroides intestinalis DSM 17393 (taxid: 471870)

Bacteroides ovatus ATCC 8483 (taxid: 411476)

Bacteroides pectinophilus ATCC 43243 (taxid: 483218)

Bacteroides plebeius DSM 17135 (taxid: 484018)

Bacteroides stercoris ATCC 43183 (taxid: 449673)

Bacteroides uniformis ATCC 8492 (taxid: 411479)

Bifidobacterium adolescentis ATCC 15703 (taxid: 367928)

Bifidobacterium adolescentis L2-32 (taxid: 411481)

Bifidobacterium angulatum DSM 20098 (taxid:518635)

Bifidobacterium bifidum DSM 20456 (taxid:500634)

Bifidobacterium breve DSM 20213 (taxid: 518634)

Bifidobacterium dentium ATCC 27678 (taxid:473819)

Bifidobacterium longum DJO10A (taxid:205913)

Bifidobacterium longum NCC2705 (taxid:206672)

Bifidobacterium longum subsp infantis str ATCC 15697 (taxid:391904)

Borrelia burgdorferi CA-11.2A (taxid:498739)

Butyrivibrio crossotus DSM 2876 (taxid: 511680)

Catenibacterium mitsuokai DSM 15897 (taxid:451640)

Clostridium asparagiforme DSM 15981 (taxid: 518636)

Clostridium bartlettii DSM 16795 (taxid: 445973)

Clostridium bolteae ATCC BAA-613 (taxid : 411902)

Clostridium hiranonis DSM 13275 (taxid:500633)

Clostridium leptum DSM 753 (taxid:428125)

Clostridium methylpentosum DSM 5476 (taxid:537013)

Clostridium nexile DSM 1787 (taxid: 500632)

Clostridium ramosum DSM 1402 (taxid: 445974)

Clostridium scindens ATCC 35704 (taxid: 411468)

Clostridium sp L2-50 (taxid:411489)

Clostridium sp M62/1 (taxid:411486)

Clostridium sp SS2/1 (taxid:411484)

Clostridium spiroforme DSM 1552 (taxid:428126)

Clostridium sporogenes ATCC 15579 (taxid:471871)

Clostridium symbiosum ATCC 14940 (taxid:411472)

Collinsella aerofaciens ATCC 25986 (taxid:411903)

Collinsella intestinalis DSM 13280 (taxid: 521003)

Collinsella stercoris DSM 13279 (taxid: 445975)

Coprococcus comes ATCC 27758 (taxid : 470146)

Coprococcus eutactus ATCC 27759 (taxid:411474)

Dorea formicigenerans ATCC 27755 (taxid:411461)

Dorea longicatena DSM 13814 (taxid:411462)

Eggerthella lenta DSM 2243 (taxid:479437)

Enterobacter cancerogenus ATCC 35316 (taxid: 500639)

Eubacterium dolichum DSM 3991 (taxid: 428127)

Eubacterium hallii DSM 3353 (taxid:411469)

Eubacterium siraeum DSM 15702 (taxid:428128)

Eubacterium ventriosum ATCC 27560 (taxid:411463)

Faecalibacterium prausnitzii A2-165 (taxid:411483)

Faecalibacterium prausnitzii M21/2 (taxid:411485)

Lactobacillus salivarius UCC118 (taxid: 362948)

Methanobrevibacter smithii ATCC 35061 (taxid:420247)

Methanobrevibacter smithii DSM 11975 (taxid: 521001)

Methanobrevibacter smithii DSM 2374 (taxid:521002)

Methanobrevibacter smithii DSM 2375 (taxid:483214)

Mitsuokella multacida DSM 20544 (taxid:500635)

Parabacteroides johnsonii (taxid:387661)

Parabacteroides merdae ATCC 43184 (taxid:411477)

Parvimonas micra ATCC 33270 (taxid:411465)

Photorhabdus luminescens subsp laumondii TTO1 (taxid:243265)

Prevotella copri DSM 18205 (taxid:537011)

Providencia alcalifaciens DSM 30120 (taxid:520999)

Providencia rettgeri DSM 1131 (taxid: 521000)

Providencia rustigianii DSM 4541 (taxid:500637)

Roseburia faecis M72/1 (taxid:301302)

Roseburia intestinalis L1-82 (taxid: 536231)

Ruminococcus gnavus ATCC 29149 (taxid: 411470)

Ruminococcus lactaris ATCC 29176 (taxid:471875)

Ruminococcus obeum ATCC 29174 (taxid: 411459)

Ruminococcus torques ATCC 27756 (taxid:411460)

Streptococcus infantarius subsp infantarius ATCC BAA-102 (taxid:471872)

Subdoligranulum variabile DSM 15176 (taxid:411471)

Victivallis vadensis ATCC BAA-548 (taxid:172901)
