# Supplementary material for: Development of a multi-epitope vaccine from outer membrane proteins and identification of novel drug targets against Francisella tularensis: an In Silico approach
Source: Front Immunol. 2025 Apr 3;16:1479862. doi: 10.3389/fimmu.2025.1479862 (PMC12003292; doi:10.3389/fimmu.2025.1479862)
Supplement: Supplementary file 2 [file DataSheet2.docx]

**Suplementary Data 2. Validation results for the predicted structures of all 12 proteins, assessed through ERRAT quality verification, VERIFY 3D analysis, ProSA evaluation, and Ramachandran plot analysis.**

Table.1 Validation results for the predicted structures of all 12 proteins, assessed through ProSA evaluation

| **OmpA family protein (WP_003020808.1)** | | **PD40 (WP_003021546.1)** |
| --- | --- | --- |
| **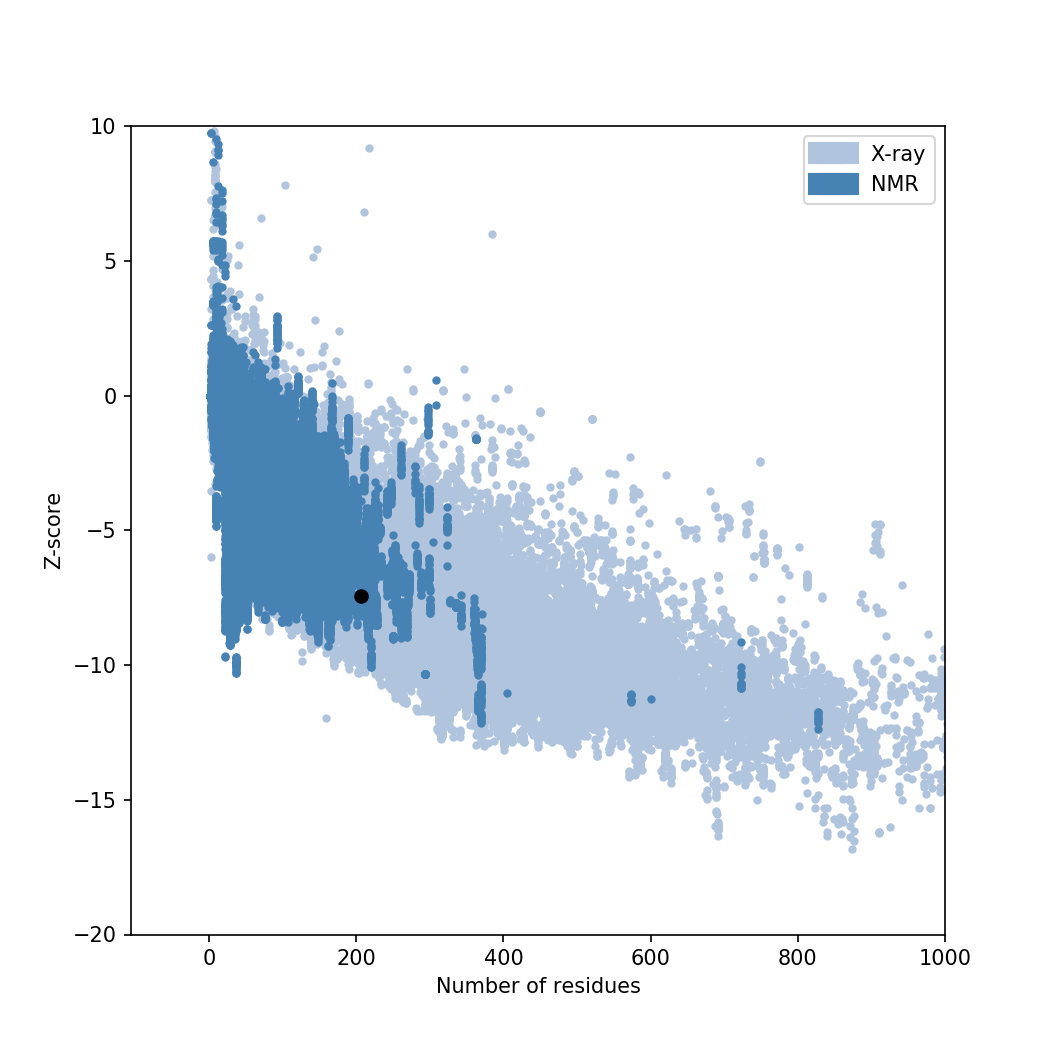** | **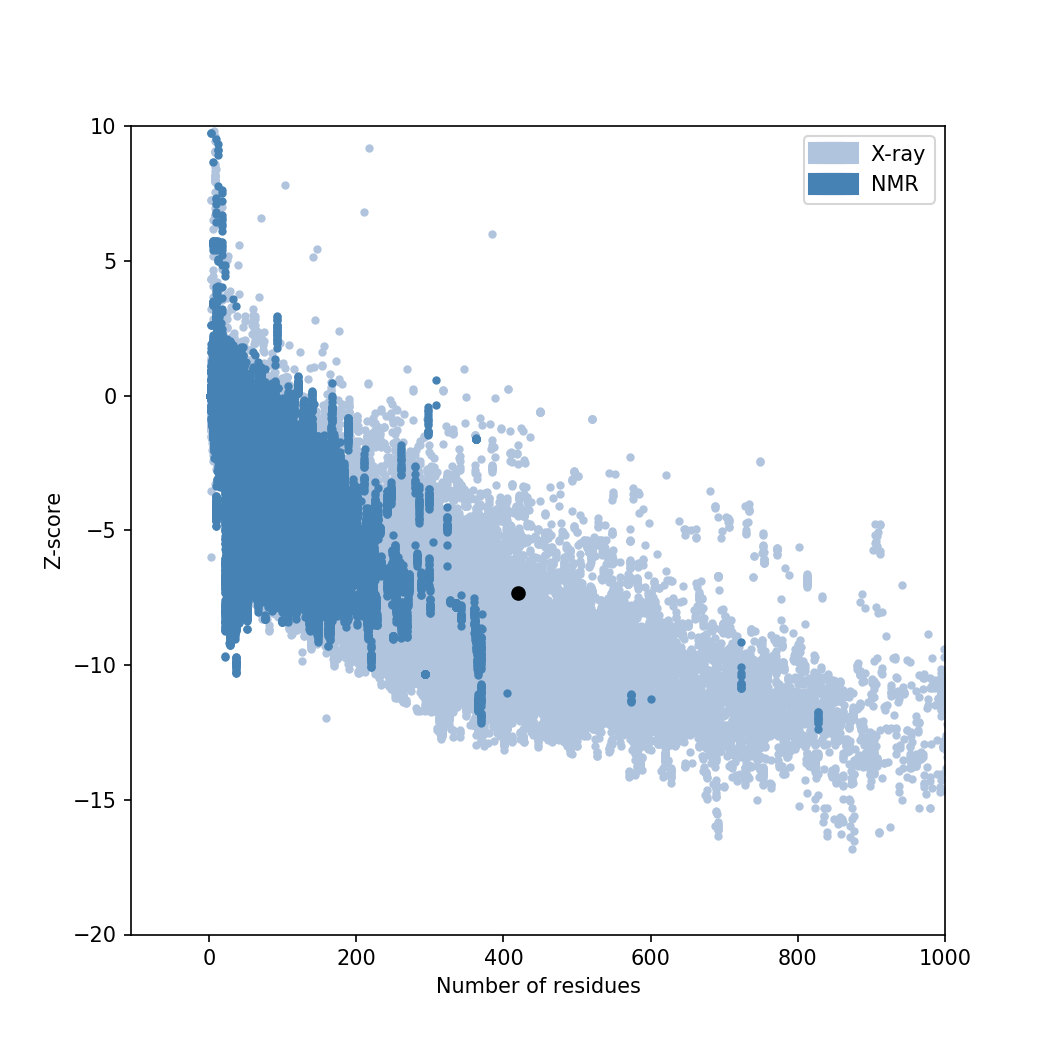** |  |
| **DUF4124 (WP_003022381.1)** | **Hypothetical protein (WP_003022843.1)** |  |
| **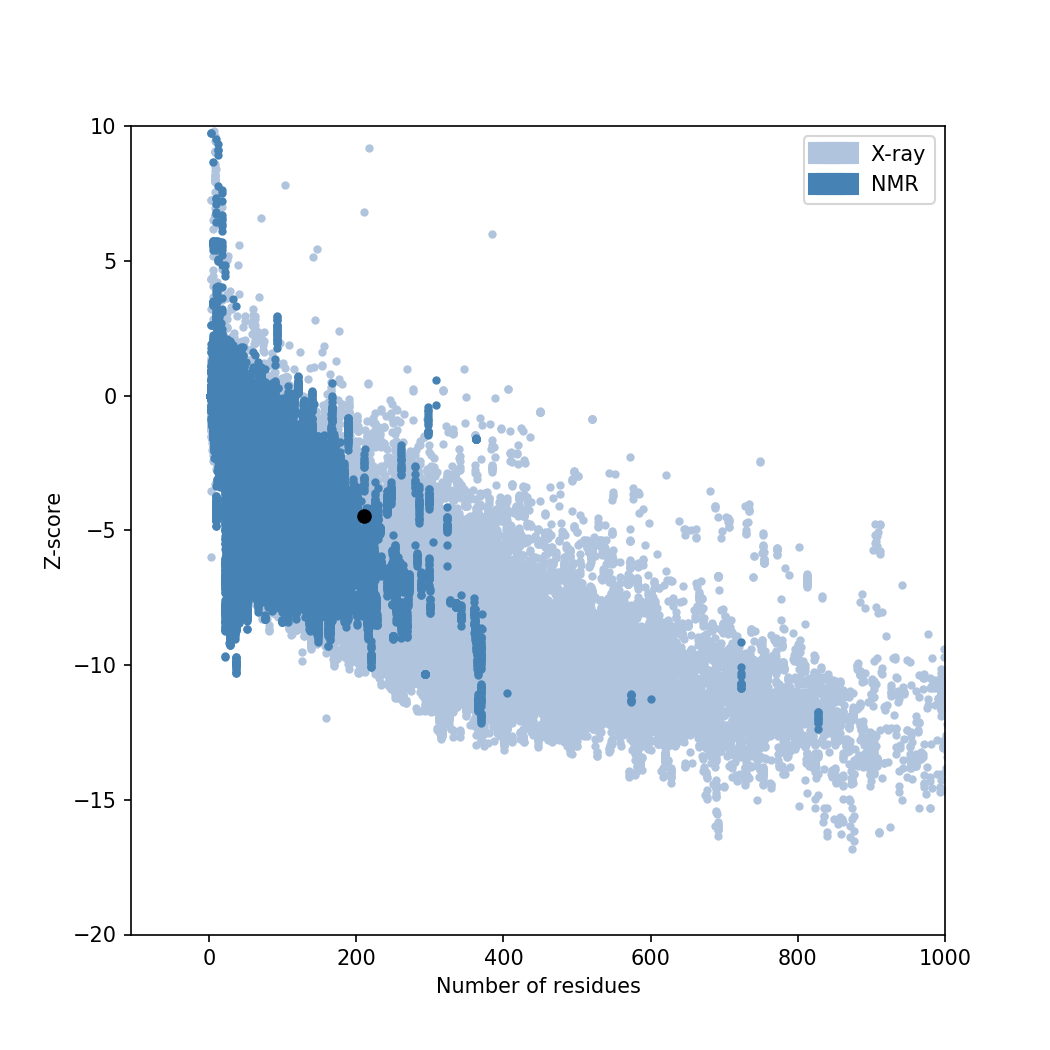** | **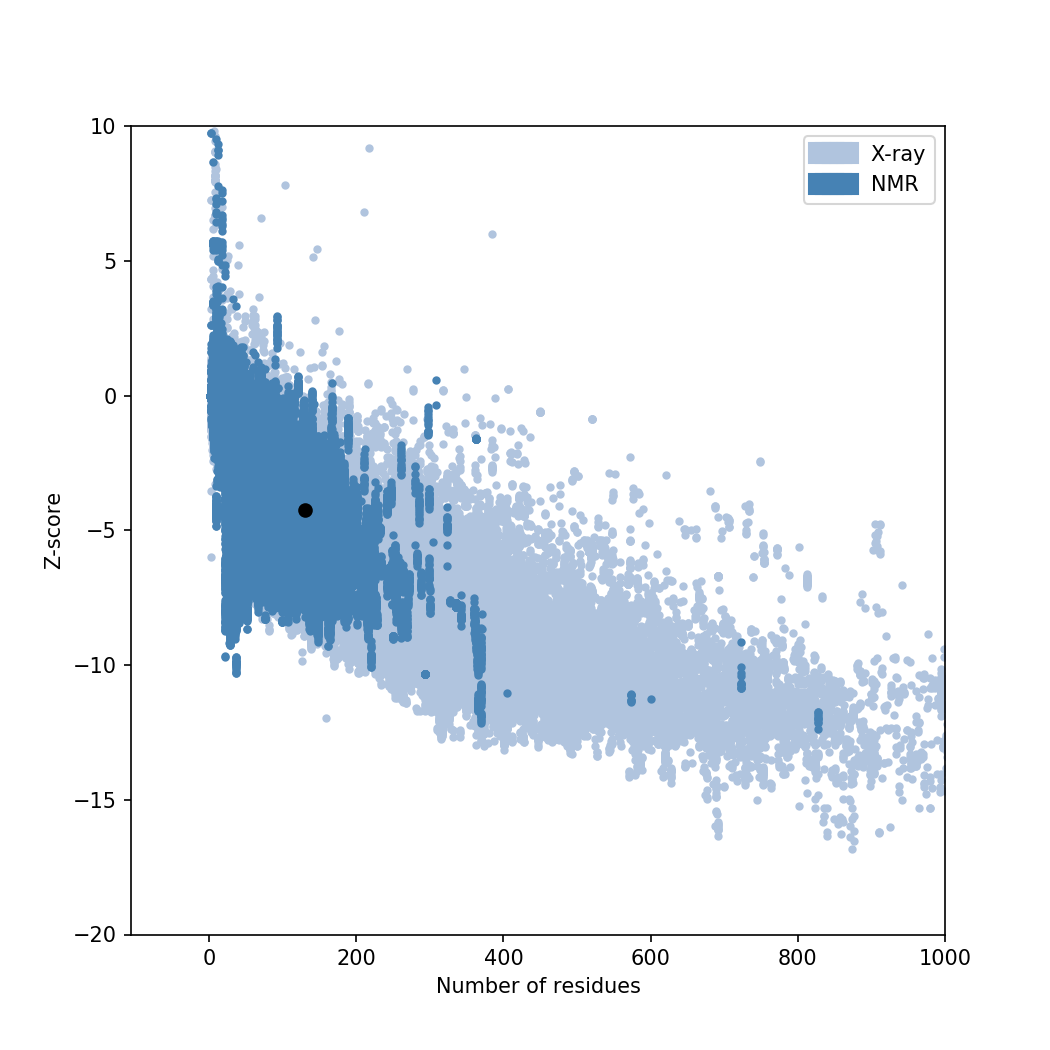** |  |
| **Hypothetical protein (WP_003023105.1)** | **DUF2147 (WP_003023209.1)** |  |
| **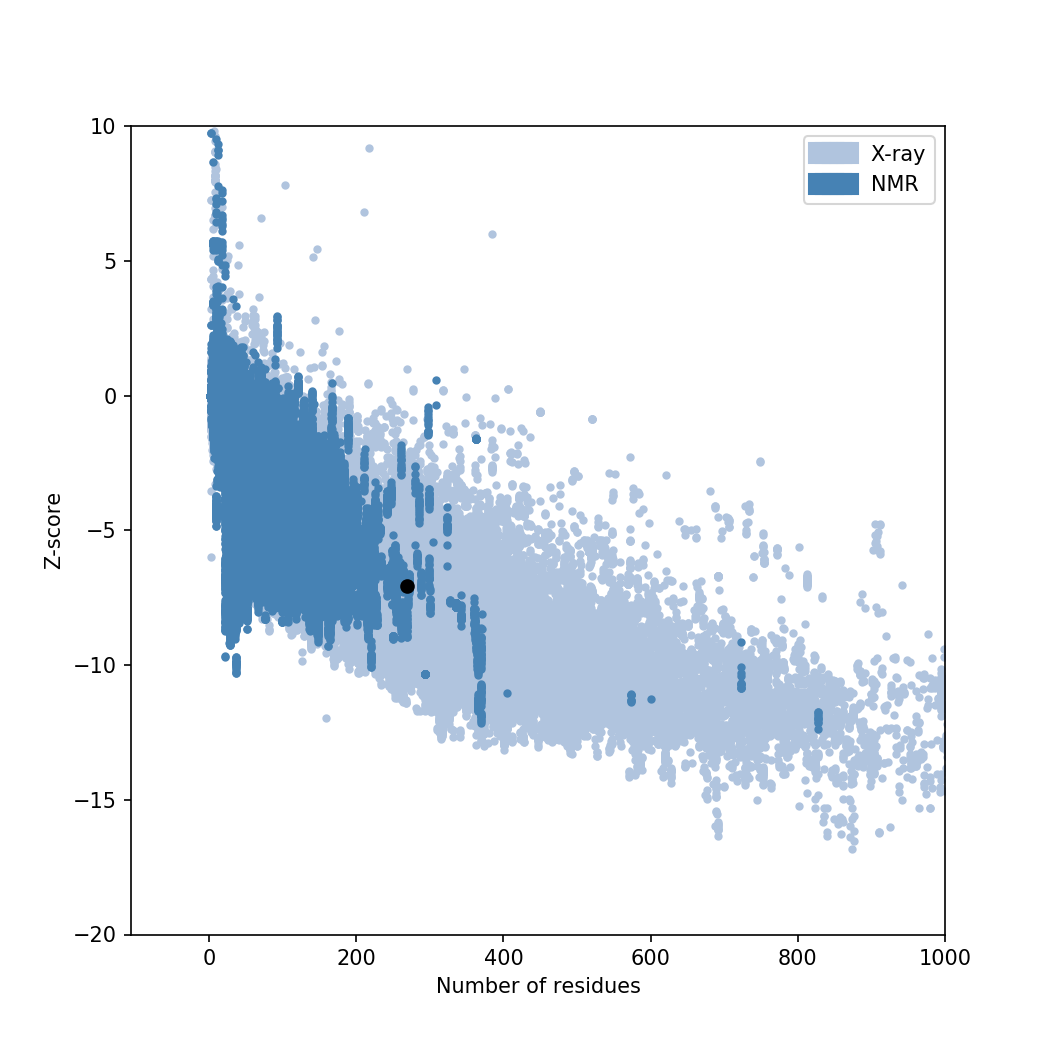** | **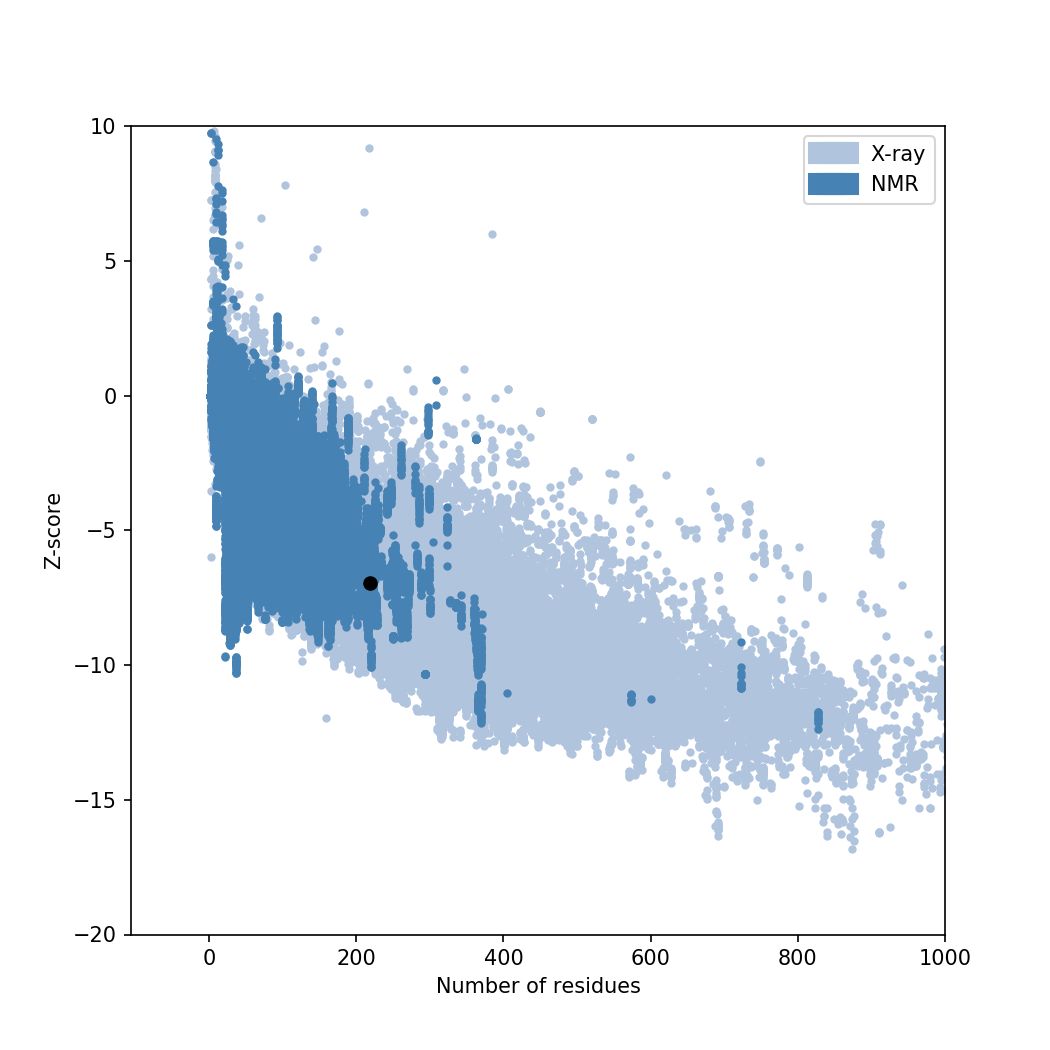** |  |
| **outer membrane protein FopA (WP_003023303.1)** | **Hypothetical protein (WP_003026145.1)** |  |
| **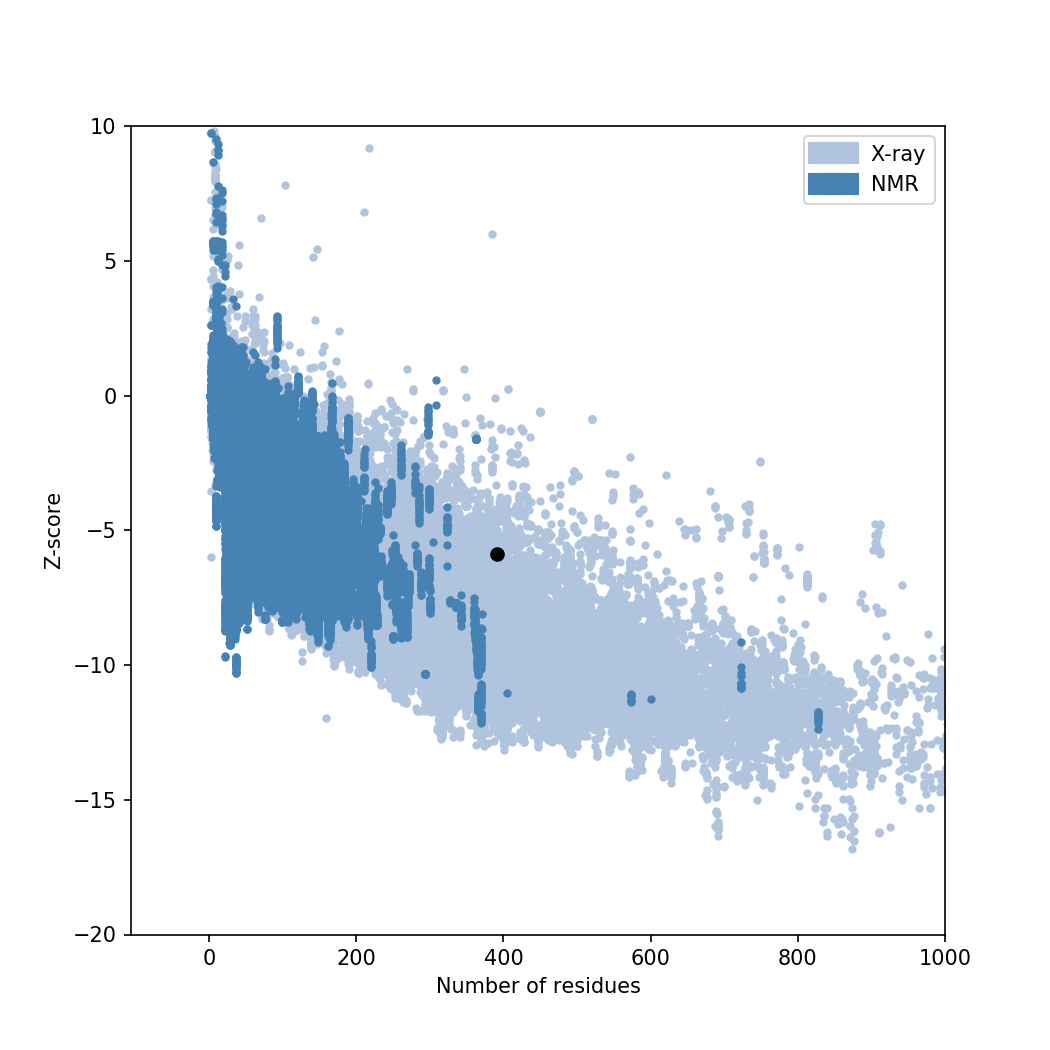** | **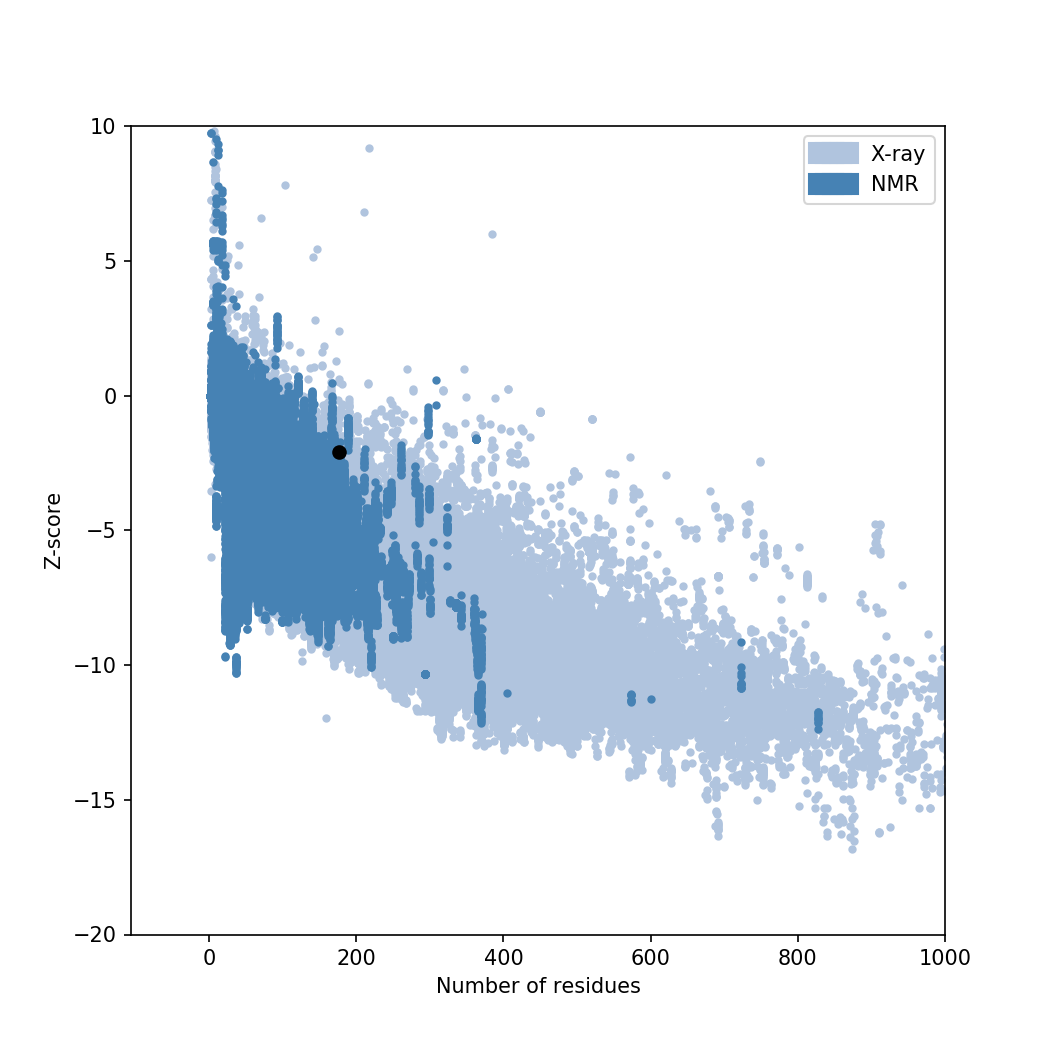** |  |
| **DUF3281 (WP_003026358.1)** | **Hypothetical protein (WP_003029346.1)** |  |
| **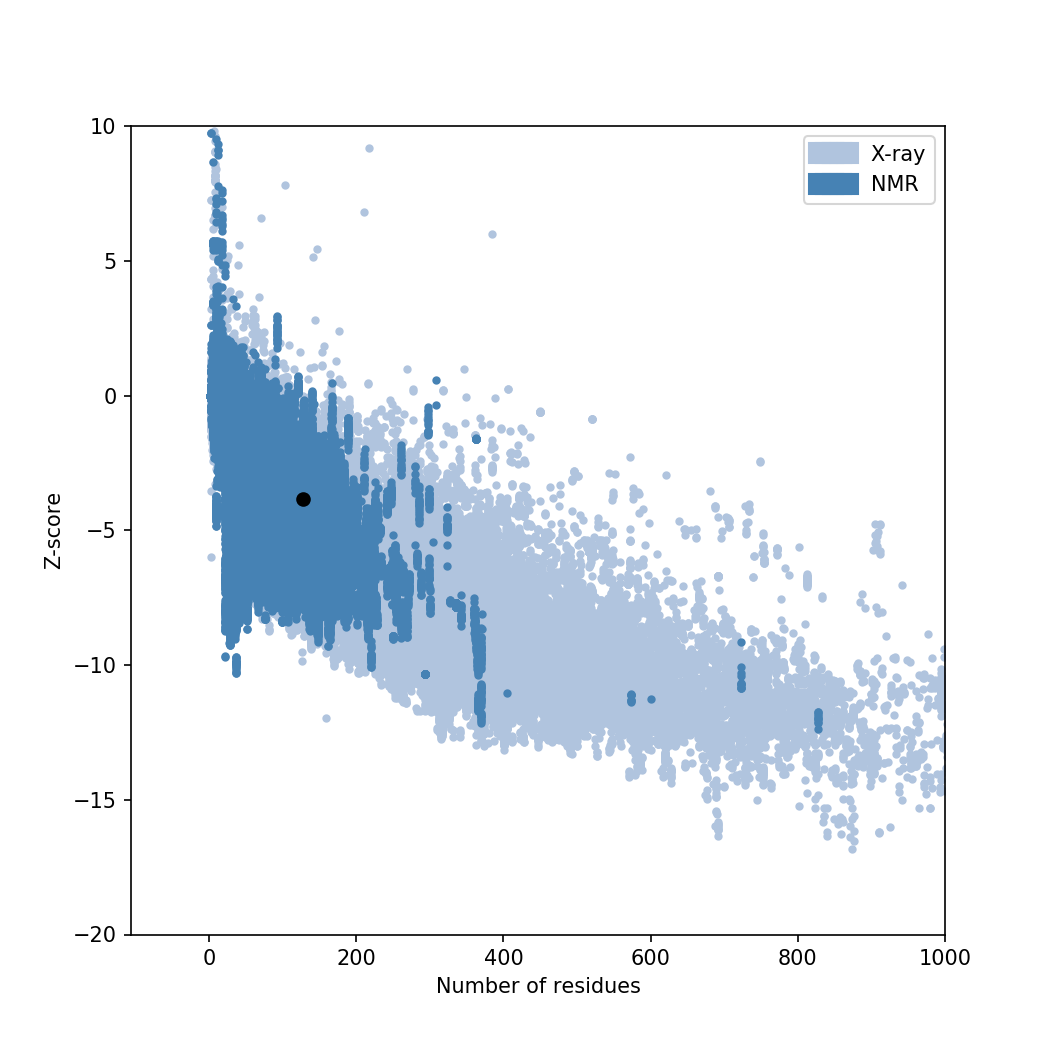** | **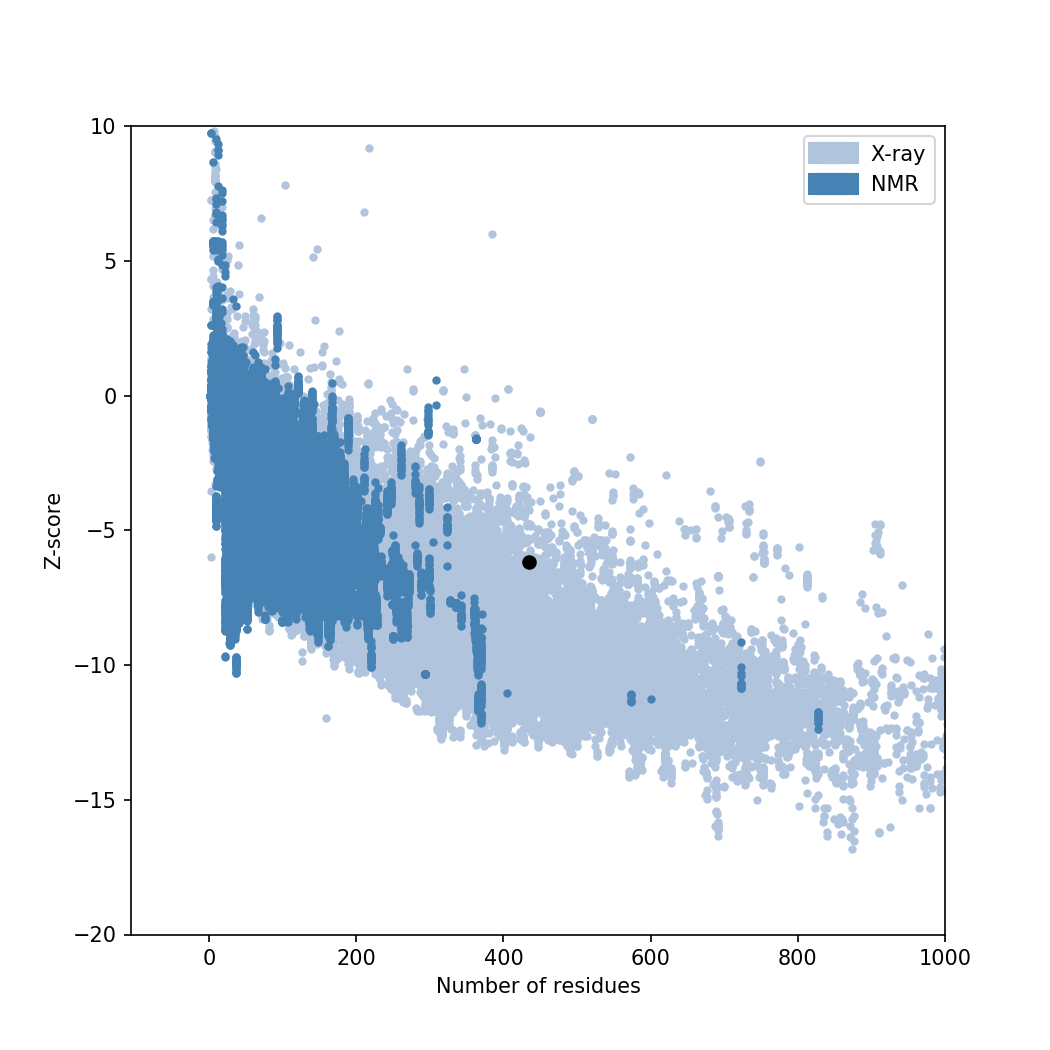** |  |
| **Hypothetical protein (WP_003029578.1)** | **Carbohydrate-binding protein (WP_227644127.1)** |  |
| **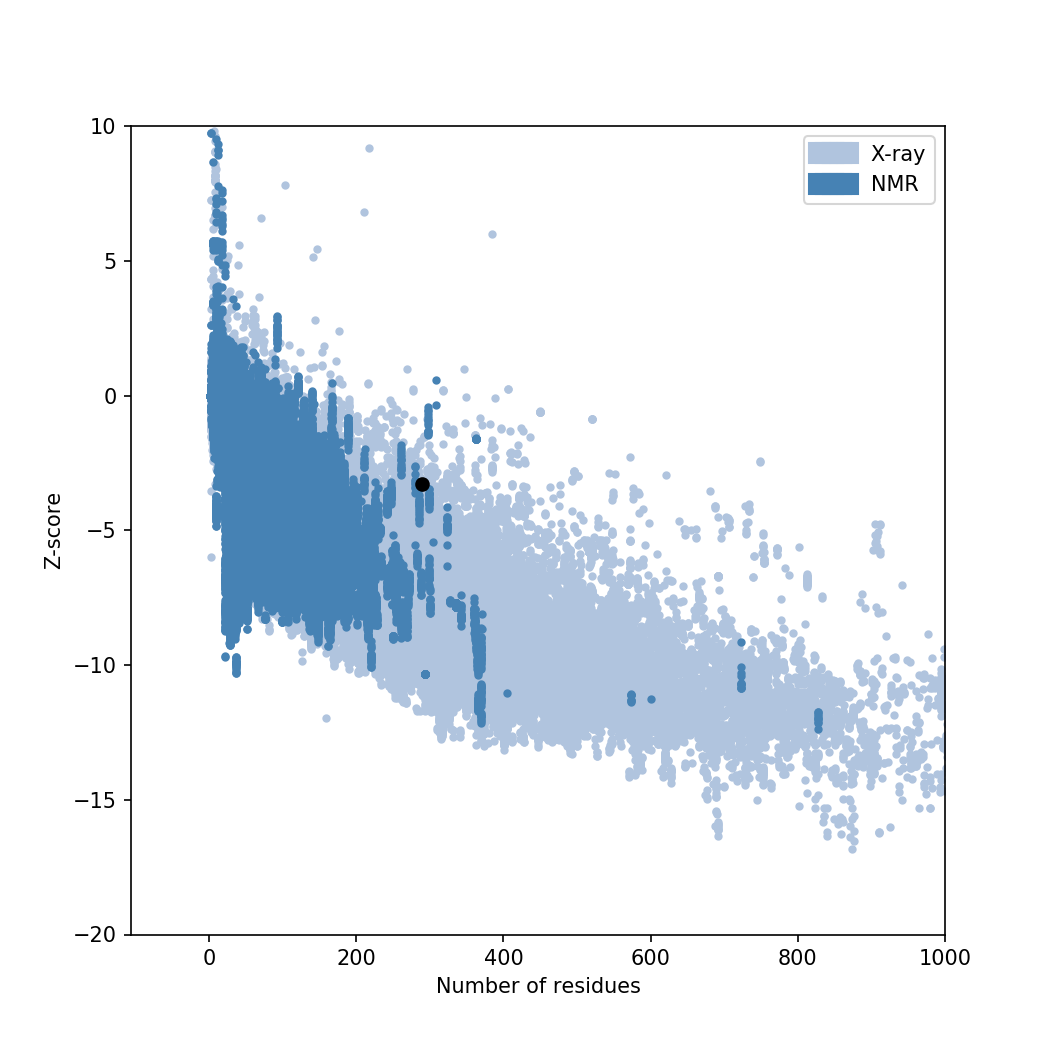** | **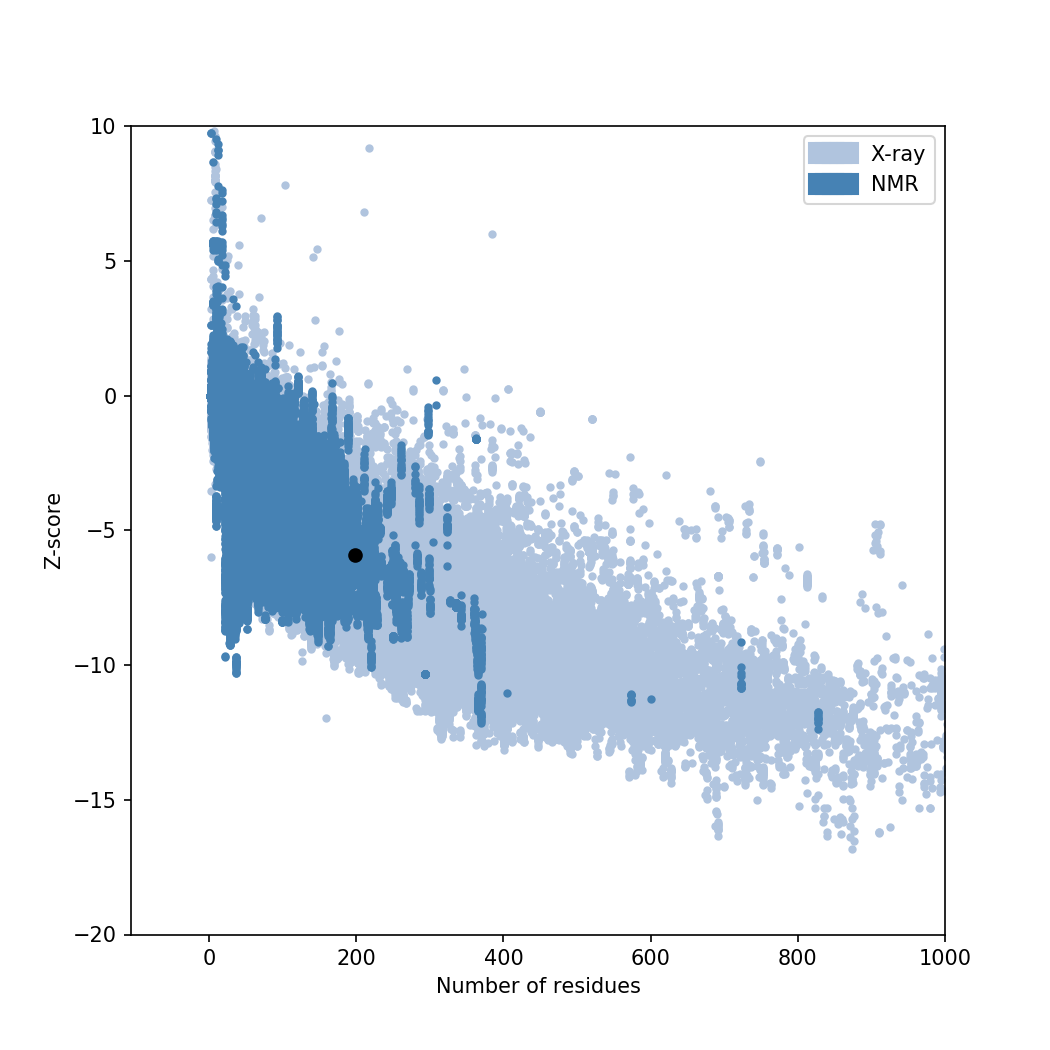** |  |

Table 2. Validation results for the predicted structures of all 12 proteins, assessed through Ramachandran plot analysis.

| **OmpA family protein (WP_003020808.1)** | **PD40 (WP_003021546.1)** |
| --- | --- |
| **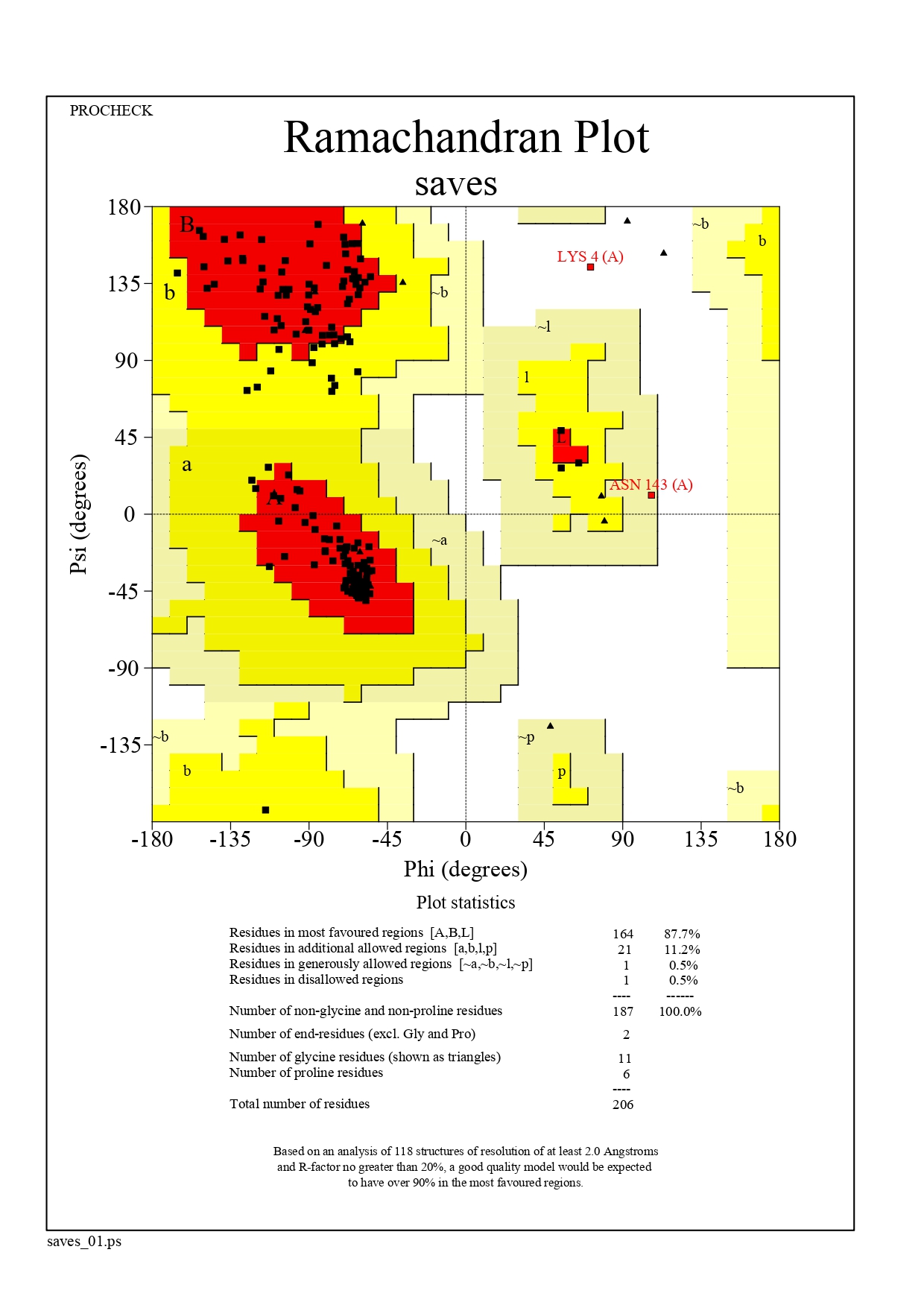** | **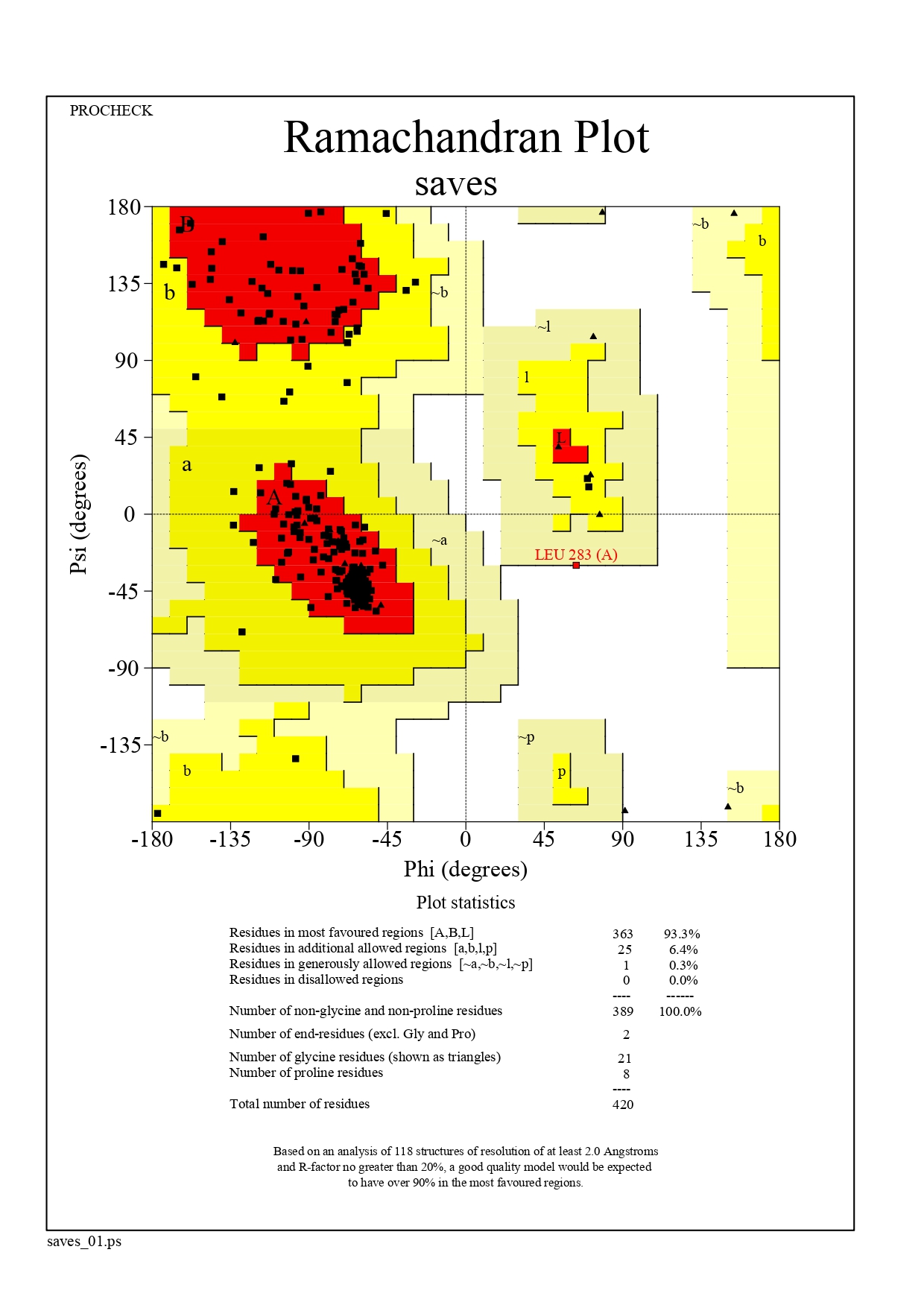** |
| **DUF4124 (WP_003022381.1)** | **Hypothetical protein (WP_003022843.1)** |
| **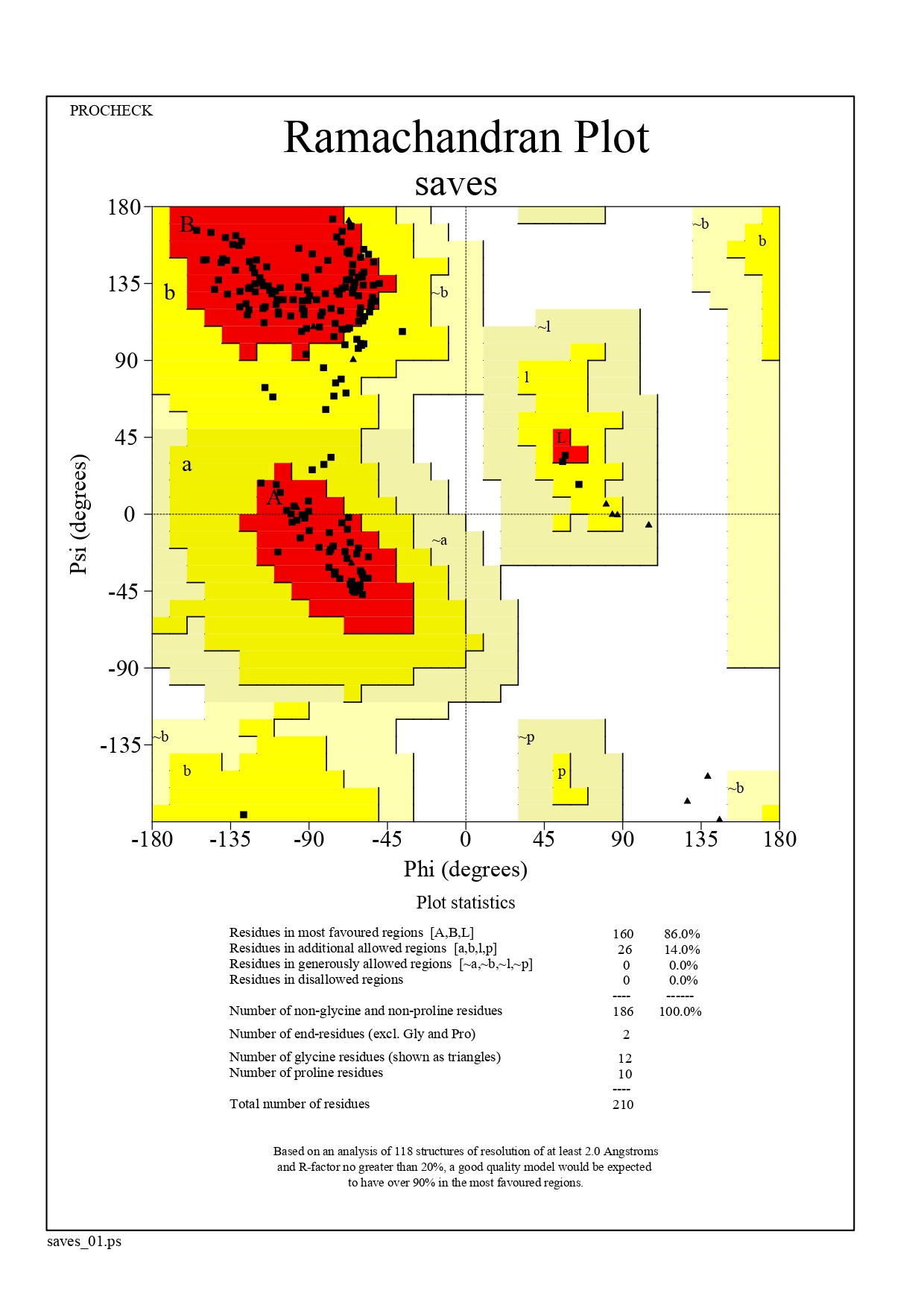** | **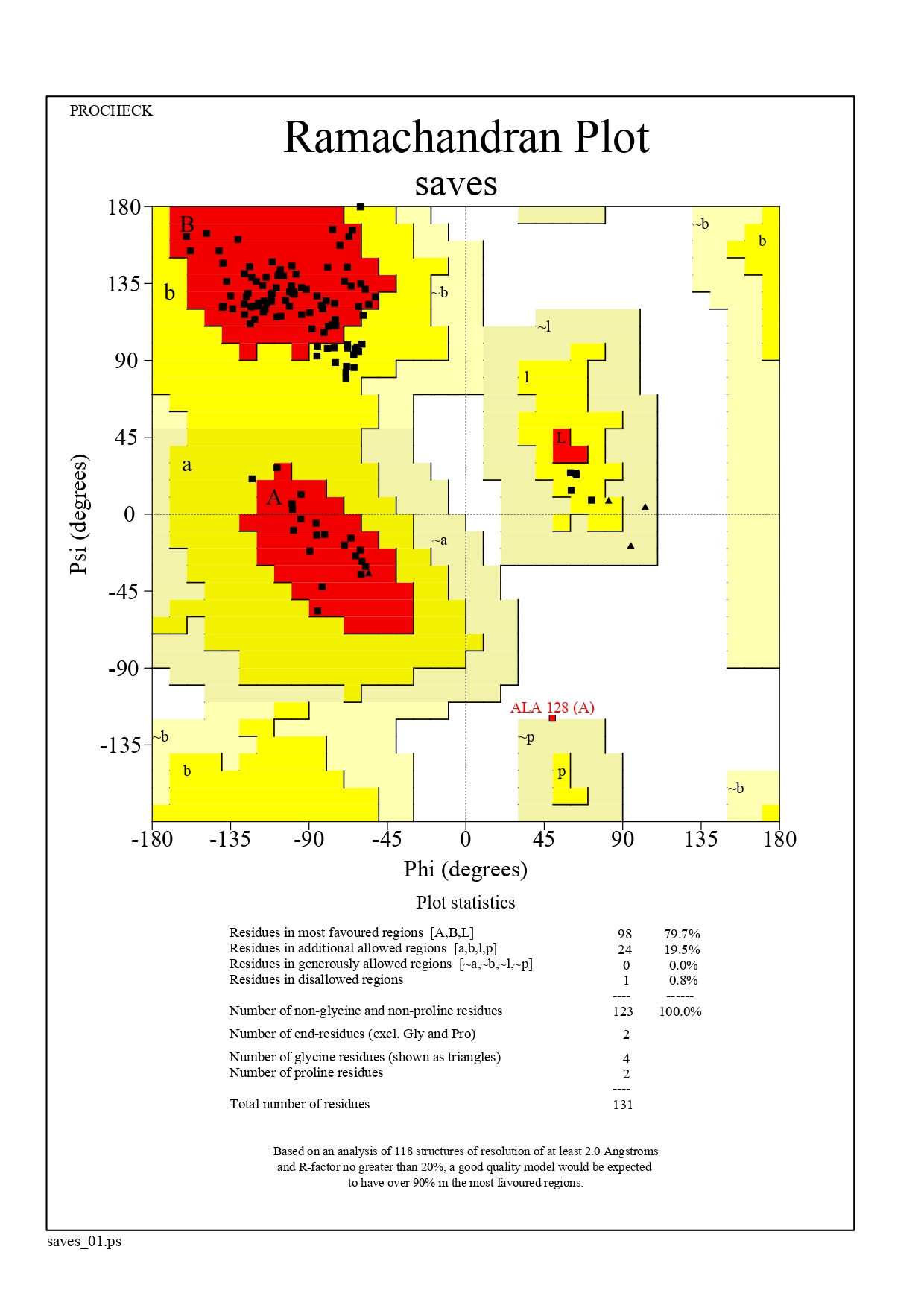** |
| **Hypothetical protein (WP_003023105.1)** | **DUF2147 (WP_003023209.1)** |
| **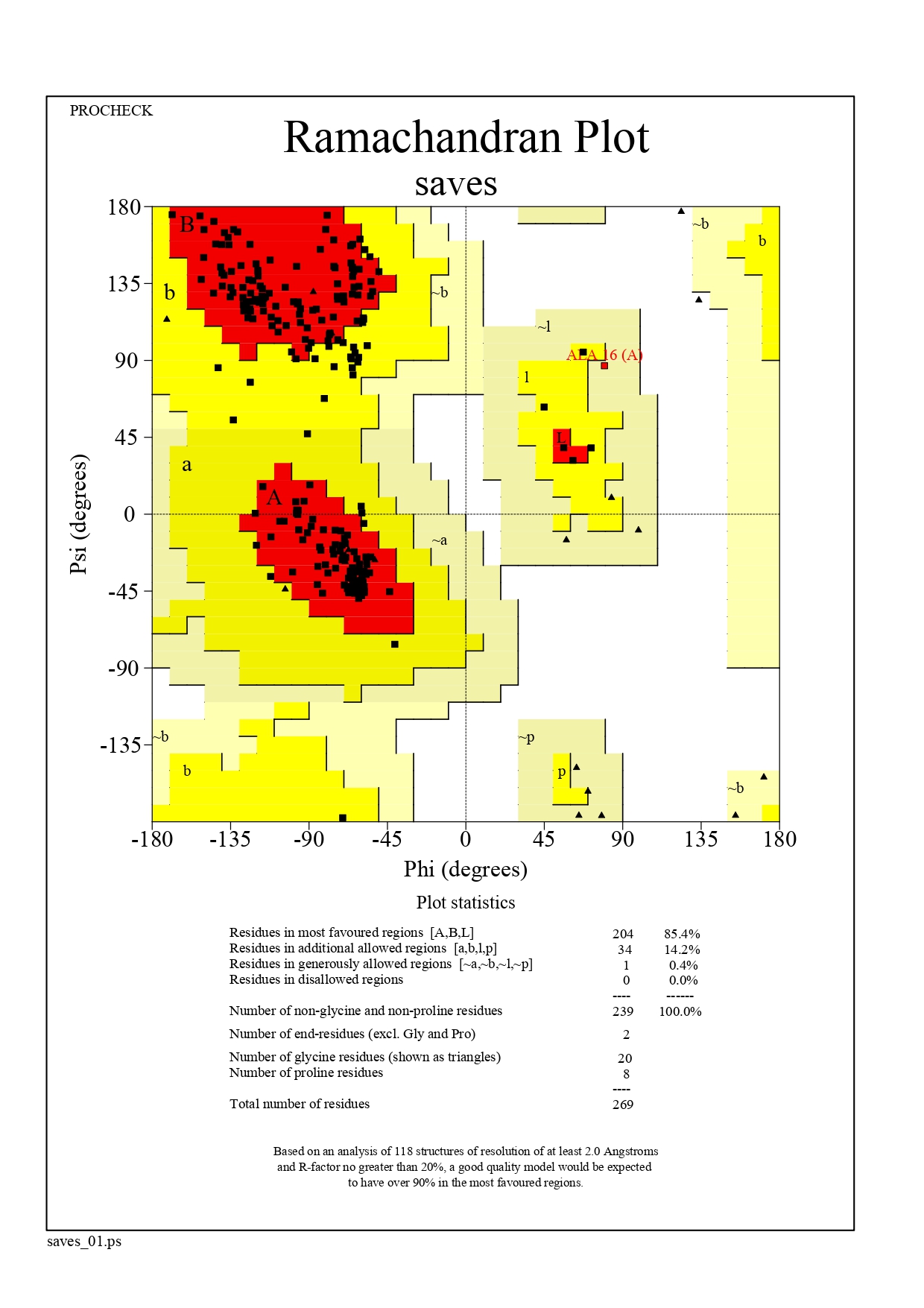** | **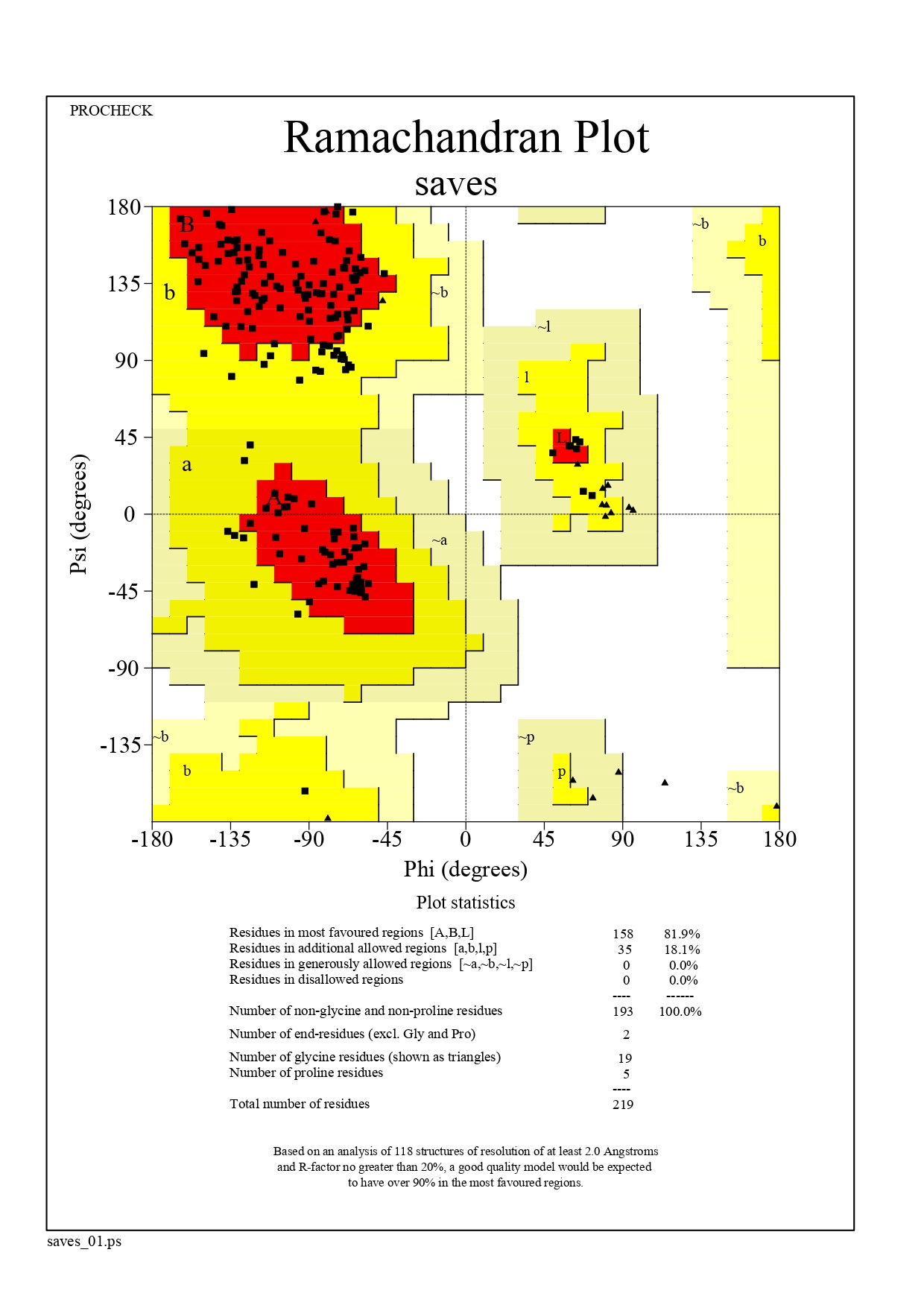** |
| **outer membrane protein FopA (WP_003023303.1)** | **Hypothetical protein (WP_003026145.1)** |
| **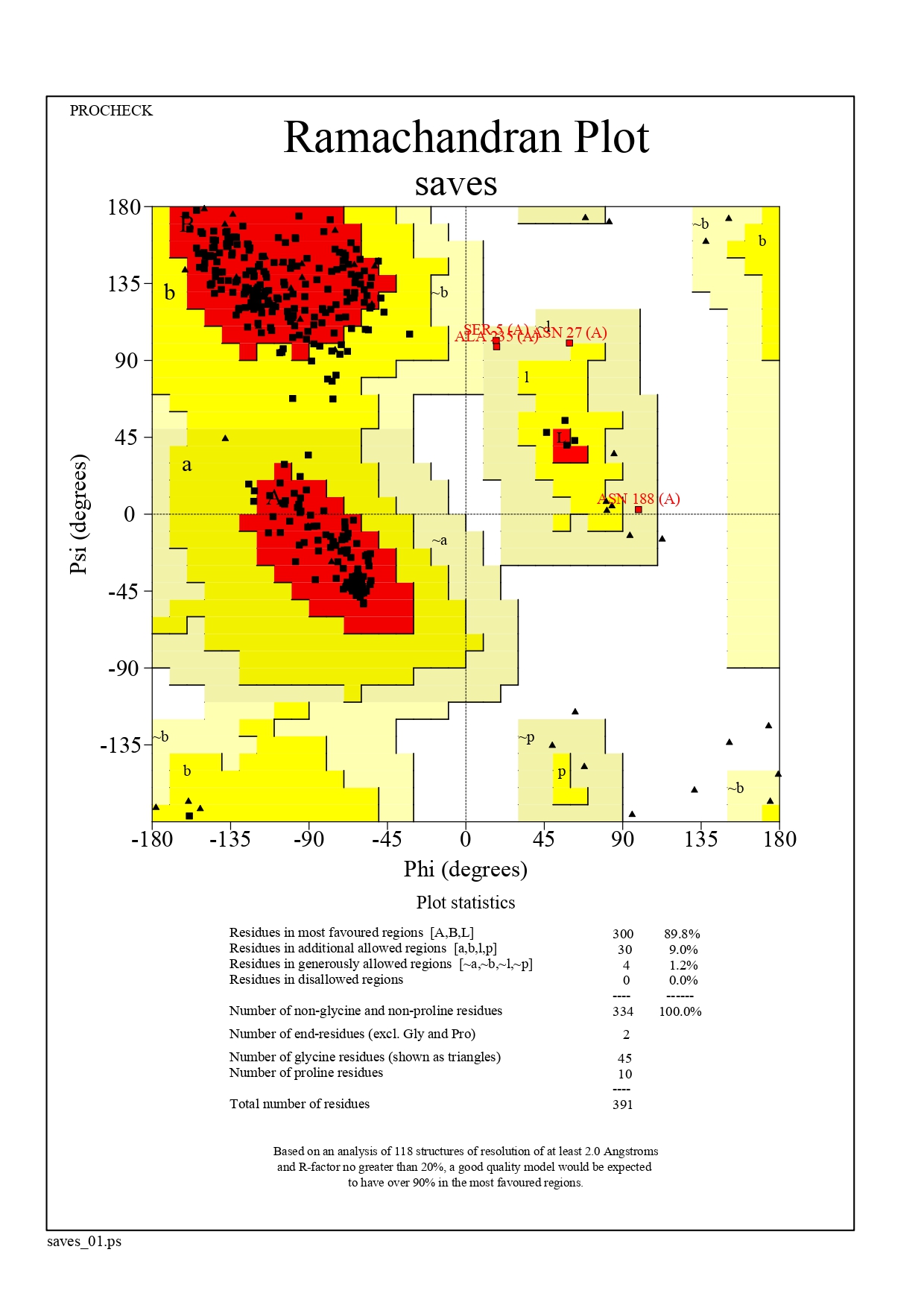** | **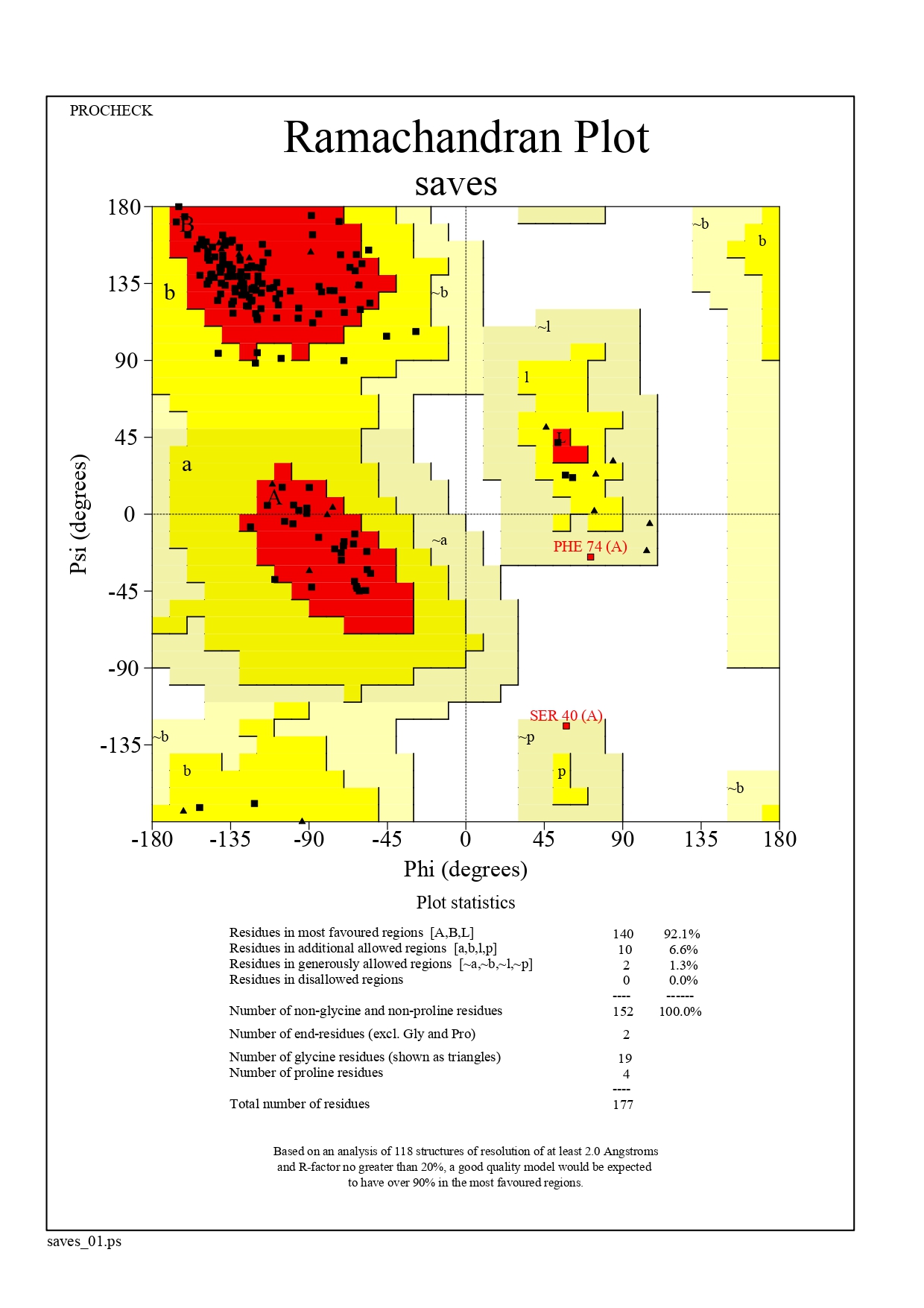** |
| **DUF3281 (WP_003026358.1)** | **Hypothetical protein (WP_003029346.1)** |
| **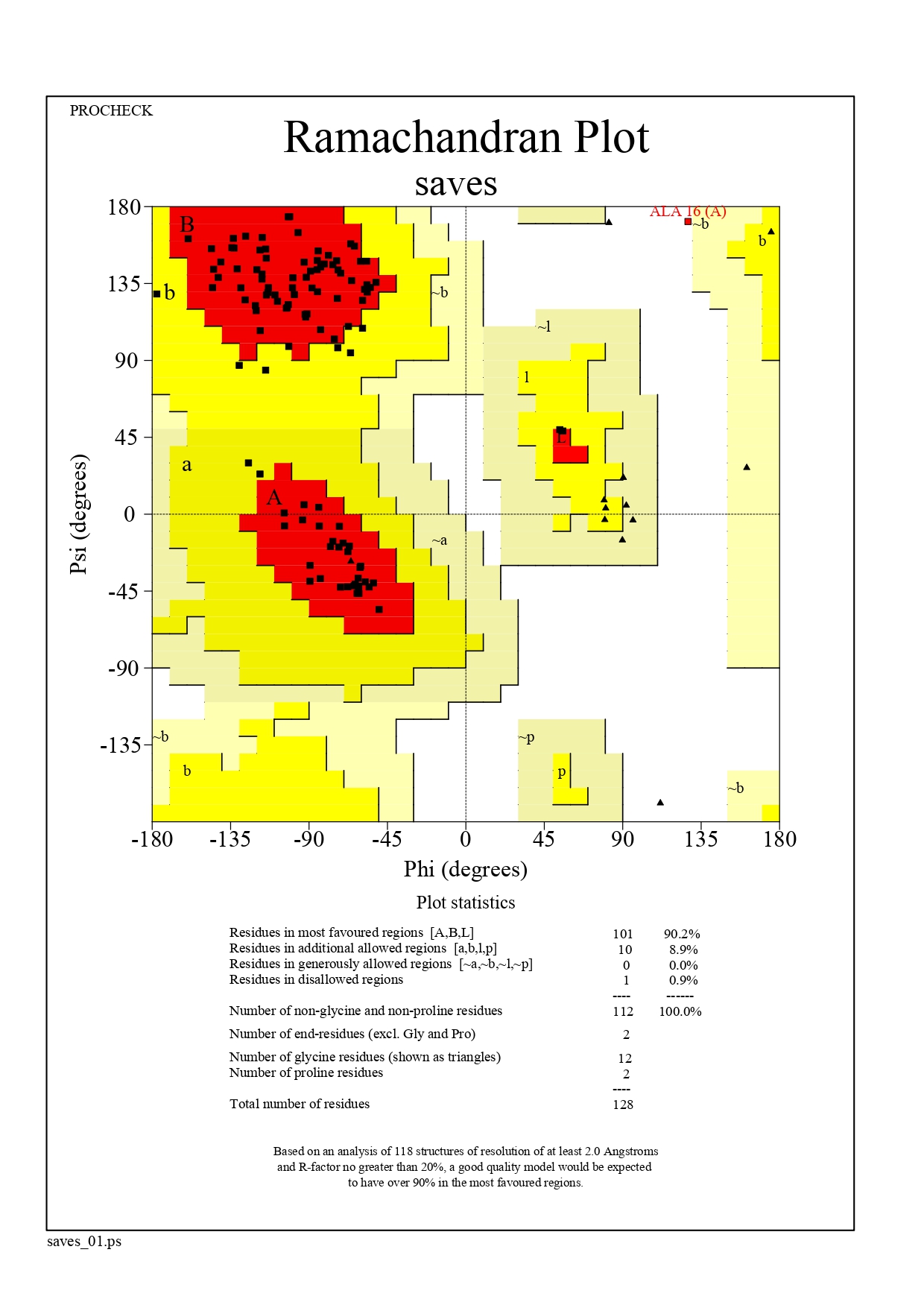** | **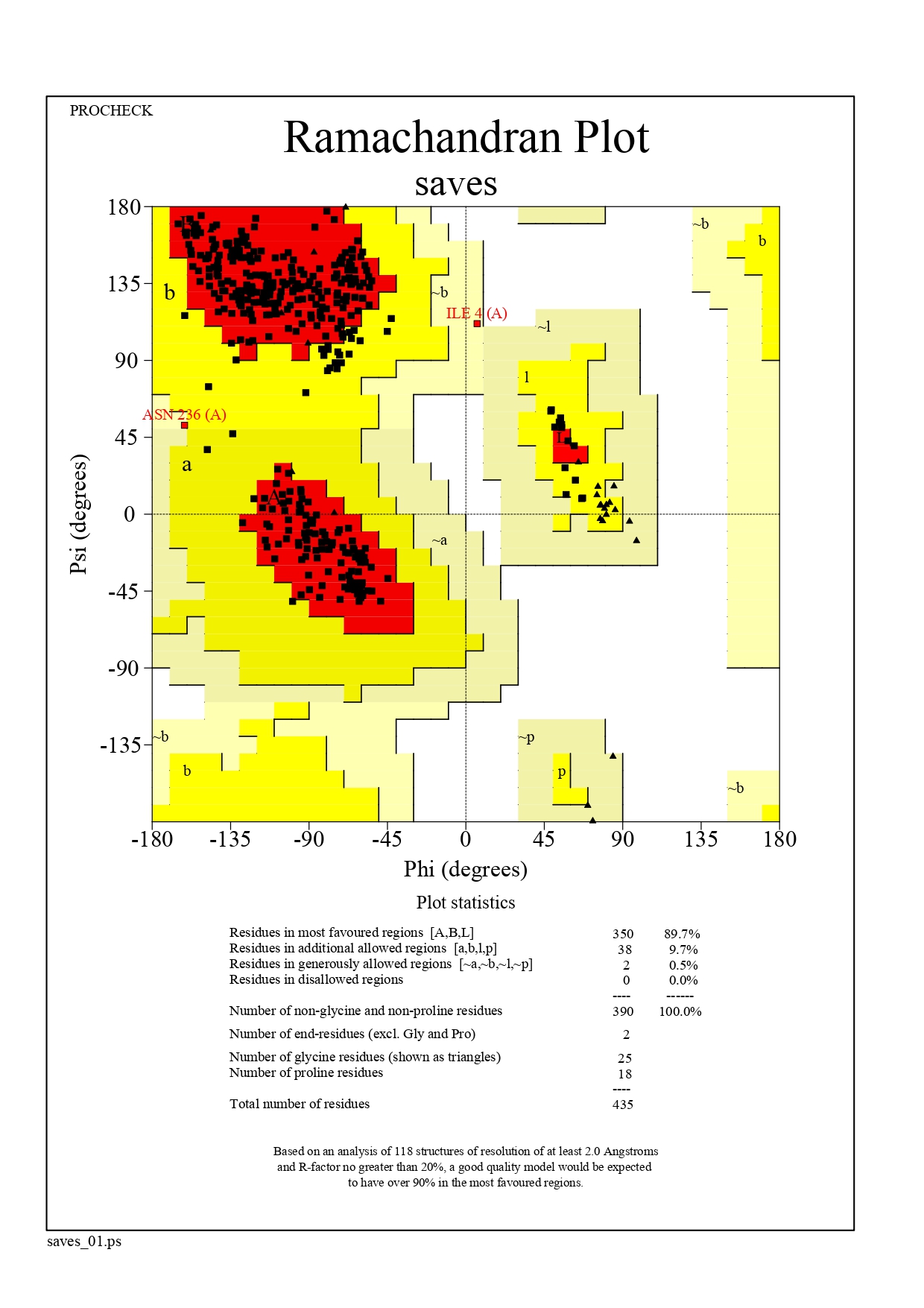** |
| **Hypothetical protein (WP_003029578.1)** | **Carbohydrate-binding protein (WP_227644127.1)** |
| **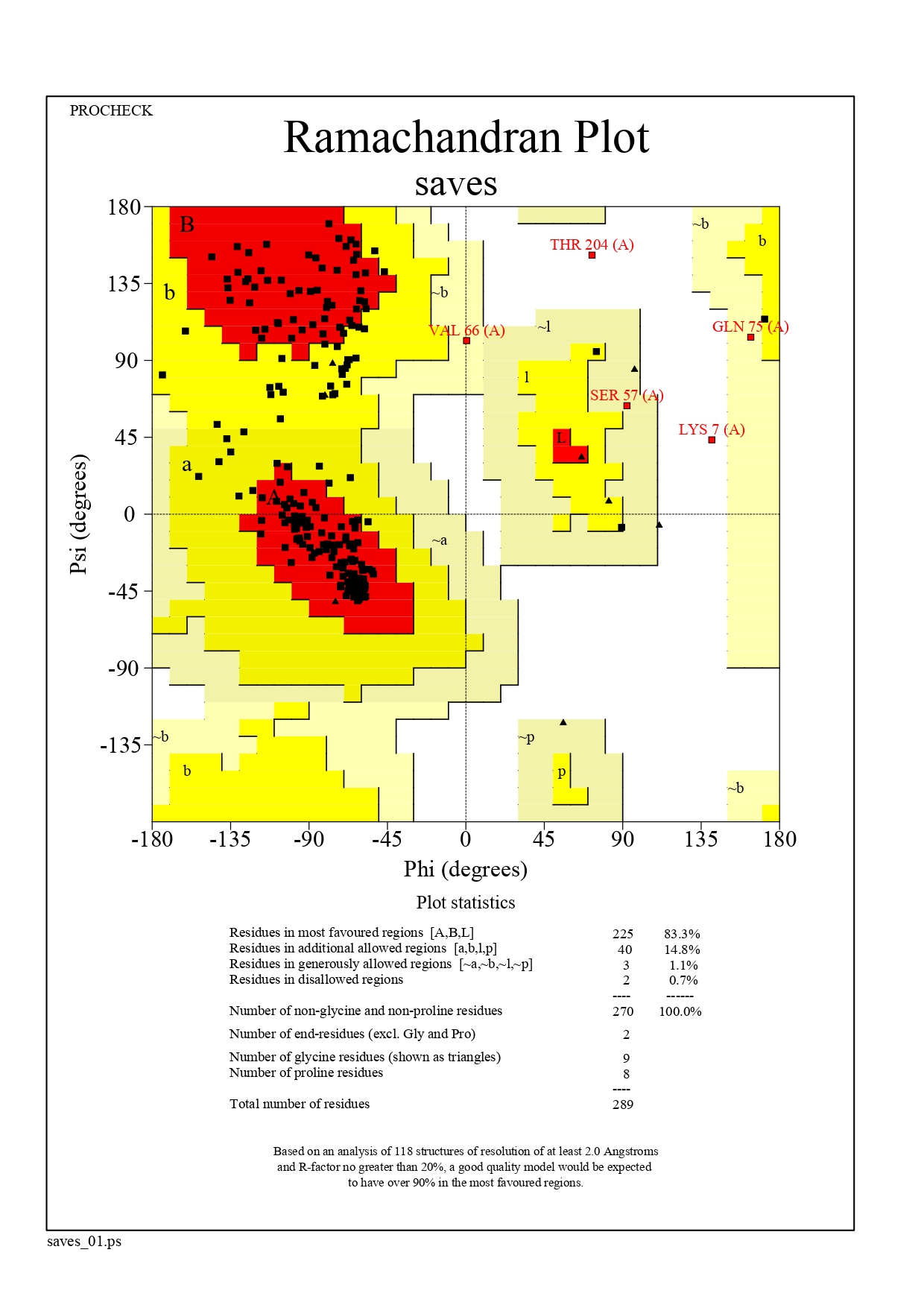** | **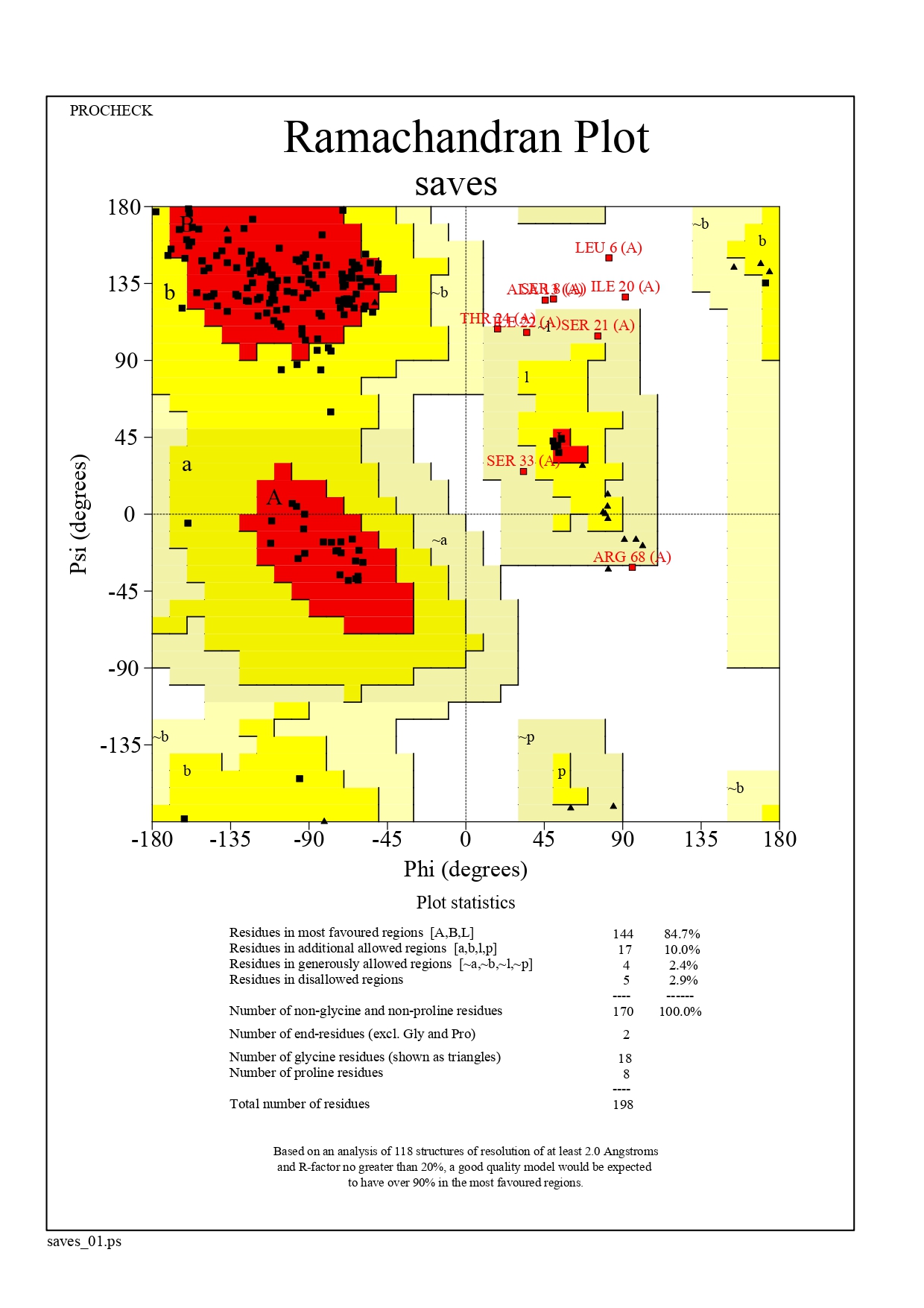** |

Table 3. Validation results for the predicted structures of all 12 proteins, assessed through VERIFY 3D analysis

| **OmpA family protein (WP_003020808.1)** | **PD40 (WP_003021546.1)** |
| --- | --- |
| **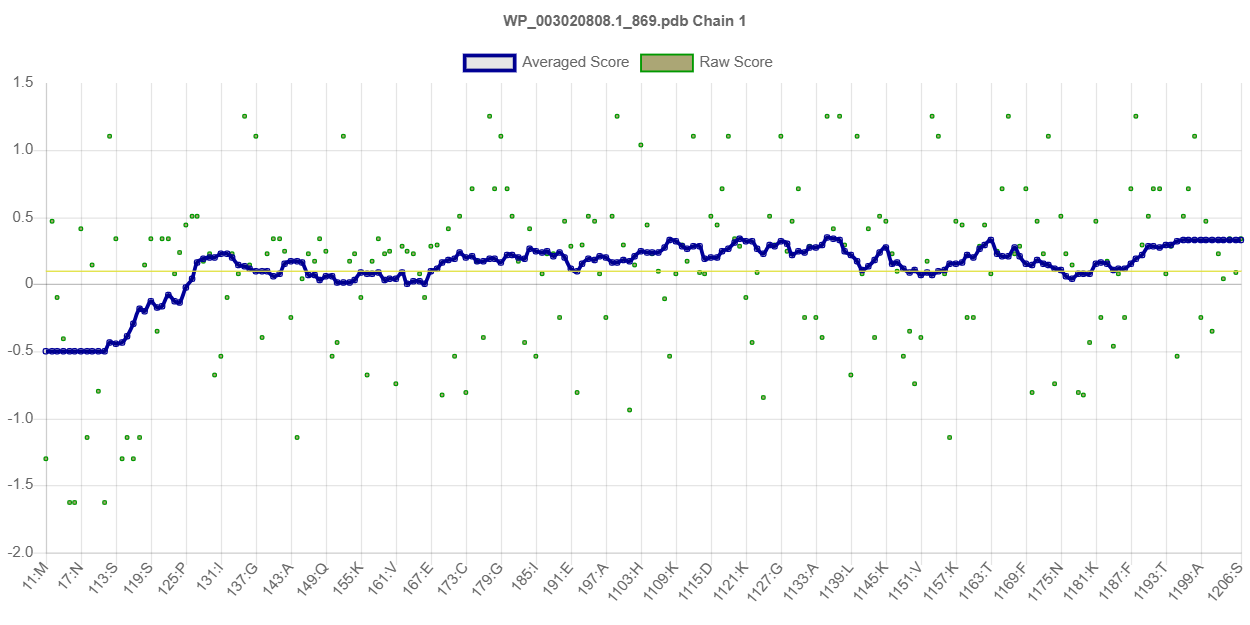** | **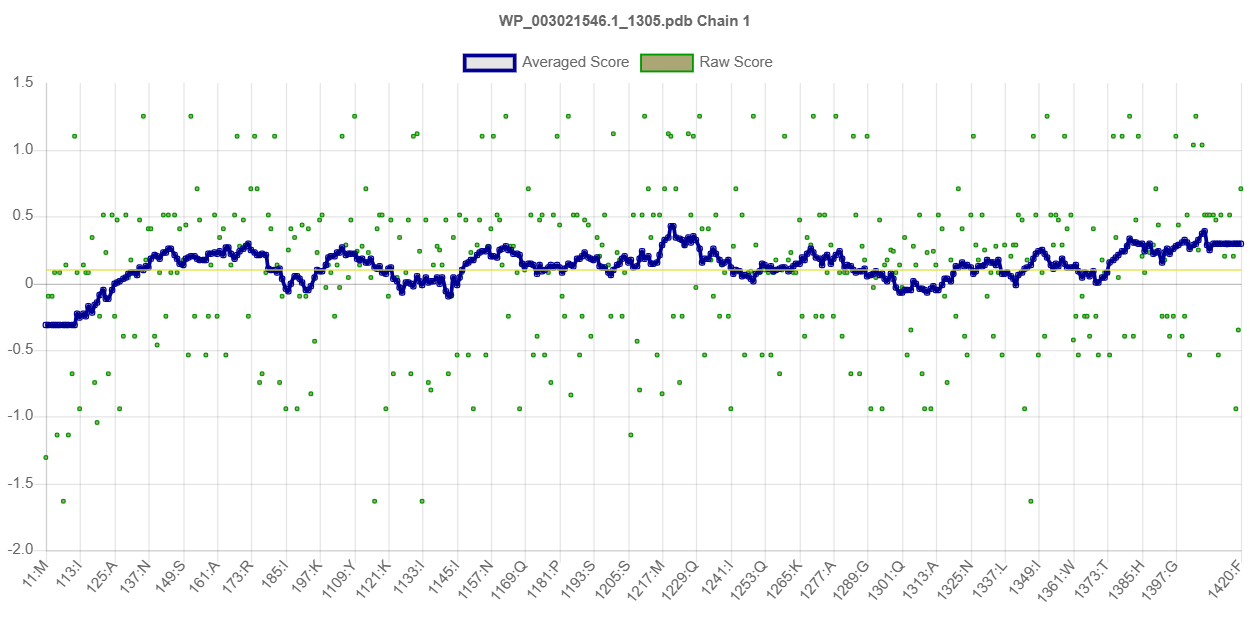** |
| **DUF4124 (WP_003022381.1)** | **Hypothetical protein (WP_003022843.1)** |
| **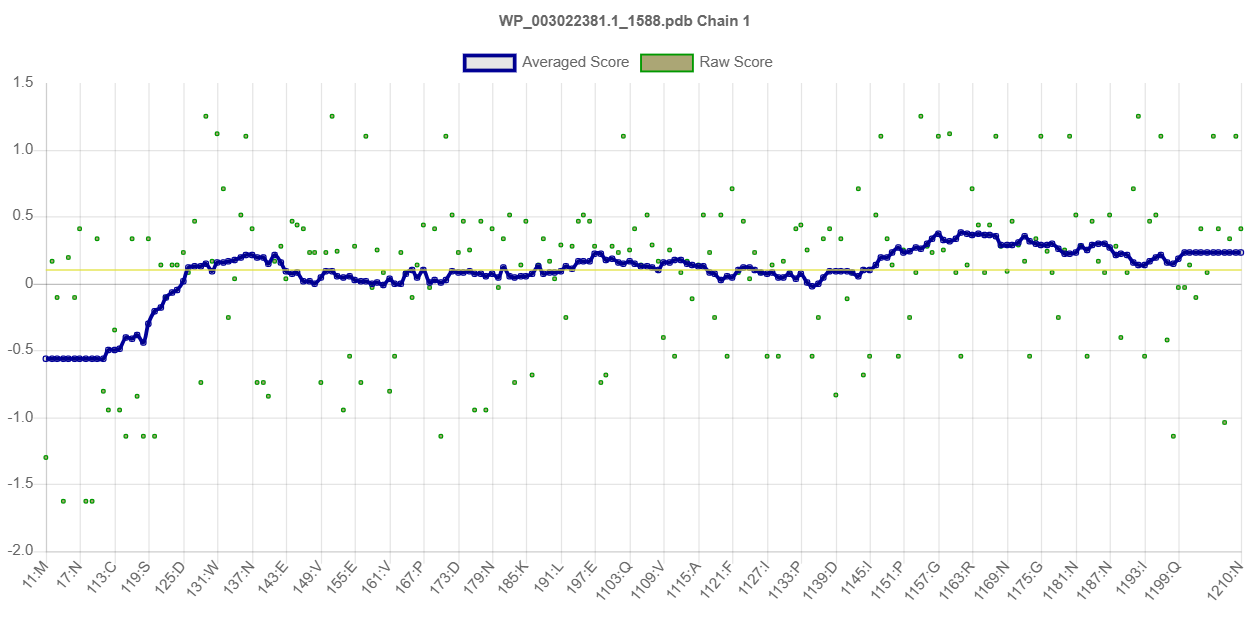** | **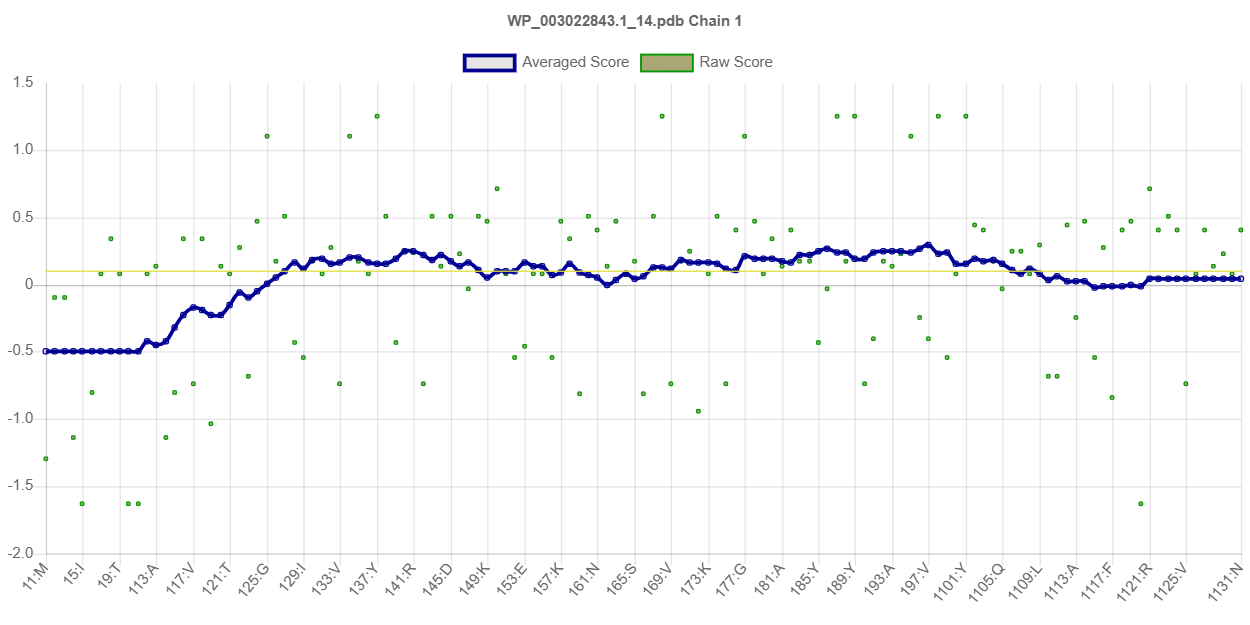** |
| **Hypothetical protein (WP_003023105.1)** | **DUF2147 (WP_003023209.1)** |
| **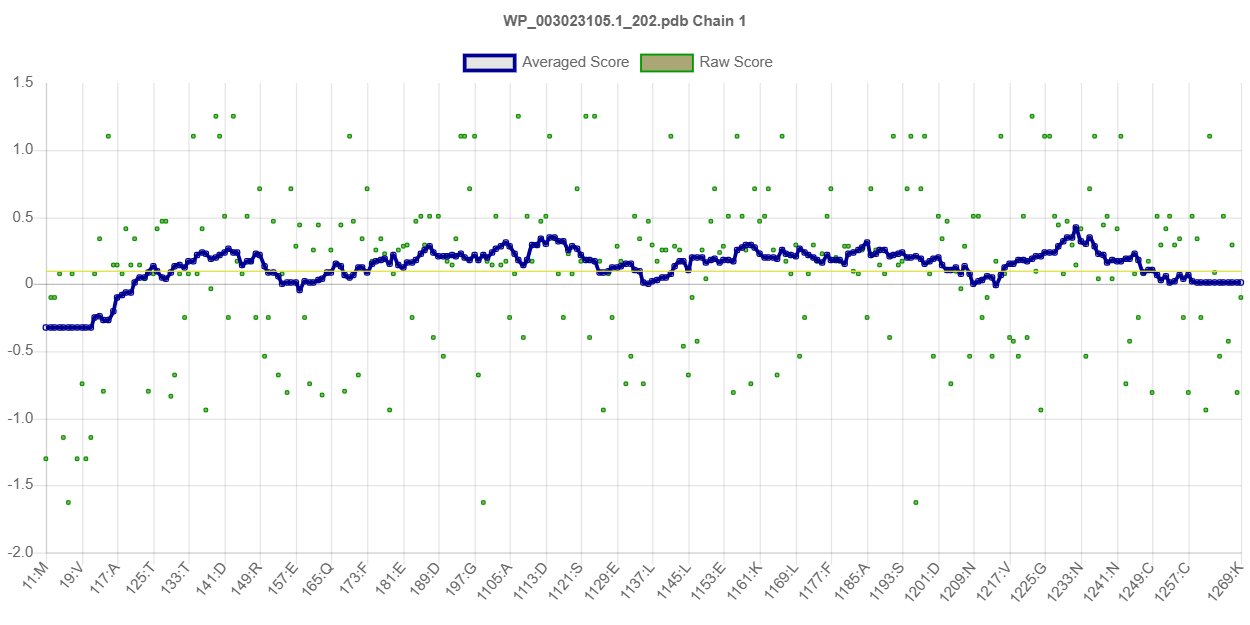** | **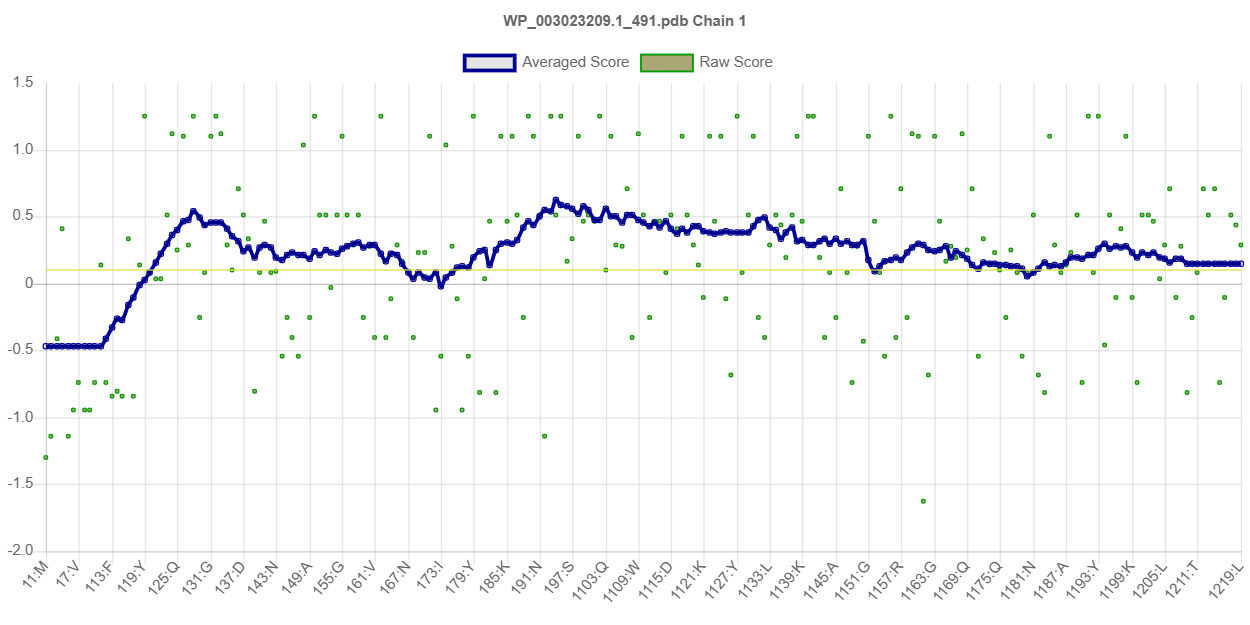** |
| **outer membrane protein FopA (WP_003023303.1)** | **Hypothetical protein (WP_003026145.1)** |
| **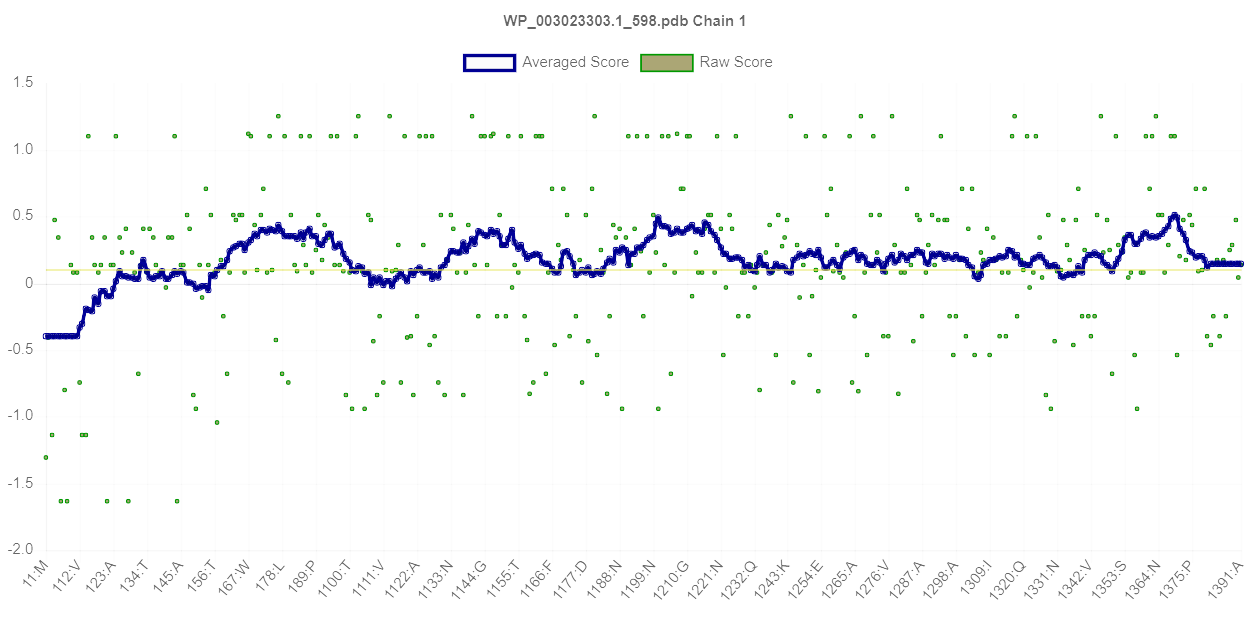** | **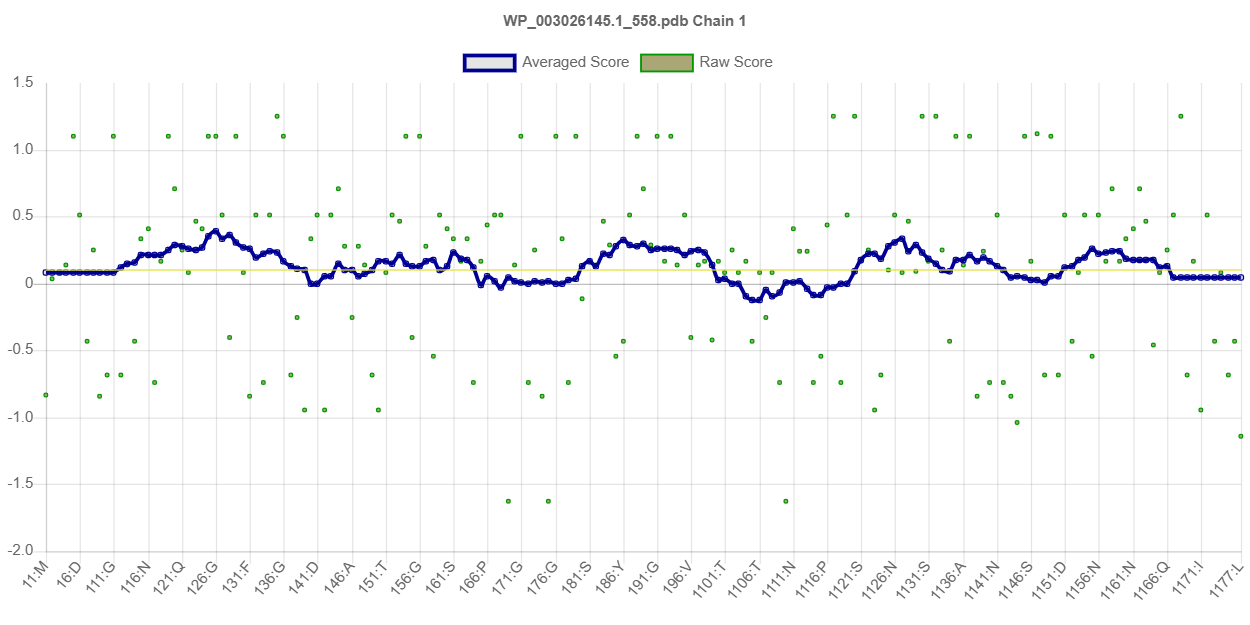** |
| **DUF3281 (WP_003026358.1)** | **Hypothetical protein (WP_003029346.1)** |
| **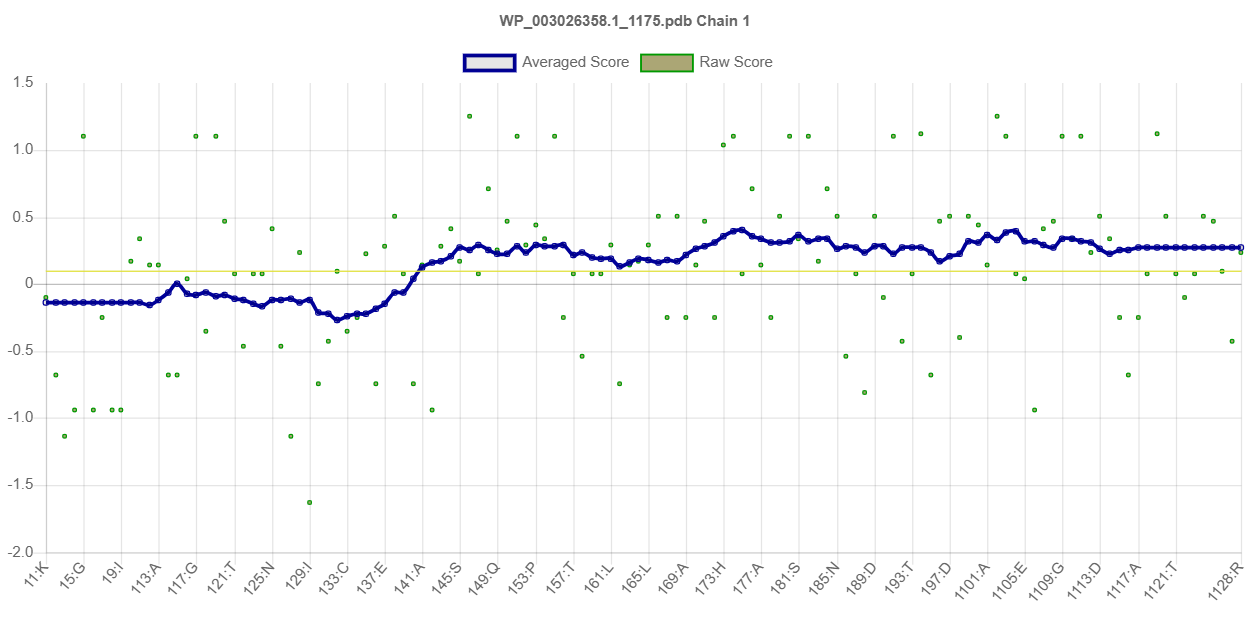** | **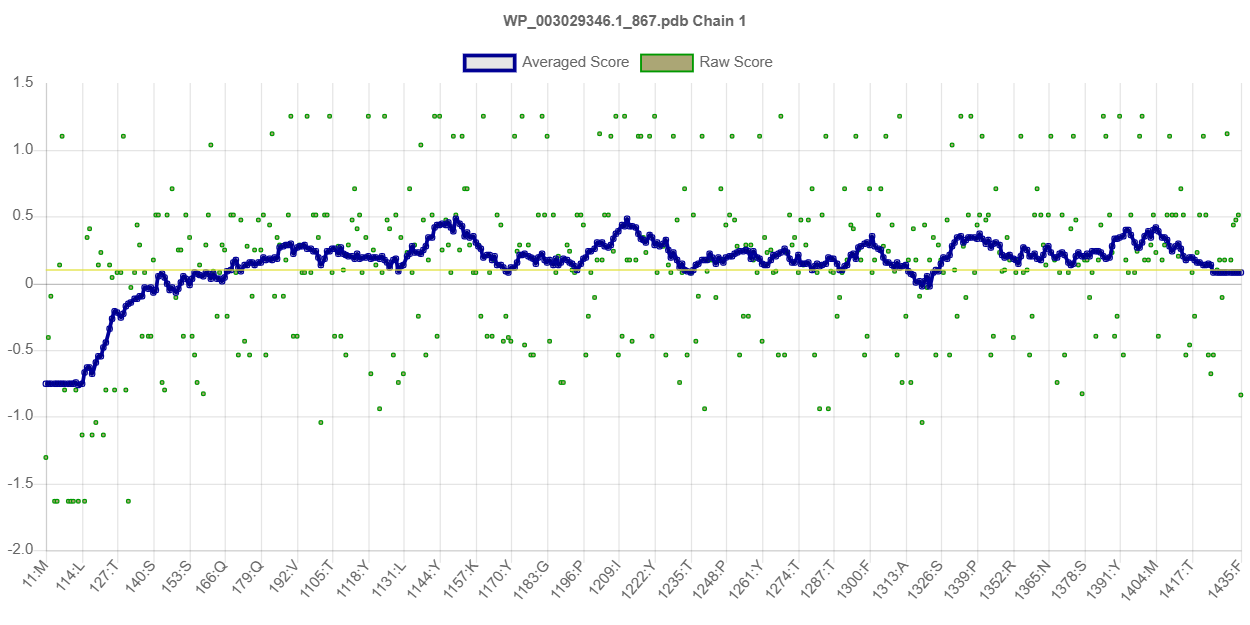** |
| **Hypothetical protein (WP_003029578.1)** | **Carbohydrate-binding protein (WP_227644127.1)** |
| **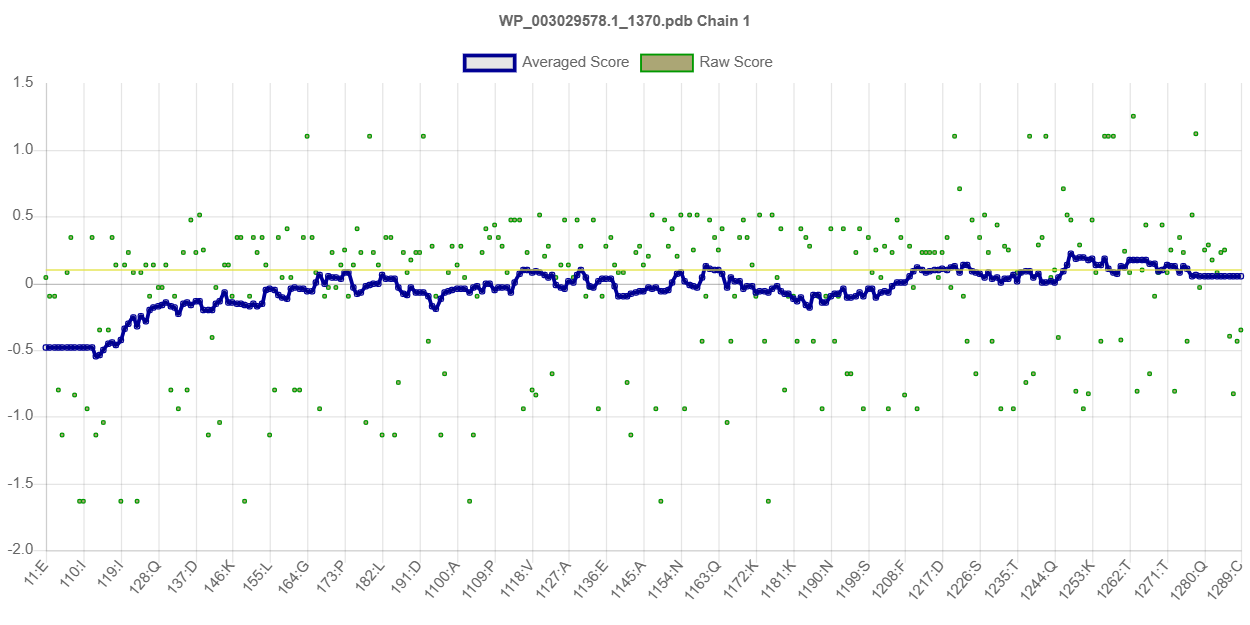** | **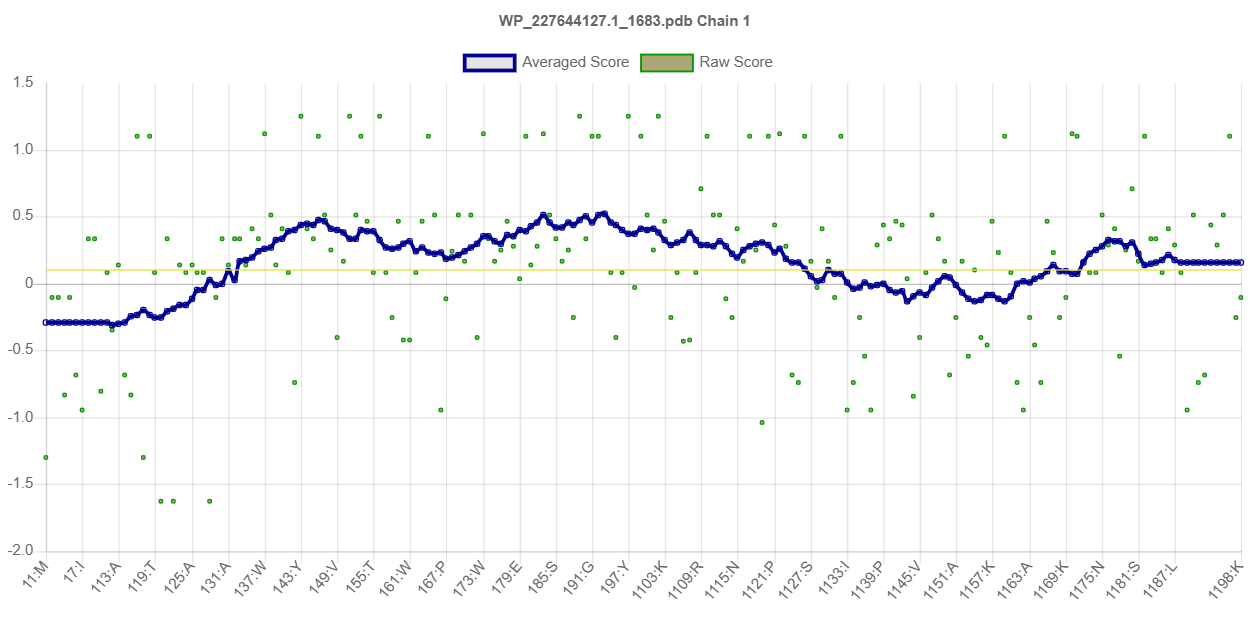** |
