# Supplementary material for: Development of a multi-epitope vaccine from outer membrane proteins and identification of novel drug targets against Francisella tularensis: an In Silico approach
Source: Front Immunol. 2025 Apr 3;16:1479862. doi: 10.3389/fimmu.2025.1479862 (PMC12003292; doi:10.3389/fimmu.2025.1479862)
Supplement: Supplementary file 3 [file DataSheet3.docx]

**Supplementary Table 1**. Linear and conformational B-cell epitopes of 12 putative immunogenic targets of *F. tularensis,* determined by BepiPred (threshold ≥0.6) and Ellipro (threshold ≥0.8), respectively.

| **Accession number (Protein)** | **Linear B cell epitopes** | **Start-End** | **Antigenicity** | **Conservancy %** | **Conformational B cell epitopes** | **Color in 3D Modeling** |
| --- | --- | --- | --- | --- | --- | --- |
| **WP_003020808.1**  **(**OmpA family protein**)** | STRPDNSDLIKDKYAGVDSSQALEMSSQIYGSDKLSSDQVEQMKKELMNINCR | 23-75 | 0.6334 | 100 | A:R5, A:I6, A:I7, A:N8, A:L9, A:A10, A:V11, A:I12, A:G13, A:S14, A:M15, A:L16 | Red |
|  |  |  |  |  | A:E162, A:T164, A:Y167, A:E169, A:F170, A:C171, A:K172, A:D173, A:G174, A:V175, A:N176, A:D177, A:A178, A:C179, A:M180 | Orange |
|  |  |  |  |  | A:G53, A:K56, A:L57, A:S58, A:S59, A:D60, A:Q61, A:E63 | Yellow |
| **WP_003021546.1 (PD40 domain-containing protein)** | NHNAKLQANDTIKYEIKQKQNIPWKSL | 60-86 | 0.8526 | 100 | A:T4, A:L5, A:T6, A:I7, A:A8, A:L9, A:L10, A:G11, A:T12, A:I13, A:A14, A:T15, A:T16 | Yellow |
|  |  |  |  |  | A:K108, A:A111, A:E112, A:D114, A:E115, A:I116, A:N117, A:N118, A:N119, A:I120, A:K121, A:K122, A:L123, A:E124, A:S125, A:Q126, A:K127, A:K128, A:L129, A:G130, A:W131, A:R132, A:I133, A:K134, A:V135, A:V136, A:A137, A:E138, A:Q139, | Orange |
|  |  |  |  |  | A:D201, A:S202, A:T204, A:S205, A:L206, A:N207, A:V209, A:D210 | Blue |
|  |  |  |  |  | A:A295, A:S296, A:P297, A:R298, A:T299, A:Q301, A:S302, A:E305, A:A306, A:I309, A:D310, A:R312, A:A313, A:N314, A:N315 | Green |
|  |  |  |  |  | A:I85, A:P86, A:N87, A:S88, A:I89 | Red |
|  |  |  |  |  | A:D98, A:A100, A:A103, A:Q104 | Cyan |
| **WP_003022381.1 (DUF4124 domain-containing protein)** | DTKVYSWR | 25-32 | 1.6411 | 100 | A:S208, A:G209, A:N210 | Yellow |
|  | VFSEEKPNDDVDYR | 39-82 | 0.7754 | 100 | A:S2, A:K3, A:F4, A:H5 | Cyan |
|  |  |  |  |  | A:N203, A:T204, A:G205, A:N206, A:Y207 | Orange |
|  |  |  |  |  | A:K6, A:N7, A:I8, A:F9, A:S10, A:V11, A:I12, A:C13, A:I14, A:L15, A:S16, A:F17 | Green |
|  |  |  |  |  | A:Q200, A:A201, A:K202 | Red |
|  |  |  |  |  | A:A66, A:P67, A:Q68, A:N69, A:L70, A:G71, A:N72, A:D73, A:K74, A:P75, A:I76 | Violet |
| **WP_003022843.1 (hypothetical protein)** | SYATELKGSDYISTEVGSTYNYQRVNADDQDK | 18-49 | 0.9382 | 100 | A:L4, A:I5, A:V6, A:T7, A:S8, A:T9, A:F10, A:I11, A:T12, A:A13, A:L14 | Red |
|  | EFNKIRNDNVTNAD | 116-129 | 1.0256 | 100 | A:N43, A:A44, A:D45, A:D46, A:Q47, A:D48 | Orange |
|  |  |  |  |  | A:D74, A:V75, A:N76, A:G77, A:K78, A:T79 | Cryan |
| **WP_003023105.1 (hypothetical protein)** | - | - | - |  | A:T4, A:L5, A:I6, A:T7 | Red |
|  |  |  |  |  | A:M8, A:V9, A:M10, A:L11, A:T12, A:S13, A:V14, A:G15, A:A16, A:A17, A:T18, A:N19, A:A20, A:S21, A:A22 | Yellow |
|  |  |  |  |  | A:G236, A:E237, A:P238, A:D239, A:E240, A:N241, A:G242, A:V243, A:Y244 | Orange |
|  |  |  |  |  | A:L53, A:C55, A:A59, A:V60, A:P61, A:P62, A:I132, A:N133, A:S134, A:K136, A:L137, A:S138, A:P139, A:Q140, A:G141, A:E142, A:Q143, A:E144, A:L145, A:K146, A:Y147, A:K150 | Cryan |
|  |  |  |  |  | A:Q206, A:D210, A:A211 | Violet |
| **WP_003023209.1 (DUF2147 domain-containing protein)** | LQRDSVTKT | 34-42 | 0.7794 | 100 | A:L2, A:N4, A:L5, A:I6, A:V7, A:I8, A:I9, A:V10, A:A11, A:V12, A:F13, A:V14, A:F15, A:S16, A:F17 | Red |
|  | NAKKTDNGNLAKGKGPLYTDG | 110-130 | 0.7448 | 100 | A:E194, A:D195, A:K196, A:N197, A:G198, A:K199, A:V200, A:N201, A:N202, A:K203, A:E204 | Orange |
|  |  |  |  |  | A:G84, A:K85, A:G86, A:D87, A:A88, A:G90, A:N91, A:L92, A:S96 | Green |
|  |  |  |  |  | A:G160, A:F161, A:G163 | Cryan |
|  |  |  |  |  |  |  |
|  |  |  |  |  |  |  |
| **WP_003023303.1 (outer membrane protein FopA)** | AYDKATLNAK | 283-292 | 0.885 | 100 | A:I21, A:A22, A:A23, A:G24, A:S25, A:D26, A:N27, A:I28, A:D29, A:T30, A:L31, A:A32, A:N33, A:T34, A:N35, A:S36, A:A37, A:T38, A:T39, A:S41, A:S42, A:G43 | Green |
|  |  |  |  |  | A:M184, A:A185, A:P186, A:S187, A:N188, A:I189, A:S190, A:G191, A:A192, A:N193, A:G194, A:R195 | Yellow |
|  |  |  |  |  | A:R2, A:L3, A:K4, A:S5, A:I6, A:V7, A:I8 | Cryan |
|  |  |  |  |  | A:T233, A:V234, A:A235, A:M236, A:P237, A:T238, A:I239, A:D240, A:E241, A:S242, A:K243, A:Y244, A:V245, A:L246, A:G271, A:D272, A:L388, A:K389, A:E390, A:A391 | Red |
|  |  |  |  |  | A:G87, A:T88, A:P89, A:N90, A:S91, A:A196 | Orange |
| **WP_003026145.1 (hypothetical protein)** | DKGVGEINNSSSVSPNNIAGV | 52-72 | 0.6682 | 100 | A:T108, A:V109, A:F110 | Red |
|  |  |  |  |  | A:N111, A:R112, A:V142, A:F143, A:Y144, A:G145, A:S146, A:Y173, A:L175, A:Y176, A:L177 | Yellow |
|  |  |  |  |  | A:M1, A:E2, A:T3, A:A4, A:G5, A:D6, A:I39, A:S40, A:D41 | Blue |
| **WP_003029346.1 (hypothetical protein)** | NHNAKLQANDTIKYEIKQKQNIPWKSL | 60-86 | 0.8526 | 100 | A:K3, A:I4, A:I5, A:A6, A:G7, A:V8, A:F9 | Red |
|  |  |  |  |  | A:F11, A:V12, A:F13, A:L14, A:I15, A:S16, A:N17, A:L18, A:Y19 | Violet |
|  |  |  |  |  | A:T97, A:N98, A:N99, A:S100, A:Y101, A:N102, A:N103, A:Y104, A:T105, A:K125, A:N126 | Yellow |
|  |  |  |  |  | A:A20, A:D21, A:L22, A:V23, A:A24 | Green |
|  |  |  |  |  | A:S40, A:D41, A:N42, A:V43, A:V44, A:D45, A:E74, A:I75, A:K76, A:Q77, A:K78, A:Q79, A:N80, A:I81, A:P82, A:W83, A:S85, A:L86, A:K95, A:E107, A:R113  A:D115, A:T116, A:S117, A:Y118 | Cryan  Blue |
|  |  |  |  |  | A:Y237, A:K238, A:G239, A:S258, A:K259, A:G260, A:Y261, A:S262, A:D263, A:Q264 | Orange |
| **WP_003029578.1 (hypothetical protein)** | AQQAVKIDVKDNPLRQYAAKSSIKSDSALVSENEVVSGSTIKQDQAPKANDYGD | 50-57 | 0.8341 | 100 | A:Q73, A:D74, A:Q75, A:A76, A:P77, A:K78 | Green |
|  |  |  |  |  | A:A79, A:N80, A:D81, A:Y82 | Pink |
|  | TYGGGT | 258-263 | 2.5336 | 100 |  |  |
|  |  |  |  |  | A:V65, A:V66, A:S67, A:G68, A:S69, A:T70, A:I71, A:K72 | Blue |
|  |  |  |  |  | A:V8, A:L9, A:T10, A:S11, A:F12 | purple |
|  |  |  |  |  | A:G83, A:D84, A:A85, A:L86, A:S87, A:S88, A:L89, A:V90, A:D91, A:T92, A:S93, A:D94, A:D95 | Red |
|  |  |  |  |  | A:D218, A:D219, A:E220, A:D221, A:S222, A:Q223, A:G224 | Yellow |
|  |  |  |  |  | A:K228, A:L229, A:S230, A:D231, A:D232, A:Y233, A:P234 | Orange |
| **WP_227644127.1 (carbohydrate-binding protein)** | - | - | - | - | A:K5, A:L6, A:I7, A:S8, A:S9, A:V10, A:T11, A:C12, A:A13, A:L14, A:M15, A:G16, A:M17, A:G18, A:T19, A:I20, A:S21, A:I22, A:A23, A:T24, A:A25, A:T26 | Red |
|  |  |  |  |  | A:P139, A:S140, A:K141, A:P142, A:E143, A:F144, A:V145, A:T146, A:D147, A:S148, A:S149, A:L150, A:W170 | Orange |
| **WP_003026358.1 (DUF3281 family protein)** | DTKTNK | 123-128 | 3.6525 | 100 | A:S14, A:A15, A:A16, A:L17, A:L18, A:E19, A:G20, A:C21, A:G22, A:K23, A:T24, A:E25, A:T26, A:T27, A:N28, A:E29, A:L30 | Red |
|  |  |  |  |  | A:D123, A:T124, A:K125, A:T126 | Orange |
